# Supplementary material for: Polyamines Control eIF5A Hypusination, TFEB Translation, and Autophagy to Reverse B Cell Senescence
Source: Mol Cell. 2019 Oct 3;76(1):110–125.e9. doi: 10.1016/j.molcel.2019.08.005 (PMC6863385; doi:10.1016/j.molcel.2019.08.005)
Supplement: Document S1. Figures S1–S7 [file mmc1.pdf]

Molecular Cell, Volume *76*

Supplemental Information

Polyamines Control eIF5A Hypusination, TFEB Translation, and Autophagy to Reverse B Cell Senescence

Hanlin Zhang, Ghada Alsaleh, Jack Feltham, Yizhe Sun, Gennaro Napolitano, Thomas Riffelmacher, Philip Charles, Lisa Frau, Philip Hublitz, Zhanru Yu, Shabaz Mohammed, Andrea Ballabio, Stefan Balabanov, Jane Mellor, and Anna Katharina Simon

Figure S1. Related to Figure 1. 6-week Treatment with Spermidine does not Affect Hematopoiesis in Old Mice.

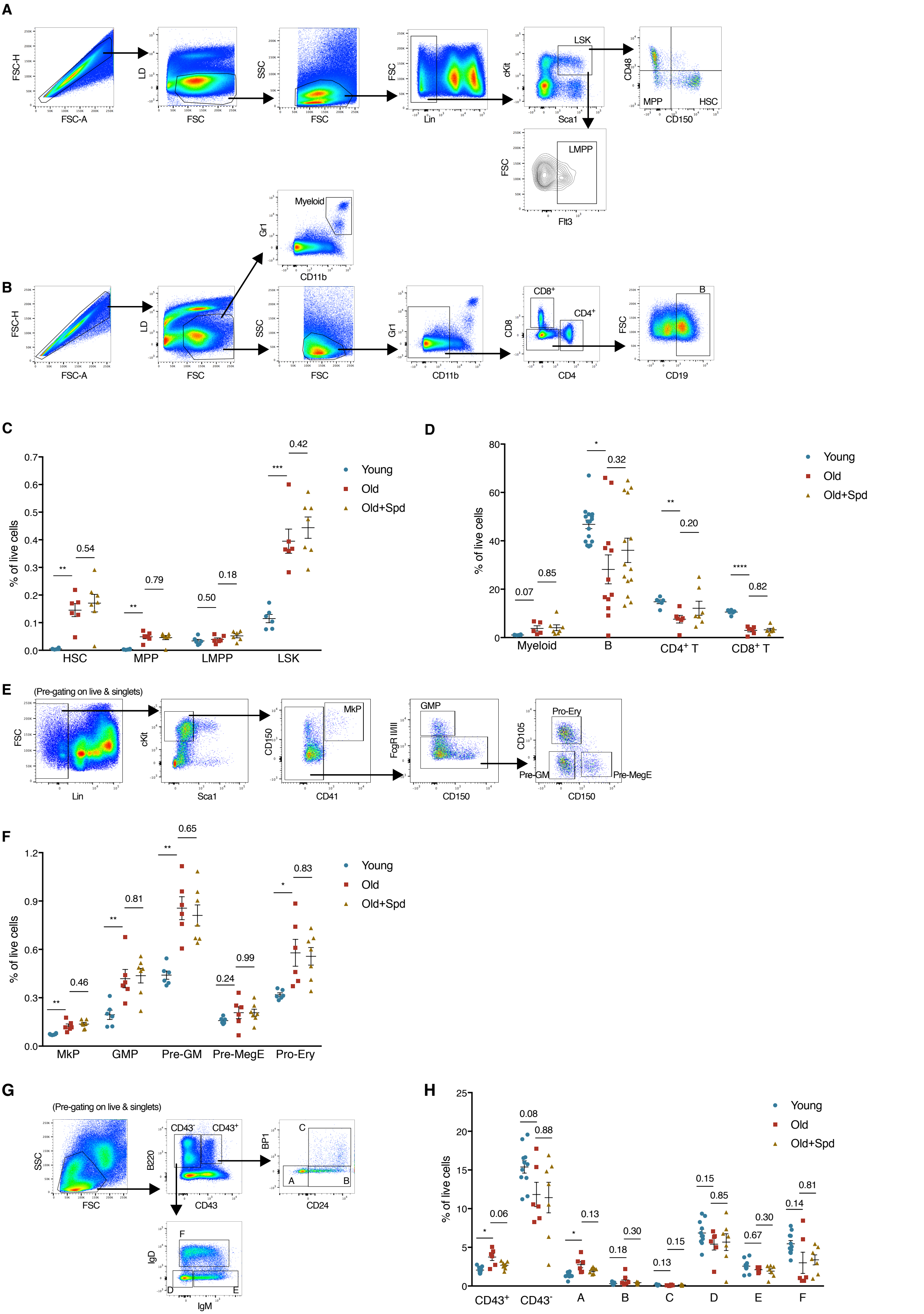

Figure S1. Related to Figure 1. 6-week Treatment with Spermidine does not Affect Hematopoiesis in Old Mice.

Various hematopoietic cell types in bone marrow and spleen from young (12 weeks), old (22-24 months), and old mice continuously administered with spermidine in drinking water for 6 weeks as in Figure 1A were assessed by flow cytometry.

(A) Gating strategy for hematopoietic stem cells (HSCs), multipotent progenitors (MPPs), lymphoid-biased multipotent progenitors (LMPPs), and $Lin^-Sca1^+cKit^+$ cells (LSKs, enriched of hematopoietic stem and progenitor cells) in bone marrow.

(B) Gating strategy for myeloid cells, B cells, $CD4^+$ T cells, and $CD8^+$ T cells in spleen.

(C) Expanded phenotypic HSCs, MPPs, and LSKs in bone marrow from old mice. The abundance of indicated cell types as % of total live cells is shown. n = 6-7 mice.

(D) Spleen lineages are lymphopenic in old mice. n = 6-17 mice as indicated by dots, combined from 2 independent experiments.

(E/F) Expanded myeloid progenitors in bone marrow from old mice. (E) Gating strategy for megakaryocyte progenitors (MkPs), granulocyte-macrophage progenitors (GMPs), pre-granulocyte/macrophages (Pre-GMs), pre-megakaryocytes/erythrocytes (pre-MegEs), and pro-erythroblast cells (Pro-Erys). (F) The abundance of indicated cell types is shown. n = 6-7 mice.

(G/H) Accumulated pro-B cells in old mice. (G) Gating strategy for Hardy fractions A-F. (H) The abundance of indicated cell types is shown. n = 7-11 mice, pooled from 2 independent experiments.

Each dot represents one mouse. Data represented as mean ± SEM. Welch's t-test. *P≤0.05, **P≤0.01, ***P≤0.001, ****P≤0.0001.

Figure S2. Related to Figure 2. Spermidine does not Improve B Cell Responses in Young Mice.

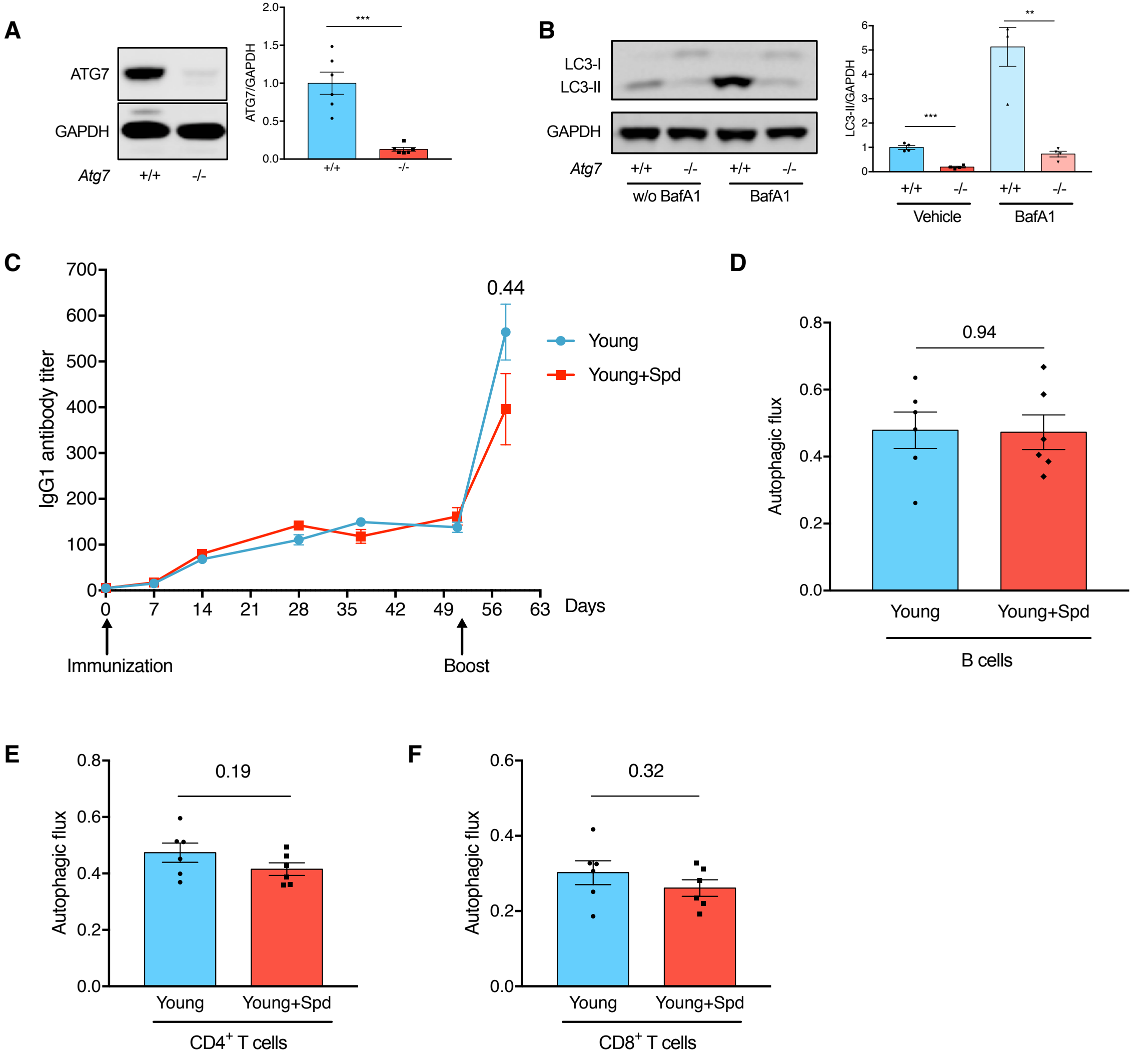

Figure S2. Related to Figure 2. Spermidine does not Improve B Cell Responses in Young Mice.

(A/B) Confirmation of reduced ATG7 expression and autophagy in B cells purified from B cell-specific *Atg7*-knockout mice. (A) B cells were purified from *Mb1-Cre-*, *Atg7*$^{flox/flox}$ (+/+) and *Mb1-Cre+*, *Atg7*$^{flox/flox}$ (-/-) mice and the expression of ATG7 was assessed by Western blot. n = 6 mice. (B) Purified B cells in (A) were cultured with 10 nM BafA1 for 2 h for LC3-II measurement by Western blot. n = 4 mice.

(C-F) Spermidine does not improve IgG1 responses in young mice. (C) Young adult mice were immunized and boosted with NP-CGG. Spermidine administration and serum NP-specific IgG1 measurement were processed as in Figure 2D. n = 11 (D7), 19 (D14), 12 (D28), 9 (D37), 15 (D51) and 6 (D58) mice, combined from 3 independent experiments. (D) Autophagic flux of splenic B cells (B220$^+$CD19$^+$) (D), CD4$^+$ T cells (E), and CD8$^+$ T cells (F) from mice culled on D58 in (C) was measured by LC3-II flow cytometry staining as in Figure 1A. n = 6 mice.

Data represented as mean ± SEM. One-tailed Welch's t-test (A/B). Two-tailed Student's t-test (C-F). $^{**}P \leq 0.01$, $^{***}P \leq 0.001$.

Figure S3. Related to Figure 3. Spermidine does not Directly Inhibit HAT Activities and High-dose Spermidine Induces Cellular Stress.

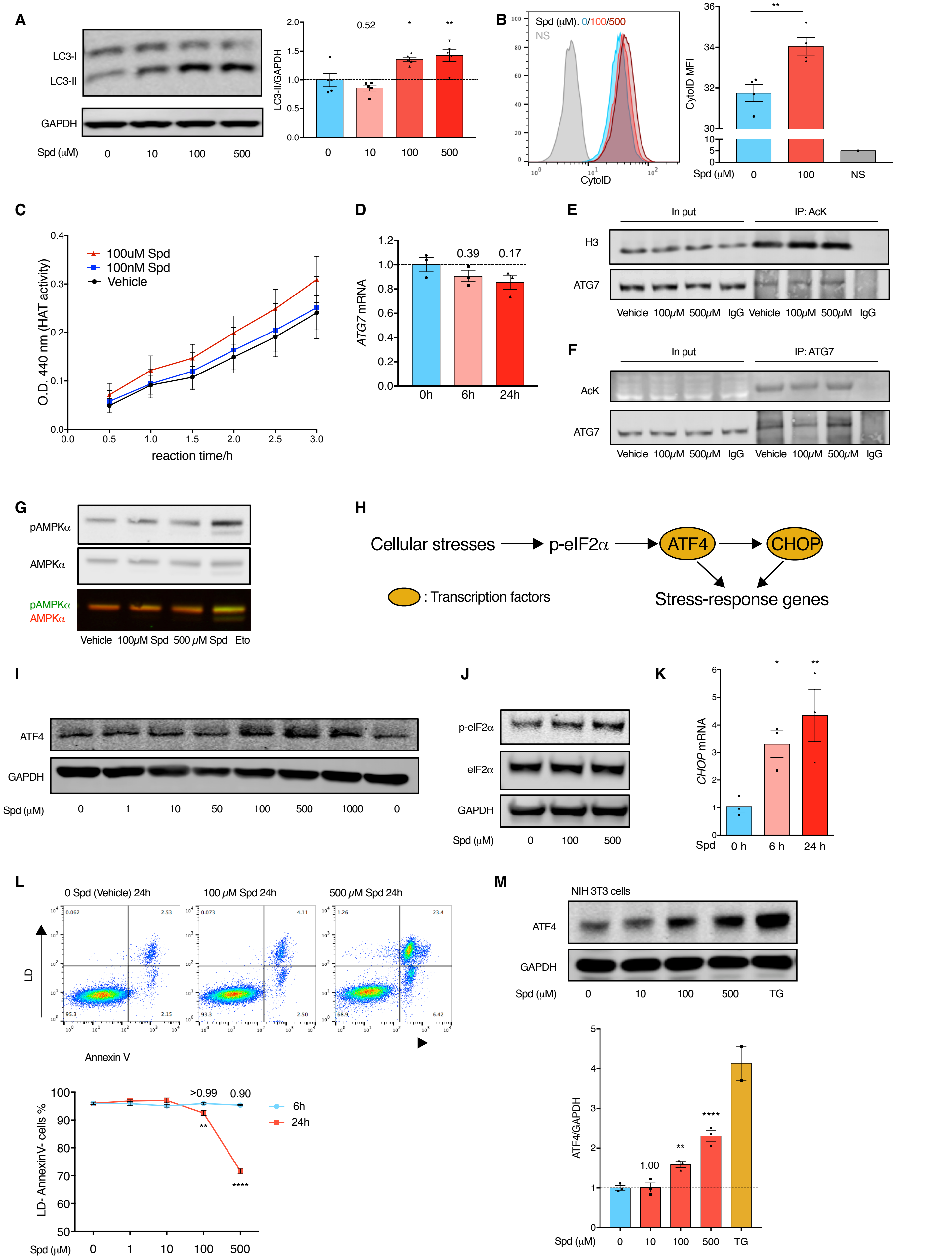

Figure S3. Related to Figure 3. Spermidine does not Directly Inhibit HAT Activities and High-dose Spermidine Induces Cellular Stress.

(A/B) Spermidine induces autophagy *in vitro*. (A) Jurkat cells were treated with spermidine as indicated for 6 h. Autophagy was assessed by LC3-II Western blot. n = 5. (B) Autophagosome/autolysosome-specific staining of CytoID was measured by flow cytometry. n = 4.

(C-F) Spermidine does not inhibit HAT activity in Jurkat cells. (C) Jurkat nuclear extract was prepared using the Nuclear/Cytosol Fractionation Kit, incubated with spermidine of indicated concentrations (starting from 0 h) and the relative HAT activity was measured using the HAT Activity Colorimetric Assay Kit. n = 3. (D) Jurkat cells were treated with 100 μM spermidine for 6 h or 24 h. *ATG7* mRNA was measured by quantitative PCR (qPCR) with *GAPDH* used as the reference gene. n = 3. (E/F) Jurkat cells were treated with spermidine for 6 h and cellular proteins with acetylated lysine residues were pulled down (IP: AcK) and assessed for H3 and ATG7 acetylation (E). To assess ATG7 acetylation in an alternative way, ATG7 was pulled down (IP: ATG7) and acetylation measured with an antibody against AcK (F).

(G) Spermidine does not affect AMPK activity. Jurkat cells were treated with spermidine or etoposide (Eto) for 6 h. AMPK activity was assessed by AMPKα phosphorylation. Representative of 3 independent repeats.

(H-M) High-dose spermidine induces cellular stress. (H) Schematic overview of the integrated stress response eIF2α-ATF4-CHOP pathway. Multiple cellular stresses induce the phosphorylation of eIF2α. Phosphorylated eIF2α facilitates the synthesis of the transcription factor ATF4, which then induces the expression of CHOP. ATF4 and CHOP induce the expression of multiple stress-response genes including chaperones, apoptosis, and autophagy. (I/J) Jurkat cells were treated with spermidine for 6 h. The expression of cellular stress markers ATF4 (I) and phosphorylation of eIF2α (J) were assessed by Western blot. Representative of 3 independent repeats. (K) Jurkat cells were treated with 100 μM spermidine. The expression of *CHOP* was assessed by qPCR with *GAPDH* as the reference gene. n = 3. (L) Jurkat cells were treated with spermidine of indicated concentrations for 6 h or 24 h. Cell viability and apoptosis were assessed by Live-Dead (LD) and Annexin V flow cytometry staining. n = 3. (M) NIH 3T3 cells were treated with spermidine or thapsigargin (TG) for 6 h. The expression of ATF4 was assessed by Western blot. n = 3.

Data represented as mean ± SEM. One-way ANOVA with post hoc Dunnett's test (A/D/K/M). Student's t-test (B). Two-way ANOVA with post hoc Dunnett's test (L). *P≤0.05, **P≤0.01, ****P≤0.0001.

Figure S4. Related to Figure 3. Spermidine Maintains Cellular Autophagy by Hypusinating eIF5A.

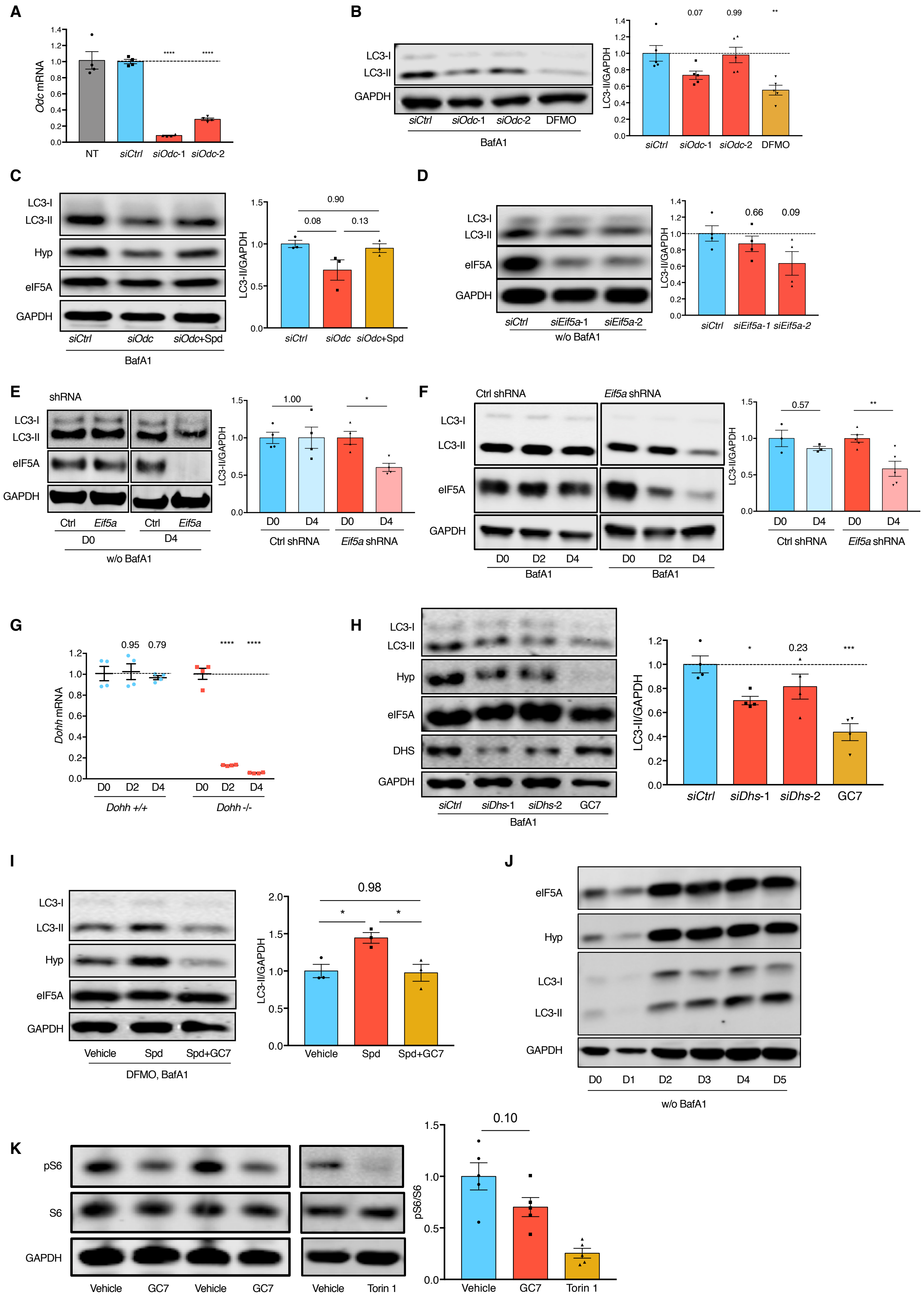

Figure S4. Related to Figure 3. Spermidine Maintains Cellular Autophagy by Hypusinating eIF5A.

(A) NIH 3T3 cells were transfected with non-targeting siRNA (*siCtrl*) or siRNA targeting two different regions of *Odc* mRNA (*siOdc*-1/2) for 3 days. The expression of *Odc* was assessed by qPCR with *Gapdh* as the reference gene. n = 4. NT, non-treatment. *siOdc-1* was used in all other figures unless specified otherwise.

(B-D) NIH 3T3 cells were transfected with *siOdc*-1/2 with/without 10 μM spermidine for 3 days (B/C), transfected with *siEif5a*-1/2 for 3 days (D), or treated with DFMO for 24 h (B). LC3-II was measured by Western blot. n = 3-5.

(E/F) The knockdown of *Eif5a* was induced by 100 μM IPTG in NIH 3T3 cells expressing IPTG-inducible *Eif5a* shRNA. The expression of LC3-II on indicated days post IPTG induction was measured by Western blot (E, without BafA1; F, with BafA1). n = 3-5.

(G) The expression of *Dohh* on indicated days post 4-OHT induced deletion was assessed by qPCR with *Gapdh* as the reference gene. n = 4.

(H) NIH 3T3 cells were transfected with *siCtrl* or siRNA targeting two different regions of *Dhs* mRNA (*siDhs*-1/2) for 3 days or treated with 100 μM GC7 for 24 h. n = 4. *siDhs-1* was used in all other figures unless specified otherwise.

(I) Spermidine-depleted NIH 3T3 cells by DFMO treatment were rescued with 10 μM spermidine alone or spermidine together with GC7 for 24 h. LC3-II was measured by Western blot. n = 3.

(J) Purified murine B cells were cultured with LPS for indicated days as in Figure 3H. The expression of overall eIF5A, hypusinated eIF5A, and LC3-II (without BafA1 treatment) was assessed by Western blot. Representative of 4 independent repeats.

(K) GC7 inhibits autophagy in an mTOR-independent manner (not via activating mTOR). LPS-activated murine B cells were treated with GC7 for 24 h as in Figure 3I, or with the mTOR inhibitor Torin 1 for 2 h. The expression of S6 and its phosphorylation (Ser235/236) downstream of mTOR were assessed by Western blot. n = 5.

BafA1 was added 2 h before harvest where indicated for autophagy measurement. Data represented as mean ± SEM. One-way ANOVA with post hoc Dunnett's test (A/B/D/H), or with post hoc Tukey's test (C/I). Two-way ANOVA with post hoc Sidak's test (E/F) or Dunnett's test (G), Student's t-test (K). *$P \leq 0.05$, **$P \leq 0.01$, ***$P \leq 0.001$, ****$P \leq 0.0001$.

Figure S5. Related to Figure 4 and Figure 5. Hypusinated eIF5A Regulates TFEB Expression and Overall Protein Synthesis.

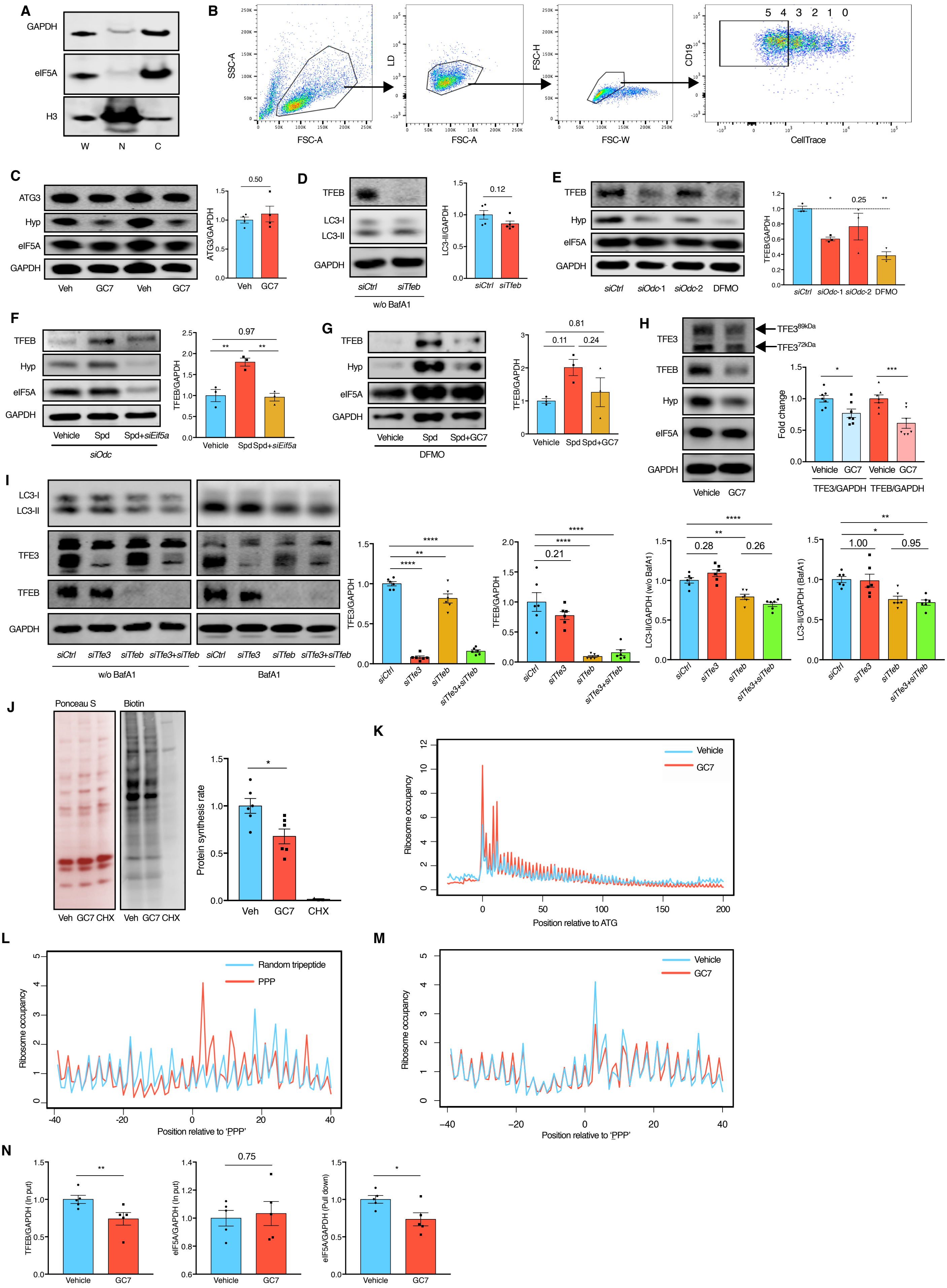

Figure S5. Related to Figure 4 and Figure 5. Hypusinated eIF5A Regulates TFEB Expression and Overall Protein Synthesis.

(A) An aliquot of protein samples for MS in Figure 4A was assessed by Western blot for cell fractionation efficiency. W: whole cell lysate; N: nuclear fraction; C: cytoplasmic fraction.

(B) Gating strategy for the FACS sorting in Figure 4B. Live B cells ($CD19^+$) that divided four times or more were collected.

(C) Murine B cells were treated with 10 μM GC7 for 24 h as in Figure 3I. The expression of ATG3 was assessed by Western blot. n = 4 mice.

(D) NIH 3T3 cells were transfected with *siTfeb* for 3 days. LC3-II expression was measured by Western blot. n = 5.

(E) NIH 3T3 cells were transfected with *siOdc*-1/2 for 3 days or treated with 1 mM DFMO for 24 h. TFEB expression was measured by Western blot. n = 3.

(F) Spermidine-depleted NIH 3T3 cells by *siOdc* transfection were rescued with spermidine alone or in combination with *siEif5a* for 3 days. n = 3.

(G) DFMO-treated NIH 3T3 cells were treated (rescued) with spermidine alone or spermidine together with GC7 for 24 h. n = 3.

(H) Murine B cells were treated with GC7 as in (C). The expression of TFE3 and TFEB was assessed by Western blot. n = 7 mice.

(I) NIH 3T3 cells were transfected with *siTfe3*, *siTfeb*, or both together for 3 days. The expression of TFE3, TFEB, and LC3-II was assessed by Western blot. n = 6.

(J) Murine B cells were treated with GC7 as in (C). Nascent proteins were labeled with AHA for 4 h, conjugated to biotin by click reaction and directly assessed by Western blot with IRDye-conjugated streptavidin. Ponceau S staining of total proteins was used as the loading control. Cycloheximide (CHX) was added 30 min before adding AHA to inhibit translation as the positive control. n = 6 mice.

(K-M) Murine B cells were treated with GC7 as in (C) and processed for ribosome profiling. (K) Ribosome occupancy of all genes is aligned at the start codon and normalized to a mean value of 1 for each gene. (L/M) Ribosome occupancy of all genes expressing PPP motifs of the Vehicle sample (L) or GC7 and Vehicle samples (M) is aligned at the underlined Pro on the P site of the ribosome and normalized to a mean value of 1 for each gene.

(N) Quantification of Figure 5B. n = 5 mice.

Data represented as mean ± SEM. Student's t-test (C/D/J/N). One-way ANOVA with post hoc Dunnett's test (E/I TFE3, TFEB), Tukey's test (F/G/I LC3-II), or Sidak's test (H). *$P \leq 0.05$, **$P \leq 0.01$, ***$P \leq 0.001$, ****$P \leq 0.0001$.

Figure S6. Related to Figure 6. Hypusination of eIF5A is Essential for Hematopoiesis and B Cell Activation.

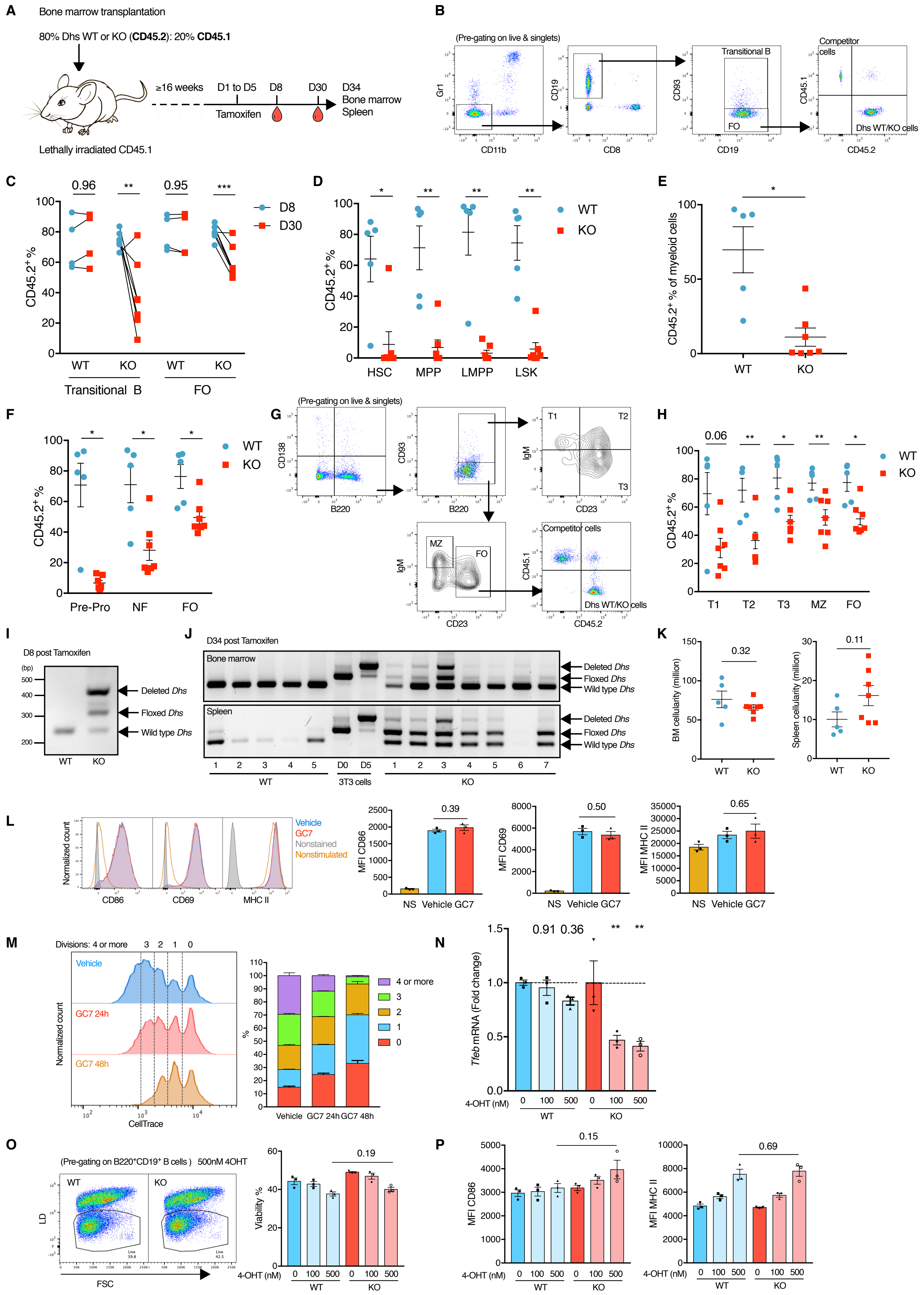

Figure S6. Related to Figure 6. Hypusination of eIF5A is Essential for Hematopoiesis and B Cell Activation.

(A-K) Competitive bone marrow chimeric mice were generated by transplanting bone marrow cells from tamoxifen-inducible CD45.2$^+$ *Dhs* knockout mice (WT: *CAG-Cre/Esr1*$^+$, *Dhs*$^{+/+}$; KO: *CAG-Cre/Esr1*$^+$, *Dhs*$^{f/f}$,) and wild type CD45.1$^+$ competitors into CD45.1$^+$ recipient mice (A). After ≥ 16 weeks of long-term reconstitution, tamoxifen was administered by oral gavage for 5 consecutive days, followed with lineage contribution assessment. (B/C) The contribution of CD45.2$^+$ cells to transitional (CD19$^+$CD93$^+$) and mature follicular (FO) B cells (CD19$^+$CD93$^-$) in peripheral blood on day 8 and day 30 post tamoxifen induction was assessed by flow cytometry. (D-H) The contribution of CD45.2$^+$ cells to bone marrow hematopoietic stem and progenitor cells (D), spleen myeloid cells (CD11b$^+$Gr1$^+$) (E), bone marrow pre-pro B cells (Hardy fraction A), newly formed B cells (NF, fraction E), follicular B cells (FO, fraction F) (F), and spleen transitional B cells (T1-3), marginal zone B cells (MZ), follicular B cells (G/H) was assessed by flow cytometry on day 34 post tamoxifen induction. (I/J) *Dhs* deletion efficiency was tested by PCR. DNA was extracted from pooled peripheral blood of the chimeric mice on day 8 post tamoxifen administration (I) or from bone marrow and spleen on day 34 post tamoxifen administration (J). 4-OHT-induced *Dhs* deletion (D0/D5) in 3T3 cells derived from *CAG-Cre/Esr1*$^+$, *Dhs*$^{f/f}$ mouse embryonic fibroblasts was used as positive control. (K) The cellularity of bone marrow (tibia and femur of both sides) and spleen of WT and KO *Dhs* chimeric mice. n = 4-7 mice.

(L) Wild type murine B cells were stimulated with LPS for 1 day with or without 10 μM GC7. The expression of early activation markers CD86, CD69, and MHC II was assessed by flow cytometry. NS, nonstimulated. n = 3 mice.

(M) Wild type murine B cells were stained with CellTrace and cultured with LPS for 3 days. GC7 was added 24 h or 48 h before harvest for cell proliferation analysis (left). The percentage of cells that divided for the indicated times is quantified (right). n = 6 mice.

(N-P) The knockout of *Tfeb* in B cells was induced as in Figure 6A for 4 days. (N) The *Tfeb* knockout efficiency was assessed by qPCR with *Gapdh* as the reference gene. (O) The viability of B cells was assessed by flow cytometry. (P) Quantification of Figure 6C. n = 3 mice.

Data represented as mean ± SEM. Two-way ANOVA with post hoc Sidak's test for transitional B cells and follicular B cells separately (C) or with post hoc Dunnett's test (N). Welch's t-test (D/E/F/H). Student's t-test (K/L/O/P). *P≤0.05, **P≤0.01, ***P≤0.001.

Figure S7. Related to Figure 7. Spermidine does not Improve Young Human B Cell Responses.

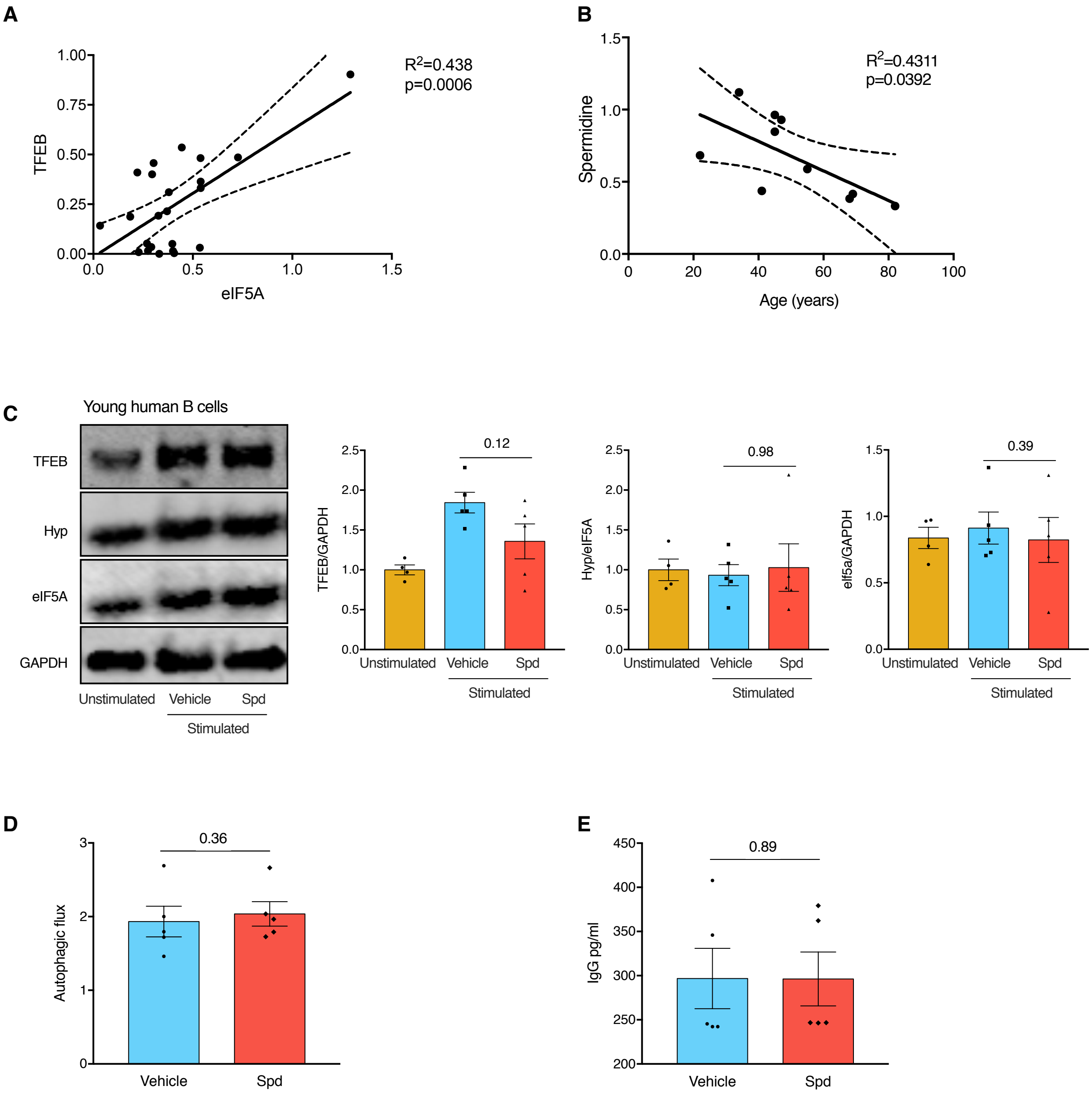

Figure S7. Related to Figure 7. Spermidine does not Improve Young Human B Cell Responses.

(A) Correlative analysis between the expression of TFEB and eIF5A (both normalized to GAPDH) in samples of Figure 7A. n = 23 donors.

(B) Spermidine content of PBMCs collected from healthy donors was measured by GC-MS. n = 10 donors.

(C-E) Sorted B cells from young human donors (<65 years) were stimulated by anti-IgM and CD40L for 7 days with 10 μM spermidine where indicated. (C) The expression of TFEB, overall eIF5A and hypusinated eIF5A was assessed by Western blot. (D) The autophagic flux was measured by flow cytometry staining of LC3-II as in Figure 1A. (E) Supernatant IgG was assessed by ELISA. n = 5 donors. Data represented as mean ± SEM.

Linear regression with 95% confidence intervals (A/B). The goodness of fit is assessed by R^2, and the P value of the slope (whether significantly non-zero) is calculated by F test (A/B). Paired Student's t-test (C-E).
