# Supplementary material for: Polyamines Control eIF5A Hypusination, TFEB Translation, and Autophagy to Reverse B Cell Senescence
Source: Mol Cell. 2019 Oct 3;76(1):110–125.e9. doi: 10.1016/j.molcel.2019.08.005 (PMC6863385; doi:10.1016/j.molcel.2019.08.005)
Supplement: Document S2. Article plus Supplemental Information [file mmc2.pdf]

Article

Molecular Cell

Polyamines Control eIF5A Hypusination, TFEB Translation, and Autophagy to Reverse B Cell Senescence

Graphical Abstract

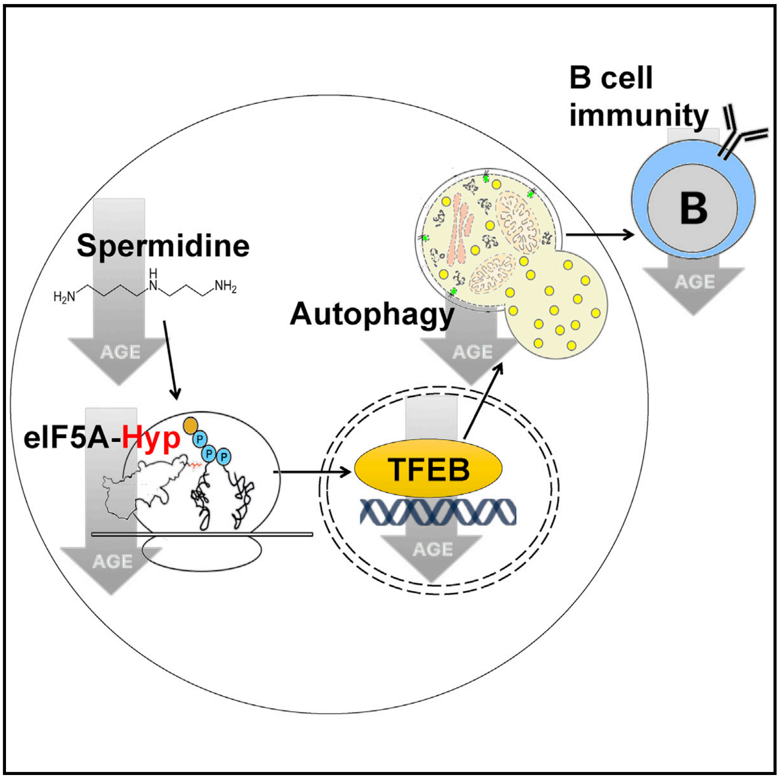

Highlights

- The endogenous polyamine spermidine maintains autophagy via hypusinating eIF5A
- Hypusinated translation factor eIF5A controls TFEB expression and autophagy
- Spermidine restores B cell responses in old mice and humans
- TFEB levels decline with age and can be used as drug target/ biomarker for human aging

Authors

Hanlin Zhang, Ghada Alsaleh, Jack Feltham, ..., Stefan Balabanov, Jane Mellor, Anna Katharina Simon

Correspondence

katja.simon@kennedy.ox.ac.uk

In Brief

In this study, Zhang et al. restore B cell immunity in the elderly with spermidine, a cellular polyamine metabolite depleted with age. They find that autophagy is maintained by spermidine via promoting eIF5A function and TFEB translation. This pathway provides an explanation for the age-related decline of autophagy.

Zhang et al., 2019, Molecular Cell *76*, 110–125
October 3, 2019 © 2019 The Author(s). Published by Elsevier Inc.
https://doi.org/10.1016/j.molcel.2019.08.005

Polyamines Control eIF5A Hypusination, TFEB Translation, and Autophagy to Reverse B Cell Senescence

Hanlin Zhang,[1] Ghada Alsaleh,[1,9] Jack Feltham,[2,9] Yizhe Sun,[1] Gennaro Napolitano,[3,4] Thomas Riffelmacher,[1] Philip Charles,[2,5] Lisa Frau,[1] Philip Hublitz,[6] Zhanru Yu,[5] Shabaz Mohammed,[2] Andrea Ballabio,[3,4,7] Stefan Balabanov,[8] Jane Mellor,[2] and Anna Katharina Simon[1,10,*]
[1]The Kennedy Institute of Rheumatology, University of Oxford, Roosevelt Drive, Oxford, OX3 7FY, UK
[2]Department of Biochemistry, University of Oxford, South Parks Road, Oxford, OX1 3QU, UK
[3]Telethon Institute of Genetics and Medicine (TIGEM), Via Campi Flegrei 34, 80078, Pozzuoli, Naples, Italy
[4]Medical Genetics Unit, Department of Medical and Translational Science, Federico II University, Via Pansini 5, 80131, Naples, Italy
[5]Target Discovery Institute, University of Oxford, Roosevelt Drive, Oxford, OX3 7FZ, UK
[6]MRC Molecular Haematology Unit, Weatherall Institute of Molecular Medicine, John Radcliffe Hospital, Oxford, OX3 9DS, UK
[7]Department of Molecular and Human Genetics and Neurological Research Institute, Baylor College of Medicine, Houston, TX 77030, USA
[8]Division of Haematology, University Hospital and University of Zürich, 8091, Zürich, Switzerland
[9]These authors contributed equally
[10]Lead Contact
*Correspondence: katja.simon@kennedy.ox.ac.uk
https://doi.org/10.1016/j.molcel.2019.08.005

SUMMARY

Failure to make adaptive immune responses is a hallmark of aging. Reduced B cell function leads to poor vaccination efficacy and a high prevalence of infections in the elderly. Here we show that reduced autophagy is a central molecular mechanism underlying immune senescence. Autophagy levels are specifically reduced in mature lymphocytes, leading to compromised memory B cell responses in old individuals. Spermidine, an endogenous polyamine metabolite, induces autophagy *in vivo* and rejuvenates memory B cell responses. Mechanistically, spermidine post-translationally modifies the translation factor eIF5A, which is essential for the synthesis of the autophagy transcription factor TFEB. Spermidine is depleted in the elderly, leading to reduced TFEB expression and autophagy. Spermidine supplementation restored this pathway and improved the responses of old human B cells. Taken together, our results reveal an unexpected autophagy regulatory mechanism mediated by eIF5A at the translational level, which can be harnessed to reverse immune senescence in humans.

INTRODUCTION

Immune senescence is characterized by the failure of lymphocytes to respond adequately to infection, malignancy, and vaccination. During a regular influenza season, about 90% of the deaths occur in people older than 65 years (Yoshikawa, 2000). Immune responses to vaccines are known to be particularly ineffective in the elderly population (>65 years of age), and yet some vaccines, such as those for influenza, are given primarily to the elderly. A major correlate of protection for vaccinations is the specific antibody titer generated by long-lived plasma B cells. With a lifespan of several decades, long-lived lymphocytes are particularly prone to accumulation of intracellular waste. Autophagy recycles unwanted cytoplasmic material. Autophagy-deficient lymphocytes are unable to generate adequate responses, in particular long-lived lymphocytes, memory T and B cells, and plasma B cells (Chen et al., 2014; Pengo et al., 2013; Puleston et al., 2014; Xu et al., 2014). Reversing immune aging would also open opportunities to improve management of age-related morbidities. However, little is known about how immune senescence can be reversed.

Genetic activation of autophagy extends lifespan in mice (Fernández et al., 2018; Pyo et al., 2013). However, only a few autophagy-inducing drugs reverse aging, one being rapamycin, an inhibitor of mTOR (Bjedov et al., 2010). Because of the unwanted effects of mTOR inhibition, there is a need to better understand mTOR-independent control of autophagy for drug development. Furthermore, to test anti-aging drugs, blood biomarkers are critical. As an endogenous polyamine metabolite that declines with age, spermidine may be key in controlling cellular aging via autophagy (Eisenberg et al., 2009). Previously we found in aged mice that spermidine improves memory T cell responses (Puleston et al., 2014), and Eisenberg et al. (2016) showed cardiac function was restored with spermidine, both in an autophagy-dependent manner. Here we investigated whether spermidine is able to rejuvenate long-term B cell responses, the main correlate of protection for vaccination. We found an autophagy-dependent improvement of B cell responses by spermidine in old mice, indicating an effect across different immune cell types. We further identified the mechanism by which it regulates autophagy.

 © 2019 The Author(s). Published by Elsevier Inc.
This is an open access article under the CC BY license (http://creativecommons.org/licenses/by/4.0/).

Spermidine post-translationally modifies (hypusinates) eIF5A, which regulates protein synthesis and autophagy in primary B cells. Because of the rigid structure of proline, triprolines slow down translation and require hypusinated eIF5A to form peptide bonds more effectively (Dever et al., 2014). We find that this is specifically required for the synthesis of the autophagosomal and lysosomal master regulator TFEB (Settembre et al., 2011), which is a short-lived protein containing one triproline motif in mouse and two in human. Moreover, this pathway is considerably downregulated in immune cells from old individuals, making its components suitable biomarkers. Importantly, spermidine improves hypusination of eIF5A, TFEB protein expression, autophagic flux, and antibody titers in old human B cells, which has direct translational relevance.

RESULTS

Six-Week *In Vivo* Treatment with Spermidine Induces Autophagy but Does Not Affect Hematopoiesis in Old Mice

Previously, we demonstrated that autophagic flux is decreased with age in human and murine T cells (Phadwal et al., 2012; Puleston et al., 2014). Here, we first investigated the extent of the decrease across bone marrow progenitors and splenic mature hematopoietic cells, with a method of quantifying endogenous LC3-II, a marker for autophagosomes, using flow cytometry that we adapted for primary hematopoietic cells (Cossarizza et al., 2017). To measure active autophagic flux, we used bafilomycin A1 (BafA1), which is a lysosomal V-ATPase inhibitor that prevents acidification of lysosomes and their degradative functions, thereby causing accumulation of autophagic substrates, including LC3-II (Figure 1A). Hematopoietic stem cells (HSCs) were found to have the highest basal autophagy levels compared with other hematopoietic cells. Although their autophagic flux was mildly reduced in old mice, it was significantly decreased in B and T cells from old mice as compared to young mice (Figure 1A; gating strategy in Figures S1A and S1B). Treatment of mice for 6 weeks with spermidine increased autophagic flux in most hematopoietic cell types tested (Figure 1A). We then tested if the *in vivo* administration of spermidine alleviates typical hallmarks of hematopoietic aging, including expansion of phenotypic HSCs, myeloproliferation, and lymphopenia (Henry et al., 2011). We observed a significant increase of phenotypic HSCs and $Lin^-Sca1^+cKit^+$ cells (LSKs) in old mice, which spermidine administration did not affect (Figures 1B and S1C). A myeloid-biased phenotype, including increased myeloid cells and more significantly diminished mature B and T cells, was found in spleens of old mice (Figures 1C and S1D). Consistently, multiple bone marrow myeloid progenitors are expanded (Figures 1D, S1E, and S1F). Bone marrow pro-B cells ($CD43^+$) also mildly accumulate (Figures 1E, 1F, S1G, and S1H), in line with the paradigm that pro-B cell maturation is blocked with age (Klinman and Kline, 1997). Six weeks of treatment with spermidine does not affect these aging phenotypes in either spleen or bone marrow (Figures 1C–1F and S1D–S1H), although longer treatment may confer a more significant effect, as lifespan extension has previously been demonstrated with 6 month treatment (Eisenberg et al., 2016).

Spermidine Restores B Cell Responses in Old Mice

Consistent with the flow cytometry staining of LC3-II (Figure 2A), reduced autophagic flux was confirmed in old mature B lymphocytes by western blot (Figure 2B) and by confocal microscopy using GFP-LC3 transgenic mice (Figure 2C). Old B lymphocytes accumulated LC3-II, which did not further increase by BafA1 treatment (Figures 2A–2C). This indicates that autophagic flux is impaired in old cells at the level of the lysosome.

We then examined whether spermidine improves B cell function. In mice older than 22 months, the antibody response to immunization with the model antigen NP-CGG was markedly reduced, as expected, and administration of spermidine in drinking water significantly improved IgG1 responses (Figure 2D). In contrast, *Atg7*-deleted B cells showed defective memory responses that mimic the aging phenotype, but spermidine failed to promote their responses (Figures 2E, S2A, and S2B). However, it should be noted that this does not exclude alternative rejuvenation mechanisms of spermidine in addition to autophagy induction. At the time of sacrifice, we found very small numbers of long-lived bone marrow NP-specific plasma cells in old mice, which were significantly restored with spermidine treatment (Figure 2F). Spermidine did not induce autophagy in B cells or T cells or improve the antibody responses in young mice (Figures S2C–S2F). Thus, spermidine restored autophagic flux and the responses of B lymphocytes in old mice *in vivo*, and its immune-boosting effect is lost in autophagy-deficient B cells.

Spermidine Maintains Cellular Autophagy by Hypusinating eIF5A

We next investigated how spermidine regulates autophagy. First, we confirmed 100 μM spermidine to be the optimal concentration to induce autophagy in the mammalian lymphocytic Jurkat cell line (Figures S3A and S3B; Eisenberg et al., 2009; Morselli et al., 2011). Several signaling pathways have been described downstream of spermidine, including inhibition of histone acetyltransferases (HATs) (Madeo et al., 2018). Therefore, we assessed whether spermidine inhibits HAT activity and thereby induces *ATG7* mRNA expression, as shown in yeast (Eisenberg et al., 2009). In none of three approaches (HAT colorimetric assay, *ATG7* qPCR, or acetylated H3 pull-down) did we find that spermidine had this effect (Figures S3C–S3E). The acetyltransferase p300 has been shown to directly acetylate certain autophagy proteins, such as ATG7 to inhibit autophagy (Lee and Finkel, 2009), and spermidine was reported to inhibit p300 activity (Pietrocola et al., 2015). However, ATG7 acetylation was not affected by two tested concentrations of spermidine within 6 h of treatment (Figures S3E and S3F). Spermidine also failed to affect the activity of AMPK, as assessed using AMPK phosphorylation (Figure S3G). However, we found that spermidine induces cellular stress (Figure S3H) at the autophagy-inducing dose of 100 μM or higher, demonstrated by increased levels of ATF4 (Figure S3I), phosphorylated eIF2α (Figure S3J), and increased expression of *CHOP* mRNA (Figure S3K) in Jurkat cells. Furthermore, high-dose spermidine induced cell death after 24 h (Figure S3L), presumably because spermidine levels are high in transformed cell lines, and further uptake is toxic and non-specifically induces cellular stress responses, likely also inducing autophagy. Cellular stress was also induced by spermidine in

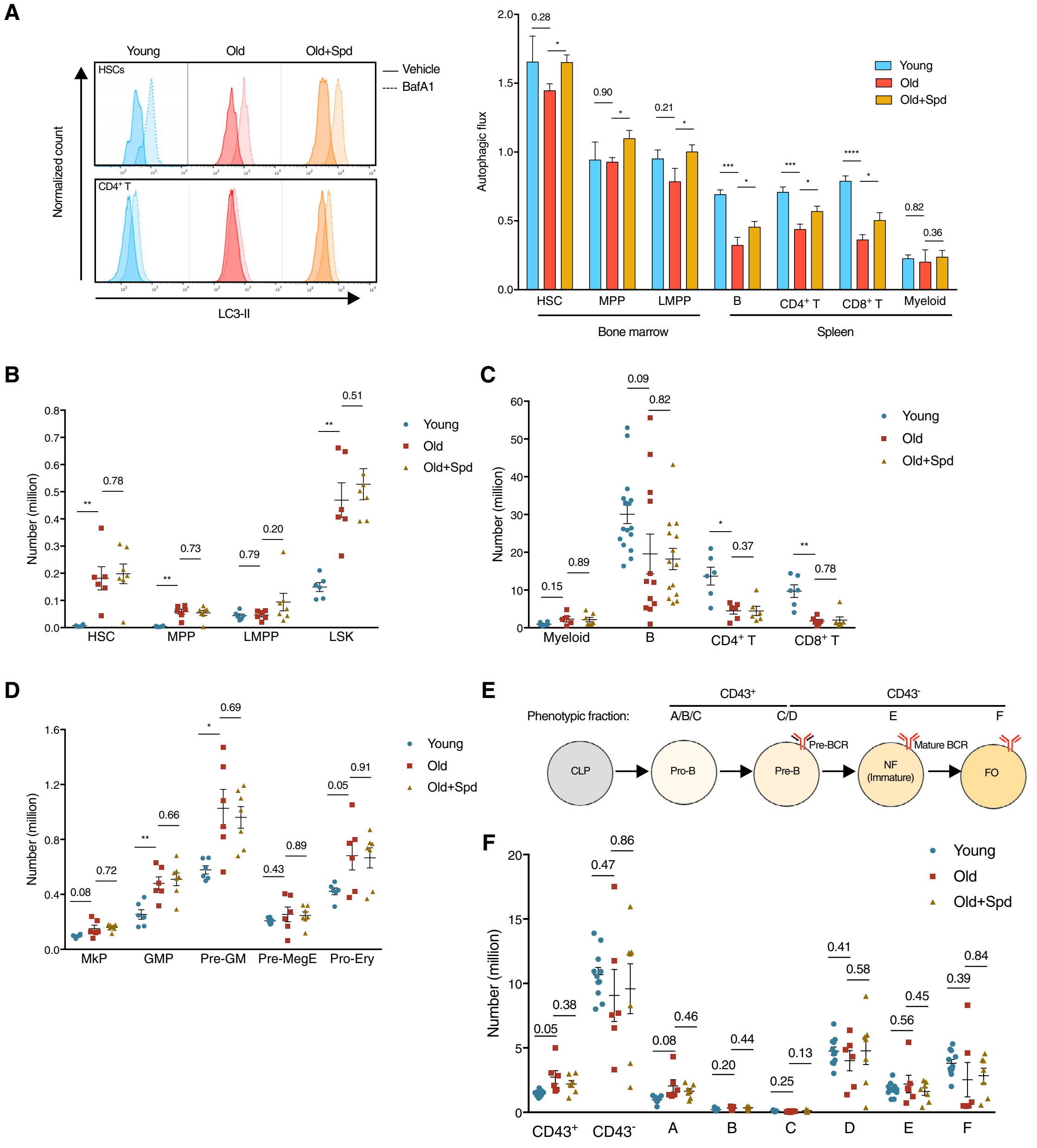

Figure 1. Six-Week *In Vivo* Treatment with Spermidine Induces Autophagy but Does Not Affect Hematopoiesis in Old Mice

(A) The autophagic flux of indicated cell types from young mice (12 weeks), old mice (24 months), and old mice administered spermidine (Spd) for 6 weeks was measured using LC3-II staining using flow cytometry after 2 h treatment with bafilomycin A1 (BafA1). Representative LC3-II plots of hematopoietic stem cells (HSCs) and CD4$^+$ T cells are shown (left). Autophagic flux was calculated as LC3-II geometric mean fluorescence intensity: (BafA1-Vehicle)/Vehicle (right). LMPP, lymphoid-biased multipotent progenitor; MPP, multipotent progenitor. n = 5, 6, and 7 mice for young, old, and old + Spd, respectively.

(B–F) Absolute count of indicated cell types in mice treated as in (A).

(B) Expanded hematopoietic stem and progenitor cells in old mice. LSK, Lin$^-$Sca1$^+$cKit$^+$ cell. n = 6 or 7 mice.

(C) Old mice are lymphopenic (spleen). n = 6–17 mice.

(D) Expanded myeloid progenitors in bone marrow of old mice. GMP, granulocyte-macrophage progenitor; MkP, megakaryocyte progenitor; Pre-GM, pre-granulocyte/macrophage; Pre-MegE, pre-megakaryocyte/erythrocyte; Pro-Ery, pro-erythroblast cell. n = 6 or 7 mice.

(E) Hardy fractions (A–F) and their correlation with B cell developmental stages. CLP, common lymphoid progenitor; FO, follicular B cell (mature recirculating B cell); NF, newly formed B cell; Pre-B, precursor B cell; Pro-B, progenitor B cell.

(F) B cell development is mildly blocked at the pro-B cell stage in old mice. n = 7–11 mice.

Data are represented as mean ± SEM. Two-tailed Student's t test for comparison of young versus old; one-tailed Student's t test for comparison of old versus old + Spd (A). Two-tailed Welch's t test (B–F). *p ≤ 0.05, **p ≤ 0.01, ***p ≤ 0.001, and ****p ≤ 0.0001.

See also Figure S1.

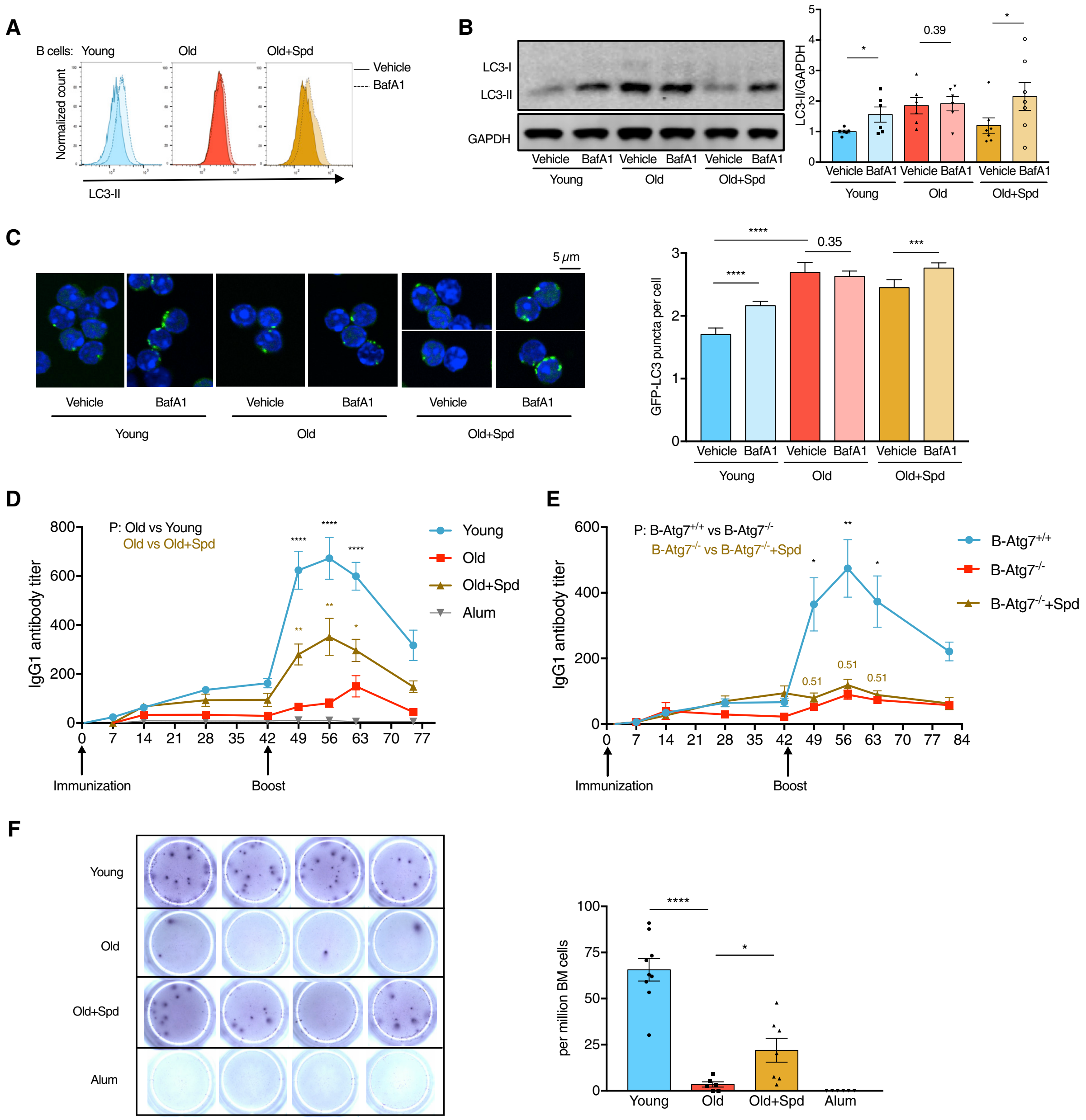

Figure 2. Spermidine Restores B Cell Responses in Old Mice

(A) Representative plot of LC3-II staining of B cells (CD19$^+$) from Figure 1A.

(B) Autophagy (LC3-II) of purified B cells from wild-type mice (treated as in Figure 1A) was assessed using western blot. n = 6 or 7 mice.

(C) Old GFP-LC3 transgenic mice were administered spermidine as in Figure 1A. The GFP-LC3 puncta of purified B cells were measured using confocal microscopy. N = 210–616 cells from four to six mice.

(D) Young or old mice were immunized with NP-CGG and administered spermidine throughout the experiment. Serum NP-specific IgG1 titers were measured using ELISA. n = 14 (young) or 10 (old/old + spd) mice from two experiments.

(E) B cell-specific *Atg7*-KO mice (*Mb1-Cre*, *Atg7$^{f/f}$*) were immunized and IgG1 responses assessed as in (D). n = 7 (B-Atg7$^{+/+}$) or n = 3 (B-Atg7$^{-/-}$) mice.

(F) Mice from (D) were culled on day 75, and bone marrow plasma cells secreting NP-specific IgG1 were measured using ELISpot. n = 6–9 mice.

Data are represented as mean ± SEM. Paired one-tailed Student's t test (B). Mann-Whitney test (C). Welch's t test (D–F). p values were adjusted using the Holm-Sidak method for multiple comparisons of the three time points in (D) and (E). *$p \leq 0.05$, **$p \leq 0.01$, ***$p \leq 0.001$, and ****$p \leq 0.0001$.

See also Figure S2.

NIH 3T3 cells as assessed by increased ATF4 expression (Figure S3M), indicating that high-dose spermidine induces cellular stress across different cell types.

Therefore, in subsequent experiments, rather than adding spermidine, we opted for the depletion of spermidine either by genetic knockdown (KD) of the key spermidine-synthesizing

enzyme, ornithine decarboxylase (ODC), or with the ODC inhibitor difluoromethylornithine (DFMO) in NIH 3T3 cells (Figure 3A). Indeed, genetic KD of *Odc* with small interfering RNA (siRNA) (Figure S4A), or DFMO treatment, reduces spermidine levels substantially, while supplementing cells with spermidine rescues its levels partially, as measured using gas chromatography-mass spectrometry (GC-MS) in cell lysates (Figure 3B). This demonstrates, first, that cultured cells actively synthesize spermidine via ODC and, second, that exogenous spermidine is efficiently taken up to rescue its intracellular levels. Next, we examined pathways downstream of spermidine that had not been previously linked to autophagy. In eukaryotic cells, spermidine is a unique substrate for the hypusination of the translation factor eIF5A (Figure 3A; Rossi et al., 2014). To date, eIF5A is the only known protein containing the unusual amino acid hypusine (Dever et al., 2014). Upon KD of *Odc* in NIH 3T3 cells, or addition of DFMO, we found reduced LC3-II levels and reduced eIF5A hypusination, while supplementation with spermidine rescued both (Figures 3C, S4B, and S4C). Similarly, reduced LC3-II levels were observed when *Eif5a* was knocked down (Figures 3D and S4D–S4F). We next depleted the two hypusinating enzymes, deoxyhypusine synthase (DHS) and deoxyhypusine hydroxylase (DOHH), to study their effects on autophagy. The KD of *Dhs* and knockout (KO) of *Dohh* (Pällmann et al., 2015), or GC7, a specific inhibitor of DHS, reduced eIF5A hypusination and LC3-II levels (Figures 3E, S4G, and S4H). Although exogenous spermidine restored LC3-II levels in cells depleted of spermidine, inhibition of eIF5A hypusination with *siDhs* or GC7 abrogated this effect (Figures 3F and S4I). Taken together the data indicate that spermidine maintains cellular basal autophagy by hypusinating eIF5A.

In activated T cells, eIF5A is one of the 20 most abundant proteins (Hukelmann et al., 2016). However, little is known about its role in lymphocytes. We measured eIF5A levels and autophagy in primary B cells during activation. LC3-II levels increased substantially over time upon activation in B cells, as measured using flow cytometry (Figure 3G) and western blot (Figures 3H and S4J). This time-dependent increase correlated with increasing levels of both total and hypusinated eIF5A (Figure 3H). In line with our data in NIH 3T3 cells, LC3-II levels decreased with increasing doses of GC7 (Figure 3I). This pathway operates independently of mTOR, whose activation inhibits autophagy. Indeed, GC7 did not activate mTOR, as demonstrated by S6 phosphorylation, a downstream substrate of mTOR (Figure S4K). Overall the data indicate that the eIF5A-autophagy pathway is induced upon activation of primary B cells, and physiological levels of hypusinated eIF5A are required for efficient autophagy.

eIF5A Hypusination Is Required for TFEB Expression

We next addressed how eIF5A regulates autophagy by identifying changes in expression of proteins involved in the autophagy pathway upon inhibition of eIF5A hypusination. We performed label-free quantitative mass spectrometry (MS) on nuclear and cytoplasmic fractions of activated primary B cells treated with GC7 (Figures 4A and S5A). This was followed by stable isotope labeling with amino acids in cell culture (SILAC) on activated primary B cells (Figures 4B and S5B). It has recently been reported that eIF5A regulates ATG3 protein synthesis in cell lines (Lubas et al., 2018). However, ATG3 protein levels were not found reduced in primary B cells treated with GC7, as shown in our MS results (Figure 4A) and confirmed by western blot (Figure S5C). Of the autophagy-related proteins detected, nuclear TFEB was the only one repeatedly found decreased upon GC7 treatment in both approaches. TFEB is a key transcription factor regulating autophagosomal and lysosomal biogenesis (Sardiello et al., 2009; Settembre et al., 2011). The effect of inhibition of eIF5A hypusination by GC7 on overall and nuclear TFEB was confirmed by western blot in activated primary B cells (Figures 4C and 4D). Accordingly, gene expression of several TFEB targets was markedly decreased upon GC7 treatment. Genetic KD of *Tfeb* was sufficient to reduce autophagic flux in NIH 3T3 cells (Figures 4F and S5D), as reported (Settembre et al., 2011). KD of *Eif5a* and *Dhs*, or GC7 treatment in NIH 3T3 cells, led to a decrease in TFEB protein (Figures 4G and 4H). To test if TFEB is affected by spermidine depletion, we inhibited spermidine synthesis in NIH 3T3 cells by *siOdc* or DFMO. This caused a reduction in TFEB, which was rescued by exogenous spermidine administration (Figures 4I and S5E), suggesting that spermidine controls cellular TFEB levels. However, spermidine failed to maintain TFEB levels when *Eif5a* was knocked down or eIF5A hypusination was inhibited (Figures 4J, S5F, and S5G), indicating that the regulation of TFEB by spermidine is mediated via eIF5A hypusination.

Being a paralog of TFEB, TFE3 has also been reported to promote autophagy and lysosomal biogenesis (Martina et al., 2014). Although TFE3 was not detected in either MS experiments (Figures 4A and 4B), western blots show that TFE3 was also reduced by GC7 treatment in B cells (Figure S5H). However, knocking down *Tfe3* alone did not significantly compromise autophagy or further reduce autophagy when knocked down together with *siTfeb* in NIH 3T3 cells (Figure S5I). Thus we focused on TFEB hereafter.

Hypusinated eIF5A Regulates TFEB Synthesis

TFEB is a very short-lived protein (<4 h half-life) (Xiao et al., 2015) compared with other proteins in the cell (median 36 h half-life) (Cambridge et al., 2011) and is therefore expected to require more active translation than most proteins. We first investigated if eIF5A controls overall protein synthesis in lymphocytes, as previously reported (Landau et al., 2010). We measured translation by flow cytometry with O-propargyl-puromycin (OPP) and found that inhibition of eIF5A hypusination by GC7 caused a 30% reduction in protein synthesis rate in activated primary B cells (Figure 5A). A similar reduction was observed using a puromycin-independent assay with L-azidohomoalanine (AHA; a methionine analog) incorporation (Figure S5J). To further assess translational changes caused by GC7, we performed ribosome profiling in activated primary B cells. Increased ribosome occupancy at the start codon was observed by GC7 treatment (Figure S5K). However, whether the accumulation of reads at the start codon is an artifact of cycloheximide treatment during sample preparation will require further validation. Stalling of ribosomes at the triproline-encoding motif (PPP) was found in activated primary B cells (Figure S5L), as observed in yeast and bacteria (Doerfel et al., 2013; Schuller et al., 2017; Ude et al., 2013; Woolstenhulme et al., 2015), indicating that polyproline is a conserved ribosome-pausing motif across kingdoms.

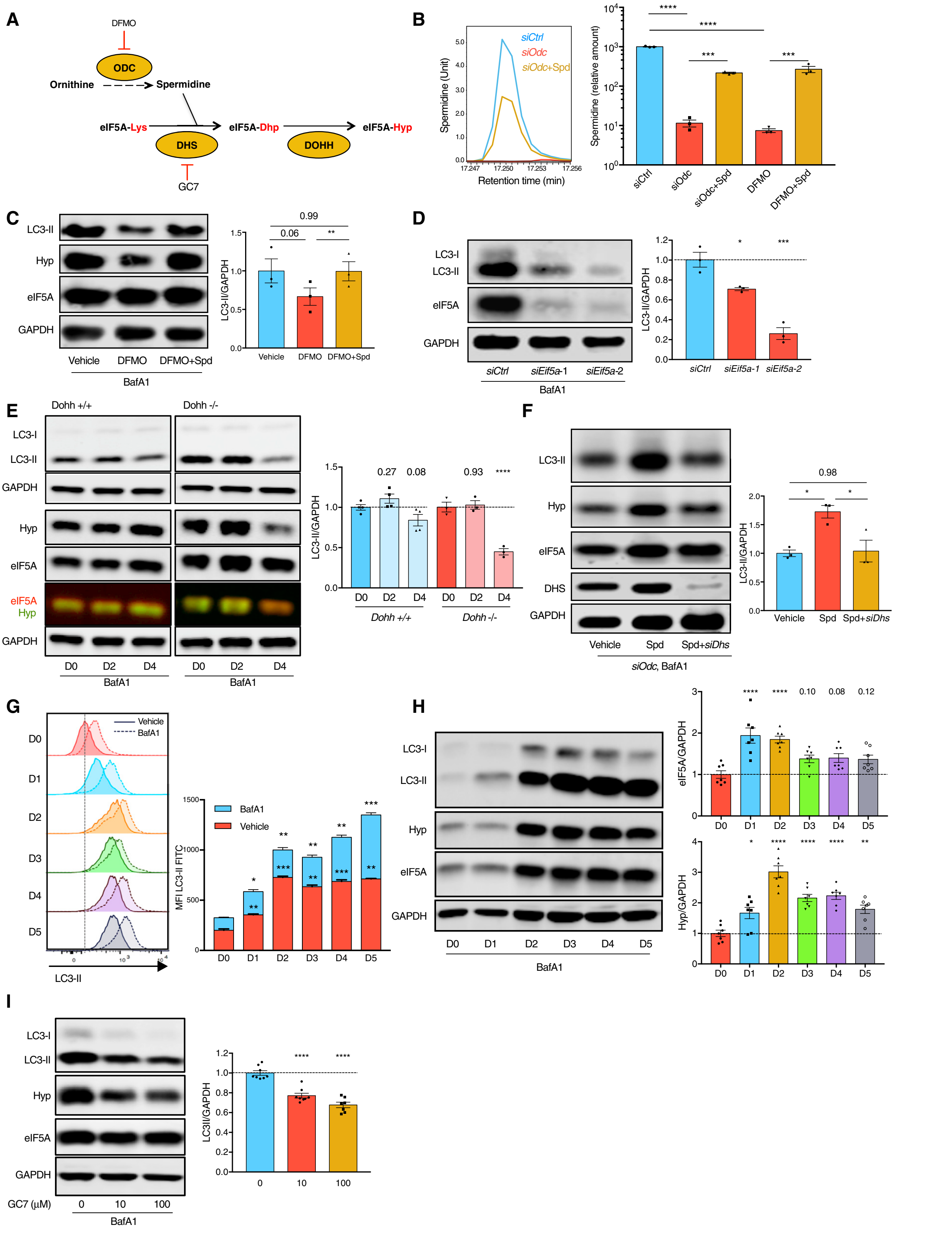

(legend on next page)

However, GC7 treatment did not further increase the ribosome occupancy at PPP motifs (Figure S5M). One explanation is that the no-go decay mechanism of mRNA at the severely stalled regions may lead to loss of reads (Doma and Parker, 2006).

We next investigated whether TFEB synthesis was affected by GC7 treatment by detecting nascent (recently made) proteins. By pulling down nascent proteins after 4 h of AHA labeling, a significant reduction of nascent TFEB was observed (Figures 5B and S5N). Interestingly, nascent eIF5A was also mildly reduced, suggesting an auto-regulatory loop of eIF5A at the translational level (Figures 5B and S5N). This is not reflected in overall eIF5A levels, probably because of the short treatment and the long half-life of eIF5A. Both human and mouse TFEB have at least one triproline motif (Figure 5C). Indeed, sequences around this triproline are also ribosome-pausing motifs (SPP and PPV) (Schuller et al., 2017), suggesting that this triproline-containing motif may confer TFEB with the specific requirement to rely on eIF5A for smooth translation. Therefore, we examined if the triproline motif in murine and human TFEB is sufficient to affect translation rate as regulated by hypusinated eIF5A. We generated three different constructs, using labile mCherry expression to report on the synthesis of either the TFEB triproline-containing motif, 13 consecutive prolines (13Pro), or a random sequence (Figure 5C). After transfection into NIH 3T3 cells, we measured translation using the ratio of the reporter mCherry to the transfection control GFP. As expected, the mCherry/GFP ratio was mildly reduced by GC7 for the random sequence and reduced by nearly half for the 13Pro motif, while the synthesis of TFEB triproline motif was also significantly inhibited (Figure 5D), indicating that the TFEB triproline motif requires hypusinated eIF5A for efficient synthesis. Moreover, mutating the triproline motif (PPP to AAA) partially rescued the expression of TFEB in the absence of hypusinated eIF5A (Figure 5E), indicating that the triproline motif is one, but not the only mechanism accounting for GC7-induced TFEB reduction. Overexpression of the triproline-mutated TFEB induced autophagy to a similar extent as wild-type TFEB (Figure 5F). However, when the nuclear localization sequence (NLS) was mutated in addition to triprolines (Roczniak-Ferguson et al., 2012), autophagy induction was lost. Taken together this indicates that the polyproline mutation does not compromise the function of TFEB as a transcription factor. Moreover, overexpressing mutant TFEB was sufficient to rescue autophagy in GC7-treated cells (Figure 5G), indicating that TFEB is critical for eIF5A-regulated autophagy. These data suggest that hypusinated eIF5A directly facilitates the synthesis of TFEB partially via the triproline-containing motif. Future studies are required to reveal other mechanisms of eIF5A-regulated TFEB expression.

Hypusination of eIF5A Is Essential for Hematopoiesis and B Cell Activation

eIF5A and its hypusination are essential for cellular growth in cultured cells (Park et al., 1994) and early embryonic development in mice (Nishimura et al., 2012), but its tissue-specific function is still unclear, although inducible whole-body KO of *Dhs* in mice leads to a fatal wasting syndrome (Pällmann et al., 2015). To investigate the function of hypusinated eIF5A during B cell development and activation *in vivo*, we generated competitive mixed bone marrow chimeras with inducible deletion of the hypusinating enzyme DHS. *Dhs* deletion was induced after long-term engraftment and B cell lineage reconstitution of CD45.2$^+$ cells was examined on days 8 and 30 after deletion (Figure S6A). Percentages of CD45.2$^+$ of both transitional and mature circulating B cells in peripheral blood were significantly affected by *Dhs* deletion on day 30 (Figures S6B and S6C). Upon sacrifice of the mice on day 34, we investigated if this was due to a loss of progenitors in the bone marrow. Unexpectedly, all multipotent hematopoietic progenitors investigated (HSC, MPP, LMPP, and LSK) were severely affected by DHS depletion (Figure S6D). This is in line with a bone marrow hypocellularity reported previously in whole-body *Dhs*-KO mice (Pällmann et al., 2015). Consistent with depleted HSCs, splenic CD45.2$^+$ myeloid cells, which mostly lack self-renewal capacity and rely on replenishment from progenitor cells, were also severely depleted after *Dhs* deletion (Figure S6E). Similarly, early B cell progenitors (pre-pro B cells), newly formed B cells and mature B cells from bone marrow as well as splenic transitional, marginal zone and follicular B cells were found significantly diminished (Figures S6F–S6H). The reduced B cell progenitors may result from either loss of HSCs or intrinsic survival defects. We checked if the remaining CD45.2$^+$ cells were indeed deleted for *Dhs* in blood (Figure S6I), bone marrow, and spleen (Figure S6J). This analysis revealed that although on day 8 after deletion with tamoxifen, blood cells were adequately deleted in the floxed *Dhs* mice, in cells that survived to day 34, *Dhs* was not deleted in most

Figure 3. Spermidine Maintains Cellular Autophagy by Hypusinating eIF5A.

(A) Spermidine synthesis and eIF5A hypusination pathway in eukaryotes.

(B) NIH 3T3 cells were transfected with non-targeting control siRNA (*siCtrl*), *siOdc* with or without 10 μM spermidine for 3 days, or treated with DFMO for 24 h where indicated. Cellular spermidine levels were measured using GC-MS. n = 3.

(C) NIH 3T3 cells were treated with DFMO and spermidine as indicated for 24 h. n = 3.

(D) NIH 3T3 cells were transfected with *siCtrl* or *siEif5a*-1/2 for 3 days. n = 3. *siEif5a-2* was used in all other figures unless specified otherwise.

(E) The KO of *Dohh* was induced by 4-OHT for indicated days in immortalized transgenic MEFs. n = 3 or 4.

(F) Spermidine-depleted NIH 3T3 cells by *siOdc* transfection were rescued with spermidine alone or spermidine together with *siDhs*. LC3-II was measured 3 days post-transfection. n = 3.

(G and H) Purified murine B cells were cultured with LPS for indicated days. LC3-II and/or eIF5A expression was measured using flow cytometry (G, n = 3 mice) and western blot (H, n = 7 mice).

(I) B cells were cultured as in (G) for 3 days with indicated concentrations of GC7 added on day 2 for 24 h. n = 6–10 mice.

To measure autophagic flux, cells were treated with 10 nM BafA1 for 2 h before harvesting where indicated. Data represented as mean ± SEM. One-way or two-way ANOVA with post hoc Dunnett's test (D, E, and G, where LC3-II levels under either basal [red bars] or BafA1 [red + blue bars] conditions are compared, H and I). One-way ANOVA with post hoc Tukey's test (B, C, and F). *p ≤ 0.05, **p ≤ 0.01, ***p ≤ 0.001, and ****p ≤ 0.0001.

See also Figures S3 and S4.

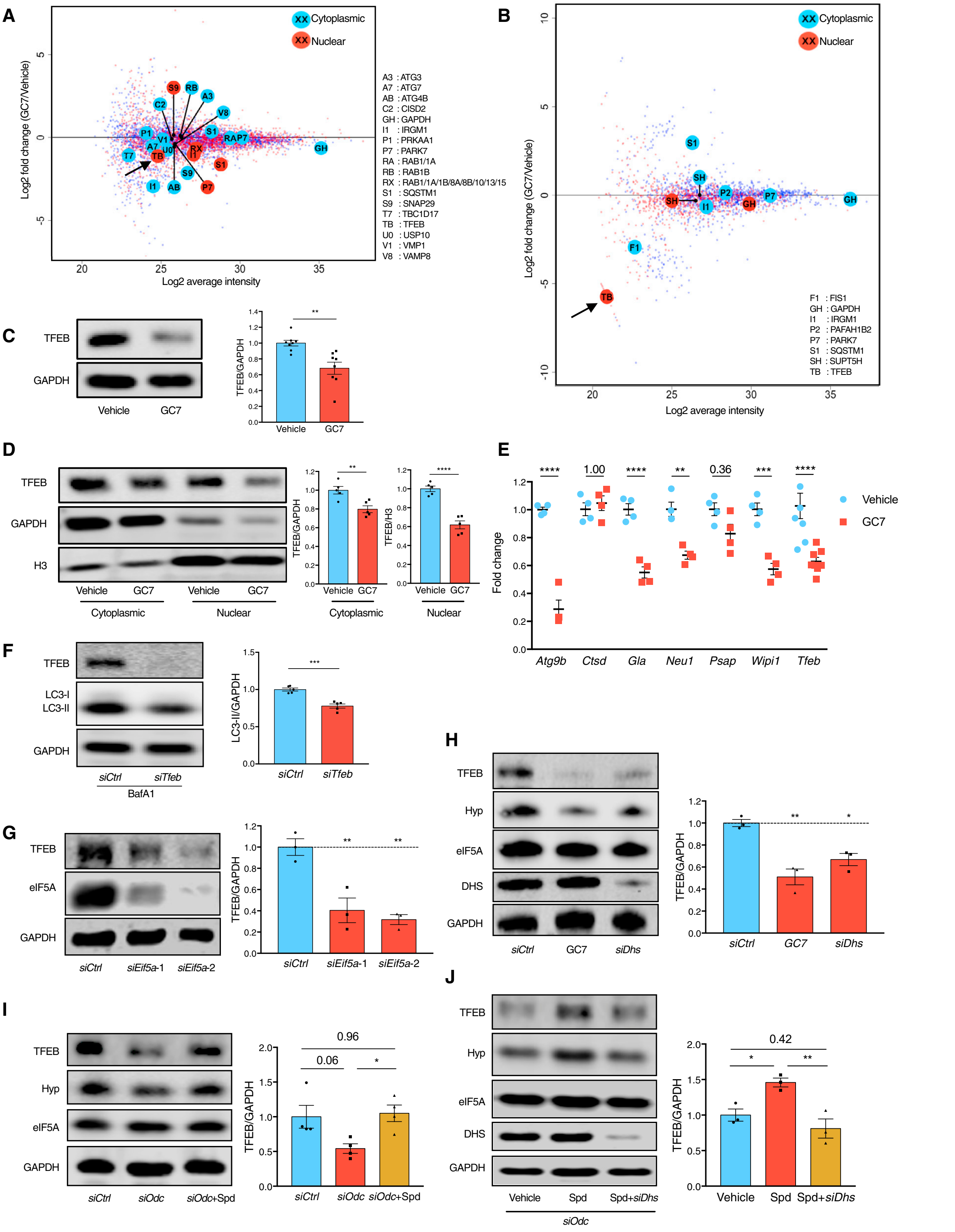

(legend on next page)

mice (Figure S6J). The overall cellularity in bone marrow and spleen was not affected despite the reduced CD45.2$^+$ cell numbers (Figure S6K), indicating that the remaining CD45.1$^+$ and non-deleted CD45.2$^+$ cells expanded and filled up the niche. These data together demonstrate a profound requirement of the eIF5A pathway in hematopoiesis and long-term survival of mature B cells.

To circumvent the defects of hematopoiesis and assess the role of eIF5A hypusination in B cell activation, we further attempted to delete *Dhs* after the first immunization with NP-CGG in *Dhs*-KO bone marrow chimeric mice supported with *Rag1*$^{-/-}$ bone marrow cells (to provide wild-type hematopoietic cells for the survival of the mice, without T and B cells). As before, after 3 weeks of deletion, all remaining lymphocytes expressed wild-type *Dhs* only. Levels of NP-specific IgG1 post-boost were not significantly changed in KO mice (data not shown), which can be attributed to contamination from non-deleted memory B cells. Overall the data imply that complete inhibition of eIF5A hypusination kills hematopoietic cells, and its effect on generation of antibodies cannot easily be investigated *in vivo*, because of (1) the rapid elimination of cells knocked out for *Dhs* and (2) the limiting deletion efficiency in mature B cells with inducible deletion models.

To investigate if eIF5A has an effect on B cell activation, we induced B cell activation *ex vivo* with LPS while inhibiting hypusination with GC7. The upregulation of early activation markers, including CD86, CD69, and MHC-II (Figure S6L) was not changed upon GC7 treatment. However, activation-induced cell proliferation was severely impaired upon inhibition of hypusinated eIF5A (Figure S6M) in a time-dependent fashion, in line with the known function of eIF5A in supporting cellular growth (Park et al., 1994). Taken together, these results indicate that hypusinated eIF5A is required for signaling events leading to specific cell fates such as proliferation and persistent antibody production for which autophagy might be required via its role in maintenance of organelle homeostasis (Pengo et al., 2013).

Spermidine Induces the Expression of Hypusinated eIF5A and TFEB in Old Mice

To assess the role of TFEB in murine B cells, we induced the KO of *Tfeb* in cultured murine B cells *ex vivo* (Figures 6A and S6N). Consistent with the reported functions of TFEB in inducing autophagy in other cell types (Figures 4F, 5F, S5D, and S5I; Settembre et al., 2011), deletion of *Tfeb* led to reduced autophagic flux in B cells (Figures 6A and 6B) without reducing cell viability (Figure S6O). Also, the expression of activation markers CD86 and MHC II was not compromised (Figures 6C and S6P). Functionally, antibody production was significantly reduced by *Tfeb* KO as assessed by secreted IgM in culture supernatants (Figure 6D). Therefore TFEB regulates antibody production of B cells, but the exact mechanisms require further investigation.

Spermidine levels have been reported to reduce with age in multiple organs, including mouse spleens (Nishimura et al., 2006). To assess whether the identified eIF5A-TFEB pathway is also affected during physiological aging and whether these changes can be rescued by exogenous spermidine, 2-year-old mice were treated with spermidine for 6 weeks. Interestingly, not only eIF5A hypusination but also overall eIF5A was significantly reduced in B cells from old mice (Figure 6E). The diminished overall eIF5A may partially result from a chronic decline in hypusination and eIF5A protein synthesis. Notably, a striking reduction of overall and especially nuclear TFEB was observed (Figures 6E and 6F), which might be a key cause of the reduced autophagic flux shown earlier (Figures 2A–2C). Importantly, spermidine supplementation restored the hypusination of eIF5A, overall eIF5A expression, and both overall and nuclear TFEB levels (Figures 6E and 6F). Thus, we observed an age-related decline of the spermidine-eIF5A-TFEB-autophagy pathway in B cells, which can be improved by spermidine supplementation. The fact that TFEB expression was only partially rescued by spermidine (Figures 6E and 6F) suggests the existence of alternative mechanisms regulating reduced TFEB expression during aging, which requires future investigation.

Spermidine Induces TFEB Expression and Improves the Function of Old Human B Cells

To investigate if this pathway regulates aging in human B cells, we first measured the levels of hypusinated eIF5A and TFEB in peripheral blood mononuclear cells (PBMCs) from healthy donors of different ages. Consistent with our findings in mice, PBMCs from donors aged 65 years and older showed a distinct reduction of TFEB protein. In some aged donors, TFEB was even undetectable by western blot (Figure 7A). In contrast, the mRNA of *TFEB* was not reduced in PBMCs from old donors (Figure 7B), further indicating that the diminished TFEB protein expression in the elderly is due to post-transcriptional changes. The overall eIF5A was significantly diminished in human PBMCs from

Figure 4. eIF5A Hypusination Is Required for TFEB Expression

(A) Murine B cells were treated with 10 μM GC7 as in Figure 3I and fractionated for label-free quantitative protein MS analysis. Identified autophagy proteins are highlighted by enlarged, annotated circles.

(B) Murine B cells were cultured in medium containing amino acids with heavy isotope labeling (GC7 treated) or light isotopes (vehicle). Cells that had divided four times or more were sorted by flow cytometry and mixed at 1:1 ratio (heavy: light isotope labeling), followed by cell fractionation and protein MS analysis. For the repeat, the labeling was swapped. Data represent the average of protein changes from the two repeats. Identified autophagy proteins are highlighted as in (A).

(C–E) Murine B cells were treated with GC7 as in (A). The overall TFEB (C) and cytoplasmic/nuclear TFEB (D) were assessed using western blot. (E) The expression of TFEB-target genes was measured using qPCR with *Gapdh* as the reference gene. n = 4–8 mice.

(F–I) NIH 3T3 cells were transfected with *siTfeb* (F), *siEif5a* (G), *siDhs* (H), or *siOdc* (I) and treated with 10 μM spermidine where indicated (I) for 3 days or treated with 100 μM GC7 for 24 h (H). LC3-II (F) or TFEB (G–I) was measured using western blot. n = 3–5. (J) NIH 3T3 cells were transfected with *siOdc* to deplete endogenous spermidine and treated with 10 μM spermidine alone or in combination with *siDhs* transfection. n = 3.

Data are represented as mean ± SEM. Student's t test (C, D, and F). Two-way ANOVA with post hoc Sidak's test (E). One-way ANOVA with post hoc Dunnett's test (G and H) or Tukey's test (I and J). $^*p \leq 0.05$, $^{**}p \leq 0.01$, $^{***}p \leq 0.001$, and $^{****}p \leq 0.0001$.

See also Figure S5.

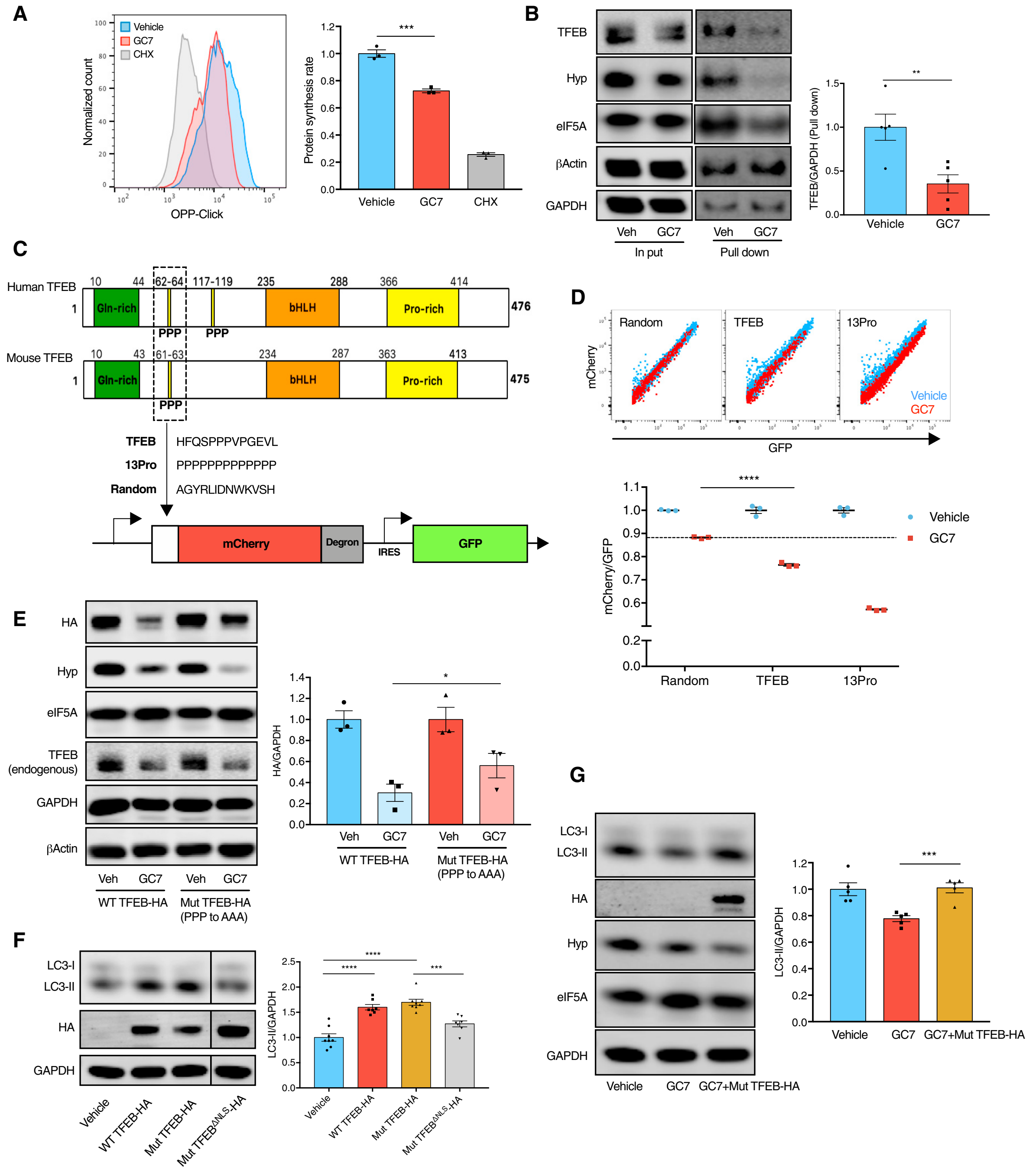

Figure 5. Hypusinated eIF5A Regulates TFEB Synthesis

(A) LPS-stimulated murine splenocytes were treated with GC7 as in Figure 3I or with cycloheximide (CHX) for 2 h. The relative protein synthesis rate of B cells ($B220^{+}CD19^{+}$) was measured using OPP-Click assay with flow cytometry. n = 3.

(B) Nascent proteins of GC7-treated murine B cells were labeled with AHA for 4 h, conjugated to biotin by click reaction, and pulled down for western blot. n = 5 mice.

(C) The polyproline motif of TFEB with its surrounding sequence was inserted before mCherry-degron to report on protein translation. GFP was used to report on transfection. Thirteen consecutive prolines (13Pro) was used as positive control for translational stalling, while 13 random amino acids (random) was used as negative control.

(D) NIH 3T3 cells were transfected with the plasmids in (C) for 24 h and treated together with GC7. The expression of GFP and mCherry was measured using flow cytometry. n = 3.

(legend continued on next page)

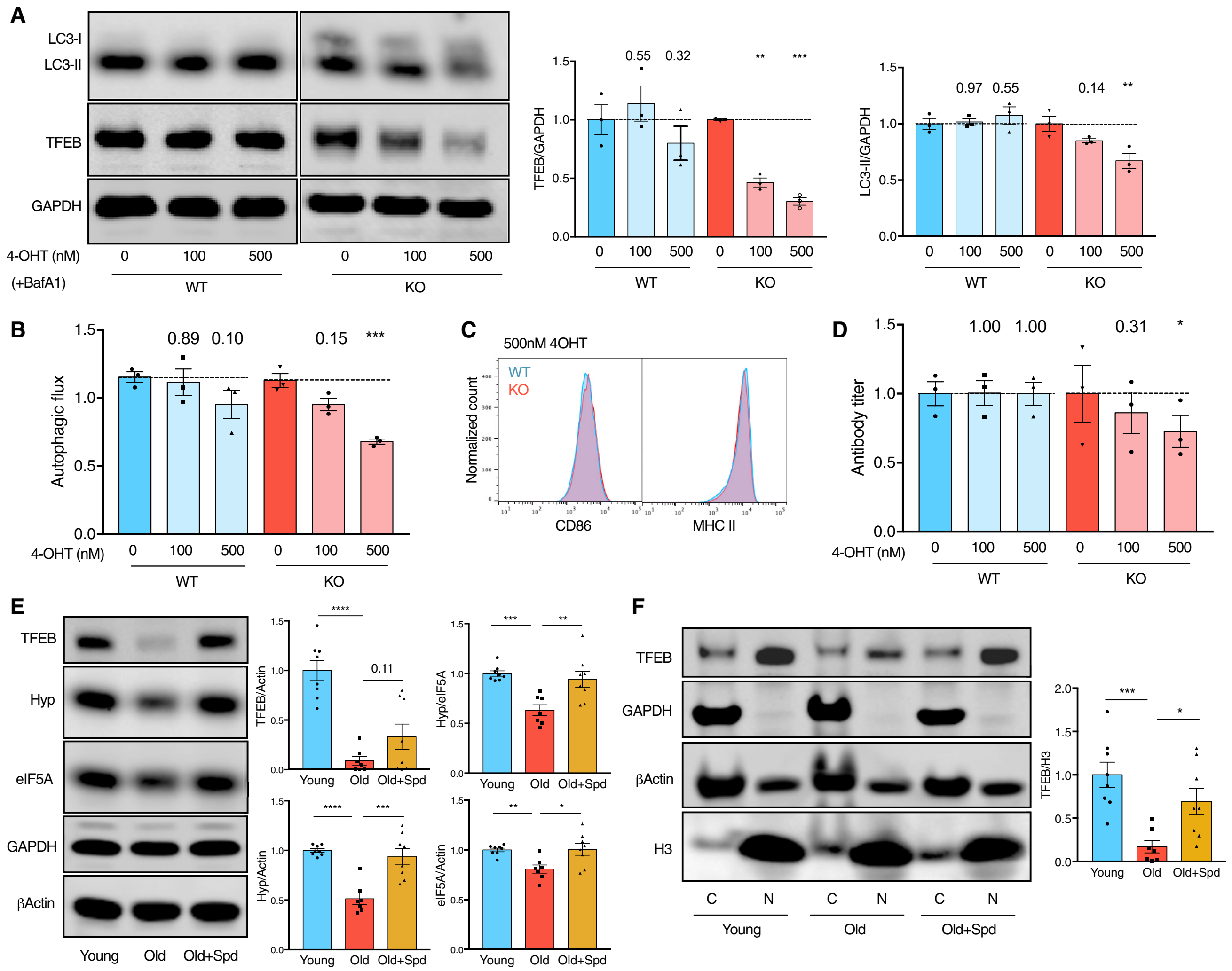

Figure 6. Spermidine Induces the Expression of Hypusinated eIF5A and TFEB in Old Mice

(A–D) Splenic B cells from tamoxifen-inducible *Tfeb*-KO mice (WT: *CAG-Cre/Esr1*$^{+}$, *Tfeb*$^{+/+}$; KO: *CAG-Cre/Esr1*$^{+}$, *Tfeb*$^{f/f}$) were cultured with IL4/anti-CD40 and 4-OHT for 4 days. The expression of TFEB, LC3-II, and the activation markers CD86/MHC II were assessed using western blot (A) and flow cytometry (B and C). The autophagic flux was calculated as in Figure 1A. IgM in culture supernatants was measured using ELISA (D). n = 3 mice.

(E and F) B cells were purified from young mice (6 weeks), old mice (24 months), or old mice administered spermidine for 6 weeks. The expression of overall eIF5A, hypusinated eIF5A, overall TFEB (E), cytoplasmic (C) and nuclear (N) TFEB (F) was assessed using western blot (n = 7 or 8 mice, combined from two independent experiments).

Data are represented as mean ± SEM. Two-way ANOVA with post hoc Dunnett's test (A, B, and D). Welch's t test (E and F). *p ≤ 0.05, **p ≤ 0.01, ***p ≤ 0.001, and ****p ≤ 0.0001.

See also Figure S6.

donors ≥65 years of age, but the remaining eIF5A was well hypusinated (Figure 7A), suggesting that cells may coordinate the expression of overall eIF5A with their hypusination levels during human aging. Indeed, TFEB protein levels correlate well with the expression of eIF5A (Figure S7A). Using mass spectrometry, we show that endogenous spermidine in human PBMCs declines with age (Figure S7B), as reported (Pucciarelli et al., 2012). Next, we investigated whether TFEB is regulated by spermidine and eIF5A in human primary B cells. Human B cells from young donors stimulated with anti-IgM and CD40L and treated with

(E–G) NIH 3T3 cells were transfected with the plasmids expressing wild-type (WT TFEB-HA) (E and F), mutant (Mut TFEB-HA, PPP to AAA) (E–G), or NLS-mutated (ΔNLS, RRRR to AAAA) mutant (F) murine TFEB fused with an HA tag at C terminus for 24 h, treated together with GC7 (E and G). The expression of HA-tagged TFEB (E) and LC3-II (F and G) was assessed using western blot. Irrelevant lanes from the same membrane were removed (F). n = 3–8.

Data are represented as mean ± SEM. Student's t test (A, B, D, and G), paired t test (E), or one-way ANOVA with post hoc Tukey's test (F). *p ≤ 0.05, **p ≤ 0.01, ***p ≤ 0.001, and ****p ≤ 0.0001.

See also Figure S5.

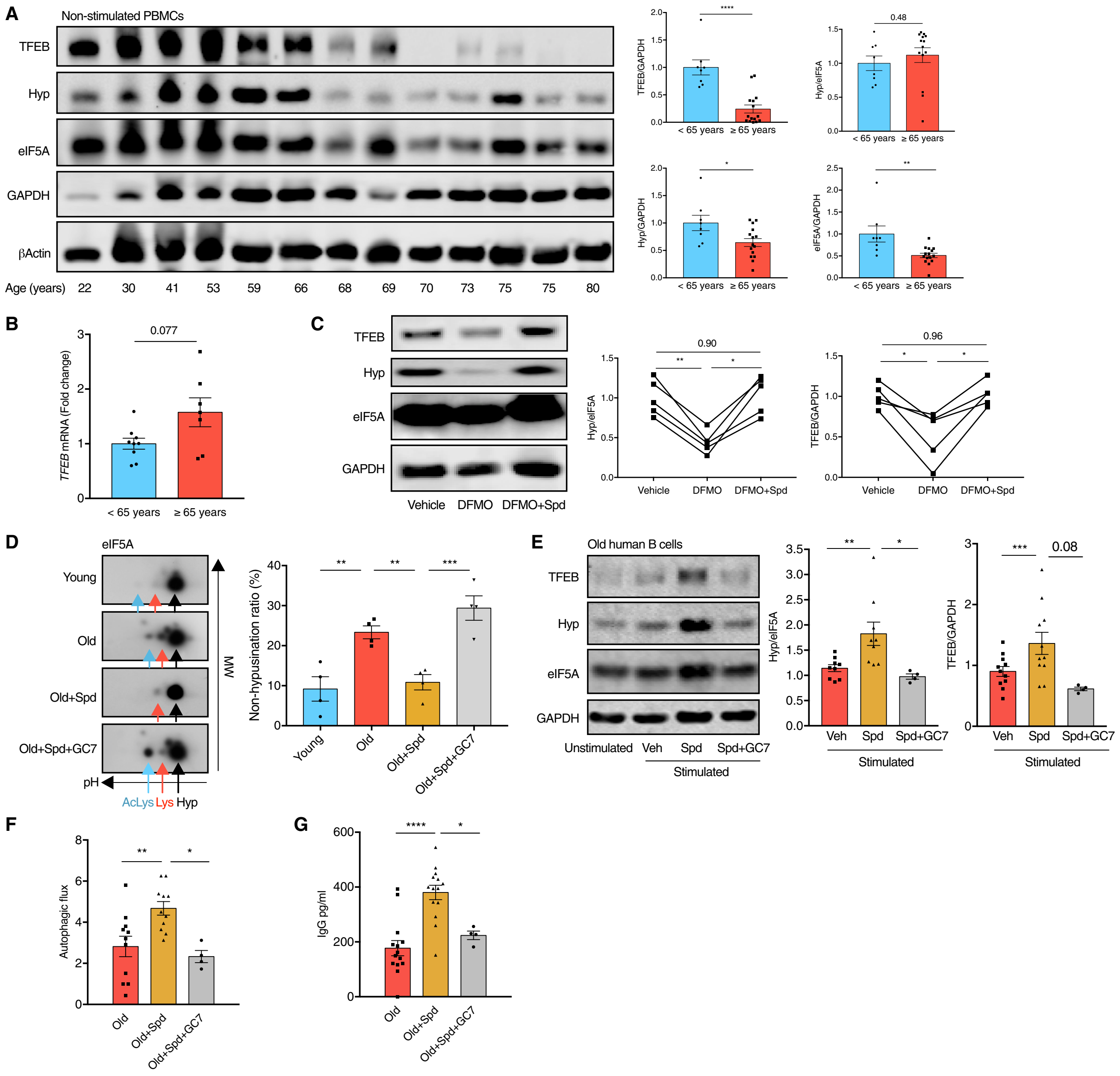

Figure 7. Spermidine Induces TFEB Expression and Improves the Function of Old Human B Cells

(A) The protein levels of TFEB, hypusinated eIF5A, and overall eIF5A of PBMCs from healthy human donors of indicated ages were assessed using western blot. A representative plot (left) and quantifications (right) are shown. n = 8–15 donors.

(B) *TFEB* mRNA of human PBMCs was measured using qPCR with *GAPDH* as the reference gene. n = 7–9 donors.

(C) Sorted B cells from young human donors were cultured with anti-IgM/CD40L and treated with DFMO with or without 10 μM spermidine for 7 days. n = 5 donors.

(D) Sorted B cells from human donors were cultured as in (C) with spermidine and/or GC7 as indicated. The expression of hypusinated (Hyp) or non-hypusinated (AcLys or Lys) eIF5A was distinguished by two-dimensional western blot of eIF5A. Black arrow, hypusinated Lys^{50}, pH 5.2; red arrow, unmodified Lys^{50}, pH 5.1; blue arrow, acetylated Lys^{47} with unmodified Lys^{50}, pH 5.0. The non-hypusination ratio was calculated as eIF5A dot densitometric intensity (AcLys + Lys)/(AcLys + Lys + Hyp). n = 4 donors.

(E–G) Sorted B cells from old human donors (age 77.5 ± 6.3 years) were cultured as in (C) together with spermidine and GC7. (E) The protein levels of TFEB and eIF5A hypusination were measured using western blot. (F) Autophagic flux was determined by flow cytometry as in Figure 1A. (G) Supernatant IgG was assessed using ELISA. n = 4–14 donors.

(legend continued on next page)

DFMO *ex vivo* showed reduced eIF5A hypusination and TFEB, which were rescued by exogenous spermidine supplementation (Figure 7C).

Last and most important, we tested if spermidine improves old human B cell responses. In contrast to non-stimulated naive PBMCs, *ex vivo* activated old human B cells showed accumulated non-hypusinated eIF5A, suggesting a defect in hypusination (Figure 7D). The levels of both eIF5A hypusination and TFEB (Figures 7D and 7E), as well as autophagic flux (Figure 7F) were significantly restored by spermidine treatment in B cells from aged donors in which spermidine levels are naturally low. IgG production by B cells from aged donors was also improved (Figure 7G). GC7 treatment abrogated these effects of spermidine (Figures 7D–7G), suggesting that spermidine improves autophagy and antibody production of old B cells via hypusinating eIF5A. In contrast, neither the eIF5A-TFEB-autophagy pathway nor IgG production was increased by spermidine in young human B cells (Figures S7C–S7E). Thus, replenishment of spermidine in B cells from aged donors rejuvenated their function via eIF5A hypusination.

DISCUSSION

This study demonstrates that autophagic flux is regulated translationally. Although acute autophagy induction may not require gene expression and protein synthesis, with components being readily available, long-term autophagic flux does depend on continued gene expression (Lawrence and Brown, 1993). We identified one specific mechanism of this regulation, whereby the polyamine metabolite spermidine is a substrate for the hypusination of the translation factor eIF5A, which in turn controls the synthesis of TFEB protein. This mechanism operates normally in the young, in whom spermidine is abundant. In B cells from old individuals, however, replenishing spermidine levels restored this pathway, thereby boosting responses *in vivo* in mice and *ex vivo* in humans. Depleted spermidine levels and subsequent low TFEB expression may be an important cause of the reduction in autophagy in the aging adaptive immune system, as well as in other tissues. However, other potential direct or indirect mechanisms may play a role in the anti-aging effects of polyamines, such as epigenetic modifications or changes in metabolism (Puleston et al., 2019).

It was reported that GC7 induces autophagy in the human fibrosarcoma 2fTGH cell line (Oliverio et al., 2014). The discrepancy between this and our findings may be a result of the high dose of GC7 (200 μM) used as opposed to 10 μM for primary B cells and 100 μM for NIH 3T3 cells used here. Treatment with a high drug dose may induce stress responses and autophagy non-specifically, as demonstrated here for spermidine (Figures S3H–S3M). In line, the study by Oliverio et al. (2014) shows that genetic ablation of eIF5A or of its hypusination enzymes consistently tends to reduce (not induce) LC3-II expression in 2fTGH cells.

Coordination of Translation and Autophagy

Translation in lymphocytes is little studied in T cells (Hukelmann et al., 2016; Tan et al., 2017) and B cells (Diaz-Muñoz et al., 2015). Naive B cells are in a metabolically quiescent state, with low protein synthesis (low eIF5A expression) and autophagy, both of which are quickly upregulated after activation. Autophagy and the translation machinery may then form a regulatory loop to support the metabolic requirements of proliferation and immunoglobulin production. In this loop, autophagy provides substrates and energy for increased translation, while inducing the synthesis of certain autophagic proteins such as TFEB. Specifically, a particular requirement for rapid provision of amino acids and unfolded protein degradation by autophagy may exist in long-lived plasma cells, which secrete antibodies at the rate of about 2,000 molecules per second (Clarke et al., 2015; Pengo et al., 2013).

Translational Control of TFEB

The anti-aging function of TFEB seems to be highly conserved across species. Earlier studies found it to be an important transcription factor for lifespan extension in *C. elegans* (Lapierre et al., 2013). Moreover, it is likely that the control of TFEB via translation is a broad, maybe universal mechanism, operating across many tissue types (here in NIH 3T3 cells and in mouse and human primary B cells). TFEB is known to be post-translationally controlled via (1) mTORC1-dependent phosphorylation for cytoplasmic retention, and (2) calcium-dependent dephosphorylation to signal for nuclear translocation (Napolitano and Ballabio, 2016). We now add an mTOR-independent step of regulation at the level of translation.

ATG3 protein synthesis was recently reported to be controlled by hypusinated eIF5A in cell lines (Lubas et al., 2018). However, we could not find a reduction of ATG3 with GC7 treatment in primary B cells, indicating that it may be cell type specific (Figures 4A and S5C). MHC II also contains polyproline motifs but did not reduce with GC7 treatment either (Figure S6L). These observations suggest that the presence of a polyproline motif alone is insufficient to cause reduced protein expression by inhibition of hypusinated eIF5A. Indeed, research in bacteria showed that polyproline-induced stalls may lead to reduced protein levels only if they limit translation more than initiation (Hersch et al., 2014). Moreover, an auto-regulatory loop exists for the regulation of *Tfeb* transcription (Settembre et al., 2013). Therefore, defective TFEB synthesis may initiate a vicious cycle involving reduced *Tfeb* transcription (Figure 4E) and further reduced protein synthesis, eventually leading to overall diminished TFEB protein. Additionally, we hypothesize that a stronger stalling motif (such as SPPPVP in TFEB), and TFEB's short half-life may contribute to reduced protein expression in the absence of hypusinated eIF5A, and predict that other such proteins are regulated by eIF5A.

Harness the Pathway to Rejuvenate Human Aging

We have shown eIF5A and TFEB levels are correlated with age in immune cells. Blood aging biomarkers are urgently needed to

Data are represented as mean ± SEM. Unpaired two-tailed Student's t test (A and D, comparison of young versus old). Welch's t test (B). Paired one-way ANOVA with post hoc Tukey's test (C). Paired one-tailed t test (D, comparisons of old versus old + Spd, old + Spd versus old + Spd + GC7 comparison, E–G). $^{*}p \leq 0.05$, $^{**}p \leq 0.01$, $^{***}p \leq 0.001$, and $^{****}p \leq 0.0001$.
See also Figure S7.

gauge the efficacy of new drugs that prolong health span. Autophagy is one of the few general mechanisms underpinning many age-related diseases and is therefore a good target for anti-aging drugs. Last, it has been accepted for decades that hypusination occurs during or shortly after the synthesis of eIF5A, and the majority of cellular eIF5A is in its hypusinated form unless artificial treatments are being used to block the hypusination pathway. Here we show that under certain physiological conditions, such as aging, a significant pool of non-hypusinated eIF5A accumulates, probably because of the lack of spermidine (Figures 6E, 7D, and 7E). This indicates that hypusination of eIF5A can potentially be harnessed therapeutically for aging or age-related diseases.

STAR★METHODS

Detailed methods are provided in the online version of this paper and include the following:

- KEY RESOURCES TABLE
- LEAD CONTACT AND MATERIALS AVAILABILITY
- EXPERIMENTAL MODEL AND SUBJECT DETAILS
  - Mice
  - Cell Lines
  - Human Peripheral Blood
- METHOD DETAILS
  - Bone Marrow Chimera
  - Mouse Immunization
  - Mouse B Cell Purification and Stimulation
  - Drug Treatments
  - One- and Two-Dimensional Western Blot
  - Immunoprecipitation
  - ELISA
  - ELISpot
  - Plasmid Construction
  - Transfection
  - Flow Cytometry
  - CytoID Staining
  - Quantitative PCR
  - Confocal Microscopy
  - Cell Fractionation
  - Protein Mass Spectrometry
  - Spermidine Measurement by Gas Chromatography Mass Spectrometry (GC-MS)
  - OPP-Click Assay
  - AHA Labeling of Nascent Proteins
  - Ribosome Profiling
  - Human B Cell Assays
- QUANTIFICATION AND STATISTICAL ANALYSIS
- DATA AND CODE AVAILABILITY

SUPPLEMENTAL INFORMATION

Supplemental Information can be found online at https://doi.org/10.1016/j.molcel.2019.08.005.

ACKNOWLEDGMENTS

We thank the staff of the University of Oxford Biomedical Services Unit for animal care, Jonathan Webber and Craig Waugh for assistance with fluorescence-activated cell sorting (FACS), Volodymyr Nechyporuk-Zloy for assistance with confocal microscopy imaging, and Alfredo Castello Palomares for help with SILAC proteomics and providing general advice. Alexander Clarke is acknowledged for reading the manuscript. H.Z. is funded by the China Scholarship Council-Nuffield Department of Medicine Scholarship and the Oxford-Elysium Prize Fellowship, and the A.K.S. lab is funded by a Wellcome Trust Investigator Award (103830/Z/14/Z). S.B. is funded by a Swiss National Science Foundation grant (31003A_150066). J.F. is funded by the Wellcome Trust Chromosome and Developmental Biology PhD Program (ALR00520). The J.M. lab is funded by a Biotechnology and Biological Sciences Research Council (BBSRC) grant (BB/P00296X/1). A.B. is funded by the Italian Telethon Foundation (TGM16CB6). Li-Cor Odyssey imager is funded by the European Research Council (ERC) (AdG 670930).

AUTHOR CONTRIBUTIONS

H.Z. designed and performed most experiments. G.A. performed human B cell studies. J.F. helped set up and analyze ribosome profiling in primary B cells. P.C. and S.M. generated proteomic data. Y.S. and L.F. ran some western blots. S.B. provided KD/KO cell lines and *Dhs*-KO bone marrow and performed two-dimensional (2D) western blotting. G.N. and A.B. provided *Tfeb*-KO spleens. P.H. made the constructs of NLS-mutated *Tfeb*. T.R. set up the *Dhs*-KO chimera experiments. Z.Y. performed GC-MS. J.M. and S.B. gave advice on data interpretation and corrected the manuscript. A.K.S. supervised this project (H.Z.'s thesis), designed the experiments, provided funds, and wrote the manuscript.

DECLARATION OF INTERESTS

A.B. is a co-founder of CASMA Therapeutics.

Received: December 3, 2018
Revised: April 30, 2019
Accepted: August 2, 2019
Published: August 29, 2019

REFERENCES

Bjedov, I., Toivonen, J.M., Kerr, F., Slack, C., Jacobson, J., Foley, A., and Partridge, L. (2010). Mechanisms of life span extension by rapamycin in the fruit fly Drosophila melanogaster. Cell Metab. *11*, 35–46.

Cambridge, S.B., Gnad, F., Nguyen, C., Bermejo, J.L., Krüger, M., and Mann, M. (2011). Systems-wide proteomic analysis in mammalian cells reveals conserved, functional protein turnover. J. Proteome Res. *10*, 5275–5284.

Chen, M., Hong, M.J., Sun, H., Wang, L., Shi, X., Gilbert, B.E., Corry, D.B., Kheradmand, F., and Wang, J. (2014). Essential role for autophagy in the maintenance of immunological memory against influenza infection. Nat. Med. *20*, 503–510.

Clarke, A.J., Ellinghaus, U., Cortini, A., Stranks, A., Simon, A.K., Botto, M., and Vyse, T.J. (2015). Autophagy is activated in systemic lupus erythematosus and required for plasmablast development. Ann. Rheum. Dis. *74*, 912–920.

Cossarizza, A., Chang, H.D., Radbruch, A., Akdis, M., Andrä, I., Annunziato, F., Bacher, P., Barnaba, V., Battistini, L., Bauer, W.M., et al. (2017). Guidelines for the use of flow cytometry and cell sorting in immunological studies. Eur. J. Immunol. *47*, 1584–1797.

Cox, J., and Mann, M. (2008). MaxQuant enables high peptide identification rates, individualized p.p.b.-range mass accuracies and proteome-wide protein quantification. Nat. Biotechnol. *26*, 1367–1372.

Cox, J., Neuhauser, N., Michalski, A., Scheltema, R.A., Olsen, J.V., and Mann, M. (2011). Andromeda: a peptide search engine integrated into the MaxQuant environment. J. Proteome Res. *10*, 1794–1805.

Dever, T.E., Gutierrez, E., and Shin, B.S. (2014). The hypusine-containing translation factor eIF5A. Crit. Rev. Biochem. Mol. Biol. *49*, 413–425.

Diaz-Muñoz, M.D., Bell, S.E., Fairfax, K., Monzon-Casanova, E., Cunningham, A.F., Gonzalez-Porta, M., Andrews, S.R., Bunik, V.I., Zarnack, K., Curk, T.,

et al. (2015). The RNA-binding protein HuR is essential for the B cell antibody response. Nat. Immunol. *16*, 415–425.

Doerfel, L.K., Wohlgemuth, I., Kothe, C., Peske, F., Urlaub, H., and Rodnina, M.V. (2013). EF-P is essential for rapid synthesis of proteins containing consecutive proline residues. Science *339*, 85–88.

Doma, M.K., and Parker, R. (2006). Endonucleolytic cleavage of eukaryotic mRNAs with stalls in translation elongation. Nature *440*, 561–564.

Eisenberg, T., Knauer, H., Schauer, A., Büttner, S., Ruckenstuhl, C., Carmona-Gutierrez, D., Ring, J., Schroeder, S., Magnes, C., Antonacci, L., et al. (2009). Induction of autophagy by spermidine promotes longevity. Nat. Cell Biol. *11*, 1305–1314.

Eisenberg, T., Abdellatif, M., Schroeder, S., Primessnig, U., Stekovic, S., Pendl, T., Harger, A., Schipke, J., Zimmermann, A., Schmidt, A., et al. (2016). Cardioprotection and lifespan extension by the natural polyamine spermidine. Nat. Med. *22*, 1428–1438.

Fernández, A.F., Sebti, S., Wei, Y., Zou, Z., Shi, M., McMillan, K.L., He, C., Ting, T., Liu, Y., Chiang, W.C., et al. (2018). Disruption of the beclin 1-BCL2 autophagy regulatory complex promotes longevity in mice. Nature *558*, 136–140.

Henry, C.J., Marusyk, A., and DeGregori, J. (2011). Aging-associated changes in hematopoiesis and leukemogenesis: what's the connection? Aging (Albany N.Y.) *3*, 643–656.

Hersch, S.J., Elgamal, S., Katz, A., Ibba, M., and Navarre, W.W. (2014). Translation initiation rate determines the impact of ribosome stalling on bacterial protein synthesis. J. Biol. Chem. *289*, 28160–28171.

Hukelmann, J.L., Anderson, K.E., Sinclair, L.V., Grzes, K.M., Murillo, A.B., Hawkins, P.T., Stephens, L.R., Lamond, A.I., and Cantrell, D.A. (2016). The cytotoxic T cell proteome and its shaping by the kinase mTOR. Nat. Immunol. *17*, 104–112.

Ingolia, N.T. (2010). Genome-wide translational profiling by ribosome footprinting. Methods Enzymol. *470*, 119–142.

Ingolia, N.T., Brar, G.A., Rouskin, S., McGeachy, A.M., and Weissman, J.S. (2013). Genome-wide annotation and quantitation of translation by ribosome profiling. Curr. Protoc. Mol. Biol. *Chapter 4*. Unit 4.18.

Kelly, S.M., Vanslyke, J.K., and Musil, L.S. (2007). Regulation of ubiquitin-proteasome system mediated degradation by cytosolic stress. Mol. Biol. Cell *18*, 4279–4291.

Klinman, N.R., and Kline, G.H. (1997). The B-cell biology of aging. Immunol. Rev. *160*, 103–114.

Komatsu, M., Waguri, S., Ueno, T., Iwata, J., Murata, S., Tanida, I., Ezaki, J., Mizushima, N., Ohsumi, Y., Uchiyama, Y., et al. (2005). Impairment of starvation-induced and constitutive autophagy in Atg7-deficient mice. J. Cell Biol. *169*, 425–434.

Landau, G., Bercovich, Z., Park, M.H., and Kahana, C. (2010). The role of polyamines in supporting growth of mammalian cells is mediated through their requirement for translation initiation and elongation. J. Biol. Chem. *285*, 12474–12481.

Lapierre, L.R., De Magalhaes Filho, C.D., McQuary, P.R., Chu, C.C., Visvikis, O., Chang, J.T., Gelino, S., Ong, B., Davis, A.E., Irazoqui, J.E., et al. (2013). The TFEB orthologue HLH-30 regulates autophagy and modulates longevity in Caenorhabditis elegans. Nat. Commun. *4*, 2267.

Lawrence, B.P., and Brown, W.J. (1993). Inhibition of protein synthesis separates autophagic sequestration from the delivery of lysosomal enzymes. J. Cell Sci. *105*, 473–480.

Lee, I.H., and Finkel, T. (2009). Regulation of autophagy by the p300 acetyltransferase. J. Biol. Chem. *284*, 6322–6328.

Lubas, M., Harder, L.M., Kumsta, C., Tiessen, I., Hansen, M., Andersen, J.S., Lund, A.H., and Frankel, L.B. (2018). eIF5A is required for autophagy by mediating ATG3 translation. EMBO Rep. *19*, e46072.

Madeo, F., Eisenberg, T., Pietrocola, F., and Kroemer, G. (2018). Spermidine in health and disease. Science *359*, eaan2788.

Martina, J.A., Diab, H.I., Lishu, L., Jeong-A, L., Patange, S., Raben, N., and Puertollano, R. (2014). The nutrient-responsive transcription factor TFE3 promotes autophagy, lysosomal biogenesis, and clearance of cellular debris. Sci. Signal. *7*, ra9.

Mizushima, N., Yamamoto, A., Matsui, M., Yoshimori, T., and Ohsumi, Y. (2004). In vivo analysis of autophagy in response to nutrient starvation using transgenic mice expressing a fluorescent autophagosome marker. Mol. Biol. Cell *15*, 1101–1111.

Morselli, E., Mariño, G., Bennetzen, M.V., Eisenberg, T., Megalou, E., Schroeder, S., Cabrera, S., Bénit, P., Rustin, P., Criollo, A., et al. (2011). Spermidine and resveratrol induce autophagy by distinct pathways converging on the acetylproteome. J. Cell Biol. *192*, 615–629.

Napolitano, G., and Ballabio, A. (2016). TFEB at a glance. J. Cell Sci. *129*, 2475–2481.

Nishimura, K., Shiina, R., Kashiwagi, K., and Igarashi, K. (2006). Decrease in polyamines with aging and their ingestion from food and drink. J. Biochem. *139*, 81–90.

Nishimura, K., Lee, S.B., Park, J.H., and Park, M.H. (2012). Essential role of eIF5A-1 and deoxyhypusine synthase in mouse embryonic development. Amino Acids *42*, 703–710.

Oliverio, S., Corazzari, M., Sestito, C., Piredda, L., Ippolito, G., and Piacentini, M. (2014). The spermidine analogue GC7 (N1-guanyl-1,7-diamineoheptane) induces autophagy through a mechanism not involving the hypusination of eIF5A. Amino Acids *46*, 2767–2776.

Pällmann, N., Braig, M., Sievert, H., Preukschas, M., Hermans-Borgmeyer, I., Schweizer, M., Nagel, C.H., Neumann, M., Wild, P., Haralambieva, E., et al. (2015). Biological relevance and therapeutic potential of the hypusine modification system. J. Biol. Chem. *290*, 18343–18360.

Park, M.H., Wolff, E.C., Lee, Y.B., and Folk, J.E. (1994). Antiproliferative effects of inhibitors of deoxyhypusine synthase. Inhibition of growth of Chinese hamster ovary cells by guanyl diamines. J. Biol. Chem. *269*, 27827–27832.

Pengo, N., Scolari, M., Oliva, L., Milan, E., Mainoldi, F., Raimondi, A., Fagioli, C., Merlini, A., Mariani, E., Pasqualetto, E., et al. (2013). Plasma cells require autophagy for sustainable immunoglobulin production. Nat. Immunol. *14*, 298–305.

Phadwal, K., Alegre-Abarrategui, J., Watson, A.S., Pike, L., Anbalagan, S., Hammond, E.M., Wade-Martins, R., McMichael, A., Klenerman, P., and Simon, A.K. (2012). A novel method for autophagy detection in primary cells: impaired levels of macroautophagy in immunosenescent T cells. Autophagy *8*, 677–689.

Pietrocola, F., Lachkar, S., Enot, D.P., Niso-Santano, M., Bravo-San Pedro, J.M., Sica, V., Izzo, V., Maiuri, M.C., Madeo, F., Mariño, G., and Kroemer, G. (2015). Spermidine induces autophagy by inhibiting the acetyltransferase EP300. Cell Death Differ. *22*, 509–516.

Preukschas, M., Hagel, C., Schulte, A., Weber, K., Lamszus, K., Sievert, H., Pällmann, N., Bokemeyer, C., Hauber, J., Braig, M., and Balabanov, S. (2012). Expression of eukaryotic initiation factor 5A and hypusine forming enzymes in glioblastoma patient samples: implications for new targeted therapies. PLoS ONE *7*, e43468.

Pucciarelli, S., Moreschini, B., Micozzi, D., De Fronzo, G.S., Carpi, F.M., Polzonetti, V., Vincenzetti, S., Mignini, F., and Napolioni, V. (2012). Spermidine and spermine are enriched in whole blood of nona/centenarians. Rejuvenation Res. *15*, 590–595.

Puleston, D.J., Zhang, H., Powell, T.J., Lipina, E., Sims, S., Panse, I., Watson, A.S., Cerundolo, V., Townsend, A.R., Klenerman, P., and Simon, A.K. (2014). Autophagy is a critical regulator of memory CD8(+) T cell formation. eLife *3*, e03706.

Puleston, D.J., Buck, M.D., Klein Geltink, R.I., Kyle, R.L., Caputa, G., O'Sullivan, D., Cameron, A.M., Castoldi, A., Musa, Y., Kabat, A.M., et al. (2019). Polyamines and eIF5A hypusination modulate mitochondrial respiration and macrophage activation. Cell Metab. *30*, 352–363.e8.

Pyo, J.O., Yoo, S.M., Ahn, H.H., Nah, J., Hong, S.H., Kam, T.I., Jung, S., and Jung, Y.K. (2013). Overexpression of Atg5 in mice activates autophagy and extends lifespan. Nat. Commun. *4*, 2300.

Roczniak-Ferguson, A., Petit, C.S., Froehlich, F., Qian, S., Ky, J., Angarola, B., Walther, T.C., and Ferguson, S.M. (2012). The transcription factor TFEB links mTORC1 signaling to transcriptional control of lysosome homeostasis. Sci. Signal. *5*, ra42.

Rossi, D., Kuroshu, R., Zanelli, C.F., and Valentini, S.R. (2014). eIF5A and EF-P: two unique translation factors are now traveling the same road. Wiley Interdiscip. Rev. RNA *5*, 209–222.

Sardiello, M., Palmieri, M., di Ronza, A., Medina, D.L., Valenza, M., Gennarino, V.A., Di Malta, C., Donaudy, F., Embrione, V., Polishchuk, R.S., et al. (2009). A gene network regulating lysosomal biogenesis and function. Science *325*, 473–477.

Schuller, A.P., Wu, C.C., Dever, T.E., Buskirk, A.R., and Green, R. (2017). eIF5A functions globally in translation elongation and termination. Mol. Cell *66*, 194–205.e5.

Settembre, C., Di Malta, C., Polito, V.A., Garcia Arencibia, M., Vetrini, F., Erdin, S., Erdin, S.U., Huynh, T., Medina, D., Colella, P., et al. (2011). TFEB links autophagy to lysosomal biogenesis. Science *332*, 1429–1433.

Settembre, C., Zoncu, R., Medina, D.L., Vetrini, F., Erdin, S., Erdin, S., Huynh, T., Ferron, M., Karsenty, G., Vellard, M.C., et al. (2012). A lysosome-to-nucleus signalling mechanism senses and regulates the lysosome via mTOR and TFEB. EMBO J. *31*, 1095–1108.

Settembre, C., De Cegli, R., Mansueto, G., Saha, P.K., Vetrini, F., Visvikis, O., Huynh, T., Carissimo, A., Palmer, D., Klisch, T.J., et al. (2013). TFEB controls cellular lipid metabolism through a starvation-induced autoregulatory loop. Nat. Cell Biol. *15*, 647–658.

Sievert, H., Pällmann, N., Miller, K.K., Hermans-Borgmeyer, I., Venz, S., Sendoel, A., Preukschas, M., Schweizer, M., Boettcher, S., Janiesch, P.C., et al. (2014). A novel mouse model for inhibition of DOHH-mediated hypusine modification reveals a crucial function in embryonic development, proliferation and oncogenic transformation. Dis. Model. Mech. *7*, 963–976.

Tan, T.C.J., Knight, J., Sbarrato, T., Dudek, K., Willis, A.E., and Zamoyska, R. (2017). Suboptimal T-cell receptor signaling compromises protein translation, ribosome biogenesis, and proliferation of mouse CD8 T cells. Proc. Natl. Acad. Sci. U S A *114*, E6117–E6126.

Ude, S., Lassak, J., Starosta, A.L., Kraxenberger, T., Wilson, D.N., and Jung, K. (2013). Translation elongation factor EF-P alleviates ribosome stalling at polyproline stretches. Science *339*, 82–85.

Wiśniewski, J.R., Zougman, A., Nagaraj, N., and Mann, M. (2009). Universal sample preparation method for proteome analysis. Nat. Methods *6*, 359–362.

Woolstenhulme, C.J., Guydosh, N.R., Green, R., and Buskirk, A.R. (2015). High-precision analysis of translational pausing by ribosome profiling in bacteria lacking EFP. Cell Rep. *11*, 13–21.

Xiao, Q., Yan, P., Ma, X., Liu, H., Perez, R., Zhu, A., Gonzales, E., Tripoli, D.L., Czerniewski, L., Ballabio, A., et al. (2015). Neuronal-targeted TFEB accelerates lysosomal degradation of APP, reducing Aβ generation and amyloid plaque pathogenesis. J. Neurosci. *35*, 12137–12151.

Xu, X., Araki, K., Li, S., Han, J.H., Ye, L., Tan, W.G., Konieczny, B.T., Bruinsma, M.W., Martinez, J., Pearce, E.L., et al. (2014). Autophagy is essential for effector CD8(+) T cell survival and memory formation. Nat. Immunol. *15*, 1152–1161.

Yoshikawa, T.T. (2000). Epidemiology and unique aspects of aging and infectious diseases. Clin. Infect. Dis. *30*, 931–933.

Yu, Z., Huang, H., Reim, A., Charles, P.D., Northage, A., Jackson, D., Parry, I., and Kessler, B.M. (2017). Optimizing 2D gas chromatography mass spectrometry for robust tissue, serum and urine metabolite profiling. Talanta *165*, 685–691.

STAR★METHODS

KEY RESOURCES TABLE

REAGENT or RESOURCE	SOURCE	IDENTIFIER
Antibodies		
GAPDH	Merck Millipore	MAB374, RRID:AB_2107445
LC3A/B	Sigma	L8918, RRID:AB_1079382
eIF5A	BD Biosciences	611976, RRID:AB_399397
Hypusine	Merck Millipore	ABS1064, RRID:AB_2631138
Hypusine	Creative Biolabs	PABL-202
ATG7	Abcam	Ab133528, RRID:AB_2532126
ATG3	Abcam	Ab108251, RRID:AB_10865145
H3	Cell Signaling	9715, RRID:AB_331563
S6	Cell Signaling	2317, RRID:AB_2238583
pS6 (Ser235/236)	Cell Signaling	4858, RRID:AB_916156
AMPKα	Cell Signaling	2793, RRID:AB_915794
pAMPKα (T172)	Cell Signaling	2535, RRID:AB_331250
AcK	Cell Signaling	9441, RRID:AB_331805
ATF4	Santa Cruz	sc-200, RRID:AB_2630429
eIF2α	Cell Signaling	2103, RRID:AB_836874
p-eIF2α (Ser51)	Cell Signaling	9721, RRID:AB_330951
TFEB	Bethyl	A303-673A, RID:AB_11204751
TFE3	Sigma	HPA023881
DHS	Abcam	Ab190266
β-Actin	Cell Signaling	3700, RRID:AB_2242334
CD40	Thermo Fisher	16-0401-86, RRID: AB_468943
IRDye 800CW Donkey Anti-Rabbit IgG (H+L)	LI-COR	926-32213, RRID:AB_621848
IRDye 680RD Donkey Anti-Mouse IgG (H+L)	LI-COR	926-68022, RRID:AB_10715072
IRDye 800CW Streptavidin	LI-COR	926-32230,
Goat anti-Rabbit IgG (H+L) Cross-Adsorbed Secondary Antibody, Alexa Fluor 568	Thermo Fisher	A-11011, RRID: AB_143157
CD16/CD32 Monoclonal Antibody (FcR Block)	Thermo Fisher	14-0161-85, RRID:AB_467134
Pacific Blue anti-mouse/human CD44 Antibody	BioLegend	103020, RRID:AB_493683
Pacific Blue anti-mouse TER-119/Erythroid Cells Antibody	BioLegend	116232, RRID:AB_2251160
Pacific Blue anti-mouse CD45.1 Antibody	BioLegend	110722, RRID:AB_492866
Pacific Blue anti-mouse Ly-6G/Ly-6C (Gr-1) Antibody	BioLegend	108430, RRID:AB_893556
Pacific Blue anti-mouse Ly-6A/E (Sca-1) Antibody	BioLegend	122520, RRID:AB_2143237
Brilliant Violet 605 anti-mouse CD41 Antibody	BioLegend	133921, RRID:AB_2563933
Brilliant Violet 605 anti-mouse CD4 Antibody	BioLegend	100451, RRID:AB_2564591
Brilliant Violet 605 anti-mouse/human CD45R/B220 Antibody	BioLegend	103244, RRID:AB_2563312
Ly-6A/E (Sca-1) Monoclonal Antibody, PerCP-Cyanine5.5	Thermo Fisher	45-5981-82, RRID:AB_914372
CD19 Monoclonal Antibody, PerCP-Cyanine5.5	Thermo Fisher	45-0193-82, RRID:AB_1106999
FITC anti-mouse Ly-6A/E (Sca-1) Antibody	BioLegend	108106, RRID:AB_313343
FITC anti-mouse/human CD45R/B220 Antibody	BioLegend	103206, RRID: AB_312991
CD43 Monoclonal Antibody, FITC	Thermo Fisher	11-0431-81, RRID:AB_465039
FITC anti-mouse CD4 Antibody	BioLegend	100406, RRID:AB_312691
FITC anti-mouse IgM Antibody	BioLegend	406506, RRID:AB_315056

(*Continued on next page*)

Continued

REAGENT or RESOURCE	SOURCE	IDENTIFIER
PE anti-mouse/human CD45R/B220 Antibody	BioLegend	103208, RRID:AB_312993
PE anti-mouse Ly-51 Antibody	BioLegend	108308, RRID:AB_313365
PE anti-mouse/human CD44 Antibody	BioLegend	103008, RRID:AB_312959
PE anti-mouse Ly-6G/Ly-6C (Gr-1) Antibody	BioLegend	108408, RRID:AB_313373
PE anti-mouse CD135 Antibody	BioLegend	135306, RRID:AB_1877217
PE anti-mouse CD19 Antibody	BioLegend	115508, RRID:AB_313643
PE anti-mouse/human CD11b Antibody	BioLegend	101208, RRID:AB_312791
CD4 Monoclonal Antibody (GK1.5), PE-Cyanine5	Thermo Fisher	15-0041-82, RRID:AB_468695
PE/Cy5 anti-mouse CD5 Antibody	BioLegend	100610, RRID:AB_312739
PE/Cy5 anti-mouse CD8a Antibody	BioLegend	100710, RRID:AB_312749
PE/Cy5 anti-mouse/human CD11b Antibody	BioLegend	101210, RRID:AB_312793
PE/Cy5 anti-mouse CD11c Antibody	BioLegend	117316, RRID:AB_493566
PE/Cy5 anti-mouse/human CD45R/B220 Antibody	BioLegend	103210, RRID:AB_312995
PE/Cy5 anti-mouse CD3ε Antibody	BioLegend	100310, RRID:AB_312675
TCR beta Monoclonal Antibody, PE	Thermo Fisher	12-5961-82, RRID:AB_466066
TCR gamma/delta Monoclonal Antibody, PE-Cyanine5	Thermo Fisher	15-5711-82, RRID:AB_468804
PE/Cy5 anti-mouse TER-119/Erythroid Cells Antibody	BioLegend	116210, RRID:AB_313711
PE/Cy5 anti-mouse NK-1.1 Antibody	BioLegend	108716, RRID:AB_493590
PE/Cy5 anti-mouse Ly-6G/Ly-6C (Gr-1) Antibody	BioLegend	108410, RRID:AB_313375
PE/Cy7 anti-mouse CD43 Antibody	BioLegend	143210, RRID:AB_2564349
PE/Cy7 anti-mouse CD8a Antibody	BioLegend	100722, RRID:AB_312761
PE/Cy7 anti-mouse CD19 Antibody	BioLegend	115520, RRID:AB_313655
PE/Cy7 anti-mouse IgM Antibody	BioLegend	406514, RRID:AB_10642031
PE/Cy7 anti-mouse CD150 (SLAM) Antibody	BioLegend	115914, RRID:AB_439797
APC anti-mouse CD105 Antibody	BioLegend	120414, RRID:AB_2277914
APC anti-mouse CD3ε Antibody	BioLegend	100312, RRID:AB_312677
TCR beta Monoclonal Antibody, APC	Thermo Fisher	17-5961-81, RRID:AB_469480
TCR gamma/delta Monoclonal Antibody, APC	Thermo Fisher	17-5711-82, RRID:AB_842756
APC anti-mouse NK-1.1 Antibody	BioLegend	108710, RRID:AB_313397
APC anti-mouse/human CD45R/B220 Antibody	BioLegend	103212, RRID:AB_312997
APC anti-mouse/human CD11b Antibody	BioLegend	101212, RRID:AB_312795
APC anti-mouse Ly-6G/Ly-6C (Gr-1) Antibody	BioLegend	108412, RRID:AB_313377
APC anti-mouse CD24 Antibody	BioLegend	138506, RRID:AB_2565651
APC anti-mouse CD4 Antibody	BioLegend	100412, RRID:AB_312697
APC anti-mouse CD48 Antibody	BioLegend	103412, RRID:AB_571997
APC anti-human CD19	BioLegend	302212, RRID:AB_314242
Alexa Fluor® 700 anti-mouse CD45.2 Antibody	BioLegend	109822, RRID:AB_493731
Alexa Fluor® 700 anti-mouse CD8a Antibody	BioLegend	100730, RRID:AB_493703
CD8a Monoclonal Antibody, APC-eFluor 780	Thermo Fisher	47-0081-82
APC/Cy7 anti-mouse/human CD11b Antibody	BioLegend	101226, RRID:AB_830642
APC/Cy7 anti-mouse IgD Antibody	BioLegend	405716, RRID:AB_10662544
APC/Cy7 anti-mouse CD19 Antibody	BioLegend	115530, RRID:AB_830707
CD117 (c-Kit) Monoclonal Antibody (2B8), APC-eFluor 780	Thermo Fisher	47-1171-82, RRID:AB_1272177
Goat Anti-Mouse IgG1, Human ads-AP	Southern Biotech	1070-04
Rabbit IgG (IP)	Santa Cruz	sc-2027, RRID:AB_737197
ATG7 (IP)	Abcam	Ab58735, RRID:AB_940290
AcK (IP)	Cell Signaling	9441, RRID:AB_331805

(*Continued on next page*)

Continued

REAGENT or RESOURCE	SOURCE	IDENTIFIER
**Biological Samples**		
Healthy adult PBMCs	Kennedy Institute of Rheumatology, University of Oxford	Rec: 11/h0711/7<br>UoB Ref: 17-1106
**Chemicals**		
Spermidine	Cayman Chemical	14918
Bafilomycin A1	Cayman Chemical	11038
N1-Guanyl-1,7-diaminoheptane (GC7)	Merck Millipore	259545-10MG
Difluoromethylornithine (DFMO)	Enzo Life Sciences	ALX-270-283
Cycloheximide	Sigma	C1988
4-Hydroxytamoxifen (4-OHT)	Sigma	H6278
Tamoxifen	Sigma	T5648
Torin 1	Cayman Chemical	10997
Etoposide	Cayman Chemical	12092
NP-CGG (4-Hydroxy-3-nitrophenylacetyl hapten-Chicken Gamma Globulin)	Biosearch Technologies	N-5055D
NP-BSA	Biosearch Technologies	N-5050H
**Critical Commercial Assays**		
FlowCellect™ Autophagy LC3 Antibody-based Detection Kit	Merck Millipore	FCCH100171
Click-iT Plus OPP Protein Synthesis Assay	Thermo Fisher	C10456
Click-iT® Metabolic Labeling kits	Thermo Fisher	C10102, C10276
**Deposited Data**		
Ribosome profiling	GEO: GSE133934	
Mendeley data	https://doi.org/10.17632/cd4f7j2hbh.1	
**Experimental Models: Cell lines**		
Jurkat	ATCC	RRID:CVCL_0367
NIH 3T3	ATCC	RRID:CVCL_0594
HEK293T	ATCC	RRID:CVCL_0063
**Experimental Models: Organisms/Strains**		
Mouse: C57BL/6J	The Jackson Laboratory	RRID:IMSR_JAX:000664
Mouse: B6.SJL	The Jackson Laboratory	RRID:IMSR_JAX:100012
Mouse: *Mb1-cre*	The Jackson Laboratory	RRID:IMSR_JAX:020505
Mouse: *Atg7^{flox}*	(Komatsu et al., 2005)	MGI:3587769
Mouse: GFP-LC3	(Mizushima et al., 2004)	MGI:3759813
Mouse: *Dhs^{flox}*	(Pällmann et al., 2015)	NA
Mouse: *Tcfeb^{flox}*	(Settembre et al., 2012)	NA
Mouse: CAG-cre/Esr1	The Jackson Laboratory	RRID:IMSR_JAX:004682
**Oligonucleotides (siRNA)**		
Eif5a #1	Dharmacon	D-057410-02
Eif5a #2	Dharmacon	D-057410-04
Odc #1	Dharmacon	D-041731-17
Odc #2	Dharmacon	D-041731-03
Dhs #1	Dharmacon	D-054953-01
Dhs #2	Dharmacon	D-054953-02
Tfeb	Dharmacon	D-050607-02
Tfe3	Dharmacon	L-054750-00
Non-target	Dharmacon	D-001220-01

(*Continued on next page*)

Continued		
REAGENT or RESOURCE	SOURCE	IDENTIFIER
Recombinant DNA		
pMSCV-UBC:GFP	Addgene	RRID:Addgene_68482
MISSION® 3X LacO Inducible Non-Target shRNA Control	Sigma	SHC332-1EA
TaqMan® probes		
Gapdh	Thermo Fisher	Mm99999915_g1
Odc1	Thermo Fisher	Mm01964631_g1
Dohh	Thermo Fisher	Mm00505991_m1
Atg9b	Thermo Fisher	Mm01157883_g1
Ctsd	Thermo Fisher	Mm00515586_m1
Gla	Thermo Fisher	Mm00516323_m1
Neu1	Thermo Fisher	Mm00456846_m1
Psap	Thermo Fisher	Mm00478338_m1
Tfeb	Thermo Fisher	Mm00448968_m1
Wipi1	Thermo Fisher	Mm00461219_m1
GAPDH	Thermo Fisher	Hs02758991_g1
TFEB	Thermo Fisher	Hs01115721_m1
ATG7	Thermo Fisher	Hs00893770_m1
CHOP	Thermo Fisher	Hs00358796_g1
Software and Algorithms		
FlowJo	FlowJo (commercial)	https://www.flowjo.com
Prism	GraphPad (commercial)	https://www.graphpad.com/scientific-software/prism/
Image Studio Lite	LI-COR (commercial)	https://www.licor.com/bio/image-studio-lite/?gclid=Cj0KCQjwpPHoBRC3ARIsALfx-_JzDOH3Ce3nVzcdgITtx4Xe0ouX62JKl-h0H5WWVPjdEPnrzl3l08waArTQEALw_wcB

LEAD CONTACT AND MATERIALS AVAILABILITY

Further information and requests for resources and reagents should be directed to and will be fulfilled by the Lead Contact, Anna Katharina Simon (katja.simon@kennedy.ox.ac.uk).

EXPERIMENTAL MODEL AND SUBJECT DETAILS

Mice

Young (6-12 weeks) and old (22-24 months) C57BL/6J wild-type mice (RRID:IMSR_JAX:000664) were purchased from Charles River UK. CD45.1^{+} B6.SJL mice (RRID:IMSR_JAX:100012) were purchased from Charles River UK and bred in BSU at the Kennedy Institute of Rheumatology. *Mb1-cre* mice were a kind gift from M Reth, *Atg7*^{flox} mice from M Komatsu (1 year old mice were used in this study). GFP-LC3 mice from N Mizushima and Stefan Balabanov made the bone marrow from inducible *Dhs*^{flox} mice available (Pällmann et al., 2015). *Tcfeb*^{flox} mice from A Ballabio. CAG-cre/Esr1 mice from the Jackson lab. 5 mM Spermidine (Cayman Chemical) was administered to mice in the drinking water, changed three times per week. All mice were held at Biomedical Services, University of Oxford. Animal experiments were approved by the local ethical review committee and performed under UK project licenses PPL 30/2809 and then PPL 30/3388.

Cell Lines

Cells (Jurkat, NIH 3T3, HEK293T) were cultured in RPMI-1640 medium supplemented with 10% FBS, Penicillin-Streptomycin (P/S), and L-Glutamine (all Sigma) in 5% CO_2 at 37°C. Stable transduction of the lentivirus to NIH 3T3 cells was performed as previously described (Preukschas et al., 2012). The IPTG-inducible MISSION shRNA lentiviral vector pLKO-puro-IPTG-3xLacO contains either the shRNA against the 3′-UTR of murine eIF5A mRNA (custom-made from #SHCLND-NM181582-TRCN0000125229, Sigma) or a corresponding non-target shRNA control (#SHC332-1EA, Sigma). 100 μM IPTG (Thermo Fisher) was used to induce the expression of shRNA. Immortalized *Dohh*$^{flox/flox}$ and *Dohh*$^{+/+}$ 3T3 cells were established from mouse embryonic fibroblasts (Sievert et al., 2014). 100 nM 4-OHT (H6278, Sigma) freshly prepared in ethanol was used to induce the knockout of *Dohh* and *Tfeb*.

Human Peripheral Blood
25 mL of blood was obtained from non-fasting healthy donors (old, $\geq$ 65 years of age; young, $\leq$ 65 years of age) in heparinized tubes. All subjects gave written informed consent. The study was approved by the Ethics Committee of the University of Oxford.

METHOD DETAILS

Bone Marrow Chimera
Bone marrow cells were collected from the femur and tibia of the donor mice (*Dhs*$^{+/+}$ *or Dhs*$^{-/-}$ CD45.2^{+}, with young B6.SJL CD45.1^{+} mice as competitors) and frozen in cryopreservation medium for long-term storage. Carefully thawed cells were counted and mixed at the indicated ratio for intravenous (iv) injection. The recipient B6.SJL mice were lethally irradiated (550 cGy twice, 4 hours apart) and rested for 1 hour prior to injection. A total of 1.5 million bone marrow cells were injected intravenously into recipient mice. After at least 16 weeks of long-term reconstitution, tamoxifen (Sigma) dissolved in corn oil was administered via oral gavage (5 mg/mouse/day for 5 consecutive days) and peripheral blood and organs were collected for analysis.

Mouse Immunization
Mice were injected intraperitoneally (ip) with 50 μg NP-CGG (N-5055D-5, Biosearch Technologies) precipitated in Imject Alum adjuvant (Thermo Fisher) on day 0 followed by secondary immunization on or after day 42 (50 μg NP-CGG in PBS ip). Spermidine was given 2 days before the first immunization and then continuously in drinking water. Peripheral blood samples were collected from tail vein on indicated days.

Mouse B Cell Purification and Stimulation
Murine splenic B cells were purified using the Pan B Cell Isolation Kit II (130-104-443, Miltenyi). Cells were cultured at a density of 1 million/ml supplemented with 10 μg/ml LPS (Santa Cruz) for stimulation. Medium was replaced on day 3 of culture by replacing half volume with fresh culture medium containing LPS followed by analysis on day 4 and day 5. For 4-OHT-inducible *Tfeb* knockout B cells, cells were stimulated with 10 ng/mL murine recombinant IL-4 (214-14, PeproTech) and 5 μg/mL anti-mouse CD40 antibody (Thermo Fisher) for 4 days.

Drug Treatments
GC7 (Merck Millipore) was added for 24 hours to B cells (10 μM) on day 2 after B cell stimulation (unless otherwise indicated) or to Jurkat/NIH 3T3 cells (100 μM). 10 nM bafilomycin A1 (Cayman Chemical), 10 μg/ml cycloheximide (Sigma), or 100 nM Torin 1 (Cayman Chemical) were added to cells for 2 hours. 10 μM etoposide (Cayman Chemical) or 1 μM thapsigargin (Cayman Chemical) was added to cell culture for 6 hours, or 1 mM difluoromethylornithine (DFMO, Enzo Life Sciences) for 24 hours.

One- and Two-Dimensional Western Blot
For one-dimensional western blot, cells were lysed using NP-40 lysis buffer containing proteinase inhibitors (Sigma) and phosphatase inhibitors (Sigma) on ice. After spinning down the debris, protein concentration in the supernatant was quantified by BCA Assay (23227, Thermo Fisher). Reducing Laemmli Sample Buffer was then added to the supernatant and heated at 100°C for 5 minutes. 5-20 μg proteins were loaded for SDS-PAGE analysis. NuPAGE Novex 4%–12% Bis-Tris gradient gel (Thermo Fisher) with MES running buffer (Thermo Fisher) was used. To improve separation of LC3-I and LC3-II, 15% Tris-HCl gel and SDS running buffer was used. Proteins were transferred to a PVDF membrane (IPFL00010, Merck Millipore) and blocked with 5% skimmed milk-TBST. Membranes were incubated with primary antibodies dissolved in 1% milk overnight and secondary antibodies dissolved in 1% milk with 0.01% SDS for imaging using the Odyssey CLx Imaging System. Two-dimensional western blot was carried out as described before (Preukschas et al., 2012). Data were analyzed using Image Studio Lite or Fiji.

Immunoprecipitation
After treatments an equal number of cells were collected, washed with PBS and lysed using NP-40 lysis buffer with proteinase and phosphatase inhibitors on ice for 20 minutes. 950 μL lysis buffer was used for 25 million Jurkat cells. Cell debris was centrifuged down. 50 μL supernatant was collected as whole cell lysate (input control), and the protein concentration was measured by BCA assay. The remaining supernatant was mixed with 15 μL Protein A Agarose beads (Thermo Fisher) in PBS, then rotated at 4°C for 15 minutes to pre-clear the lysate. The beads were removed by centrifugation at 6000 rpm for 1 minute. Immunoprecipitation (IP) antibody (AcK, ATG7, or rabbit IgG) and 20 μL Protein A agarose beads were added to the lysate and then rotated at 4°C overnight. The beads were washed twice with NP-40 lysis buffer containing proteinase and phosphatase inhibitors and the supernatant discarded. 35 μL 2X Reducing Laemmli Sample Buffer was added to the beads and heated at 100°C for 5 minutes, and supernatant used for western blot analysis.

ELISA
For NP-IgG1 ELISA, ELISA plates (675061, Greiner Bio-One) were coated with 5 μg/ml NP-BSA (N-5050H-10, Biosearch Technologies) in bicarbonate/carbonate buffer at 4°C overnight. After three washes with PBS, plates were blocked with 5% skimmed

milk in PBS at 37°C for 1 hour, followed by 3 x PBS washes. Serum samples diluted in 1% milk were added and incubated at 37°C for 1 hour. For relative quantification, a standard serum sample was made by pooling samples on day 7 post secondary immunization from a prior experiment, which was used for all subsequent experiments. Serum samples of various days were serially diluted first to determine the proper dilution, and 1:1000-1:5000 was chosen for NP-IgG1 ELISA. After serum sample incubation, plates were washed 6 times with PBS-0.05% Tween 20 followed with detection antibody incubation at 37°C for 1 hour. Alkaline phosphatase-(AP-) conjugated goat-anti-mouse IgG1 (Southern Biotech) detection antibody was diluted in 1% milk-PBS at a ratio of 1:2000. After 5 washes with PBS-0.05% Tween 20 and once with PBS, AP-substrate (S0942, Sigma) dissolved in pNPP buffer was added for 15-20 min and absorbance was measured at 405 nm by ELISA plate reader (FLUOstar Omega, BMG Labtech). To detect IgM in culture supernatants, samples were diluted at the ratio of 1:100. The IgM Mouse Uncoated ELISA Kit (88-50470, Thermo Fisher) was used according to manufacturer's protocol.

ELISpot

MultiScreenHTS-HA filter plates (MSHAS4510) were first rinsed with 35% ethanol for 30 s and washed 3 times with PBS. Plates were coated with 20 μg/ml NP-BSA in PBS at 4°C over night. After three washes with PBS, plates were blocked with RPMI-1640 medium supplemented with 10% FBS at 37°C for 30 minutes. Then bone marrow cells were added in duplicates in culture medium at the density of $3x10^5$ /100 μL/ well and cultured at 37°C over night. Plates were then washed 3 times with PBS and 3 times with PBS-0.05% Tween 20. AP-conjugated anti-mouse IgG1 detection antibody diluted in 1% FBS was added to plates for 1 hour incubation at 37°C. After 5 washes with PBS-0.05% Tween 20 and once with PBS, AP substrate (170-6432, Bio-Rad) was added for spot development. Plates with clear spots and clean background were washed with water to stop development, dried, and counted with the AID ELISpot Reader System (ELR078IFL, AID GmbH).

Plasmid Construction

The TFEB polyproline motif-mCherry-IRES, 13P-mCherry-IRES, Random-mCherry-IRES, murine wild-type/mutant (PPP to AAA) *Tfeb*-HA were cloned into pMSCV-UBC:GFP. A flexible GSGSG linker was inserted between the targeted sequence and mCherry to assist folding of the fusion protein. The sequence coding C-terminal 37-amino acid degron of murine ODC (Kelly et al., 2007) was inserted to the C-terminal of mCherry. The nuclear localization sequence RRRR (AGAAGACGCAGG) of TFEB was mutated to AAAA (GCTGCAGCCGCG) by PCR.

Transfection

NIH 3T3 cells were transfected with siRNA according to Thermo Fisher protocol (13778075, Lipofectamine® RNAiMAX Reagent). Cells were collected on day 3 post-transfection for western blot analysis. For plasmid transfection assay, NIH 3T3 cells were transfected with the indicated plasmids using Lipofectamine 3000 according to the manufacturer's protocol. Protein expression was quantified by flow cytometry (geometric mean fluorescence intensity) or western blot 24 hours post transfection.

Flow Cytometry

Cells were stained with fixable Zombie Aqua Live/Dead staining (423102, Biolegend), FcR block, and surface marker antibodies and analyzed with four-laser LSR Fortessa X-20. Acquired data were analyzed using FlowJo 10.2. For LC3-II flow cytometry staining, the FlowCellect™ Autophagy LC3 Antibody-based Detection Kit (FCCH100171, Merck Millipore) and for CellTrace staining, CellTrace Violet (C34557, Thermo Fisher) were used according to the manufacturer's protocol.

CytoID Staining

Cells were stained with CytoID (ENZ-51031-K200, Enzo Life Sciences) according to manufacturer's protocol. Briefly, cells were resuspended in CytoID staining solution (1:4000 diluted in RPMI without phenol red (R1780, Sigma) supplemented with 5% FBS) and incubated at 37°C for 30 minutes in the dark. Then cells were washed once, followed by surface marker staining and flow cytometry analysis without fixation.

Quantitative PCR

RNA was extracted using the RNeasy Plus Mini Kit (74134, QIAGEN). The concentration of RNA was measured using NanoDrop1000 (Thermo Fisher), followed by reverse transcription using the High Capacity RNA-to-cDNA Kit (4387406, Thermo Fisher). Taqman probes (Thermo Fisher), TaqMan Gene Expression Master Mix (4369016, Thermo Fisher), and the ViiA 7 Real-Time PCR System (Thermo Fisher) were used for quantitative PCR. ΔΔCt method was used for the quantification of target mRNAs expression using *Gapdh* as the reference gene.

Confocal Microscopy

B cells from GFP-LC3 mice were purified by magnetic-activated cell sorting (MACS) and fixed with 4% paraformaldehyde at room temperature for 10 min. After nuclear staining with DAPI (Sigma), cells were transferred to slides using Cytospin 3 cytocentrifuge (Shandon) and imaged with the Olympus FV1200 Laser Scanning Microscope. CellProfiler software was used for automatic autophagosome quantification. Nuclei were defined in DAPI channel (diameter of 45-100 pixel units), autophagosomes were defined in GFP

channel (diameter of 3-15 pixel units,1 pixel unit = 100 nm). An artificial but consistent threshold of 1-10 cellular GFP spots in GFP-LC3 confocal imaging was used for statistical analysis. 10%–45% B cells are GFP-negative in GFP-LC3 transgenic mice as assessed by flow cytometry. Therefore cells with 0 GFP spot (either due to no GFP expression or very low autophagy) were excluded from all samples. Cells with more than 10 GFP spots were also excluded from all samples as outliers.

Cell Fractionation

Cells in a 1.5 mL Eppendorf tube were washed once with cold PBS and re-suspended in 200-500 μL HLB buffer (10 mM Tris-HCl pH 7.5, 10 mM NaCl, 2.5 mM MgCl2) with protease inhibitors and phosphatase inhibitors. Then an equal volume of HLB+2N buffer (HLB+0.4% NP-40, 1N is 0.2% NP-40) was added and carefully mixed. Samples were incubated on ice for 5 minutes and then underlayed with 200 μL HLB+NS buffer (HLB+0.2% NP-40+10% sucrose). Samples were centrifuged at 500 g for 5 minutes and the upper half of the supernatant was collected as the cytoplasmic fraction. The remaining liquid was discarded and the nuclear pellet was washed once more with HLB buffer and lysed with NP-40 lysis buffer or Laemmli sample buffer. All procedures were performed on ice.

Protein Mass Spectrometry

LPS-stimulated mouse B cells were treated with 10 μM GC7 on day 2 for 24 hours. Cells were collected and fractionated for label-free quantitative mass spectrometry (MS) analysis. For SILAC MS, B cells stained with CellTrace Violet (C34557, Thermo Fisher) were cultured in two types of medium: light and heavy. In light medium, SILAC RPMI-1640 medium (89984, Thermo Fisher) supplemented with 1.15 mM L-Arg (Sigma) and 0.22 mM L-Lys (Sigma), 10% dialyzed FBS (Sigma), and P/S, L-Glutamine, 50 μM 2-mercaptoethanol, 20 mM HEPES were used. In heavy medium, $^{13}C_6{}^{15}N_4$-L-Arg (Arg-10, Silantes) and $^{13}C_6{}^{15}N_2$-L-Lys (Lys-8, Silantes) were used instead of the light arginine and lysine respectively. 10 μM GC7 was added to either light or heavy medium in two repeats. After GC7 treatment, dead cells were first removed using the Dead Cell Removal Kit (Miltenyi). An identical number of cells that had divided at least 4 times in light or heavy medium were collected by FACS, and mixed for cell fractionation and protein MS analysis.

Peptide samples were prepared using the Filter Aided Sample Preparation (FASP), as previously described (Wiśniewski et al., 2009). Briefly, Vivacon 500 filters (Sartorius, VN01H02 10 kDa/VNCT01) were pre-washed with 200 μL 0.1% trifluoroacetic acid in 50% acetone. Lysate samples were loaded to the filter and denatured with 200 μL 8 M urea in 100 mM triethylammonium bicarbonate buffer (TEAB) for 30 minutes at room temperature. Denatured proteins were reduced by 10 mM tris (2-carboxyethyl) phosphine (TCEP) for 30 minutes at room temperature and alkylated with 50 mM chloroacetamide for 30 minutes at room temperature in the dark. Subsequently, 1 μg LysC (Wako) in 150 μL 50 mM TEAB containing 6 M urea was added and incubated at 37°C for 4 hours. Then the buffer was diluted to 2 M urea by 50 mM TEAB, followed by adding 0.5 μg trypsin (Promega) overnight at 37°C. Trypsinised samples were centrifuged and the flow-through, containing peptides, was dried and resuspended in 70 μL 10% formic acid (or 5% formic acid and 5% DMSO for SILAC experiment).

Peptides were separated on an Ultimate 3000 UHPLC system (Thermo Fisher) and electrosprayed directly into a QExactive mass spectrometer (Thermo Fisher). The peptides were trapped on a C18 PepMap100 pre-column (300 μm i.d. x 5 mm, 100 Å, Thermo Fisher) using solvent A (0.1% formic acid in water) at a pressure of 500 bar, then separated on an in-house packed analytical column (75 μm i.d. packed with ReproSil-Pur 120 C18-AQ, 1.9 μm, 120 Å, Dr. Maisch GmbH) using a linear gradient (length: 120 minutes, 15% to 35% solvent B (0.1% formic acid in acetonitrile), flow rate: 200 nl/min). Data were acquired in a data-dependent mode (DDA). Full scan MS spectra were acquired in the Orbitrap (scan range 350-1500 m/z, resolution 70000, AGC target 3e6, maximum injection time 50 ms). The 10 most intense peaks were selected for HCD fragmentation at 30% of normalized collision energy at resolution 17500, AGC target 5e4, maximum injection time 120 ms with first fixed mass at 180 *m/z*. Charge exclusion was selected for unassigned and 1+ ions. The dynamic exclusion was set to 20 s.

Raw MS data were processed by MaxQuant (version 1.5.0.35i) for peak detection and quantification (Cox and Mann, 2008; Cox et al., 2011). MS spectra were searched against the *Mus musculus* UniProt Reference proteome (retrieved 12/01/17) alongside a list of common contaminants, using the Andromeda search engine with the following search parameters: full tryptic specificity, allowing two missed cleavage sites, fixed modification was set to carbamidomethyl (C) and the variable modification to acetylation (protein N terminus) and oxidation (M). The search results were filtered to a false discovery rate (FDR) of 0.01 for proteins, peptides and peptide-spectrum matches (PSM). Protein intensity distributions were log2 transformed and median-centered using Perseus (version 1.5.5.3). For the SILAC analysis, missing values were replaced by an estimated background noise value. Proteins without greater-than-background values in both replicates for at least one condition were removed. MA plots were generated using R (version 3.4.2). Reviewed autophagy proteins were searched against the *Mus musculus* UniProt database. Data from the independently analyzed cytoplasmic and nuclear proteins were shown as overlaid MA-plots wherein the log2 average (GC7 + Vehicle) intensity (A) is plotted on *x* versus the log2 (GC7:Vehicle) fold change (M) plotted on *y*.

Spermidine Measurement by Gas Chromatography Mass Spectrometry (GC-MS)

Cells were washed with PBS and the pellet resuspended in lysis buffer (80% methanol + 5% Trifluoroacetic acid) spiked with 2.5 μM 1,7-diaminoheptane (Sigma). The cell suspension, together with acid-washed glass beads (G8772, Sigma), was transferred to a bead beater tube and homogenized in a bead beater (Precellys 24, Bertin Technologies) for four cycles (6500 Hz, 45 s) with 1 minute of ice incubation between each cycle. The homogenized samples were centrifuged at 13,000 g for 20 minutes at 4°C. The supernatant was

collected and dried overnight. For chemical derivatization, 200 μL trifluoroacetic anhydride was added to the dried pellet and incubated at 60°C for 1 hour, shaking at 1200 rpm. The derivatization product was dried, re-suspended in 30 μL isopropanol and transferred to glass loading-vials. The samples were analyzed using a GCxGC-MS system as described (Yu et al., 2017). The following parameters were used for quantification of the 1D-GC-qMS data: type: area, slope: 1000/min, width: 0.04 s, drift 0/min and T. DBL: 1000 min without any smoothing methods used. Cellular spermidine amount was normalized to total protein levels determined by BCA assay for each sample first followed by comparison between samples.

OPP-Click Assay

Protein synthesis rate was measured using the Click-iT Plus OPP Protein Synthesis Assay (C10456, Thermo Fisher) according to manufacturer's protocol. Geometric mean fluorescence intensity (normalized to vehicle) was used as an indicator of the relative translation rate.

AHA Labeling of Nascent Proteins

Click-iT® Metabolic Labeling kits (C10102, C10276, Thermo Fisher) were used for AHA labeling according to manufacturer's protocol. Briefly, cells were pre-cultured in methionine-free culture media (R7513, Sigma) supplemented with 10% dialyzed FBS, P/S, L-Cystine, and L-Glutamine for 30 minutes to deplete methionine reserves. 50 μM AHA was then added for 4 hours. Cycloheximide (10 μg/mL, Sigma) was added 30 min before adding AHA to inhibit translation. Cells were lysed in lysis buffer (50 mM Tris-HCl, pH 8.0, 1% SDS with protease/phosphatase inhibitors). The cleared cell lysate (200 μg protein/sample) was used to perform Click reaction for biotin labeling. Biotinylated proteins were directly used for western blot to determine the overall protein synthesis rate or pulled down with streptavidin-agarose beads (50 μL beads/sample, S1638, Sigma) overnight to purify nascent proteins for western blot analyses.

Ribosome Profiling

The sequencing library was prepared according to the reference (Ingolia, 2010; Ingolia et al., 2013) with the following changes. LPS-activated B cells were MACS-purified with the Dead Cell Removal Kit (Miltenyi) to remove dead cells prior to cell lysis. 100 μg/ml cycloheximide was used in the lysis buffer. The B cell lysate was spiked with the *S. cerevisiae* lysate at the ratio of 13 million B cells: 270 million yeast cells (which equals to 20:4 μg miRNA). Clarified B cell and yeast lysates were RNase I-digested, ribosome purified and miRNA extracted to determine the miRNA yield for spiking. Size exclusion chromatography (27-5140-01, GE Healthcare) was used to recover ribosomes. The miRNeasy Micro Kit (QIAGEN) was used to purify miRNAs. Ribosome protected fragments (RPFs) of 27-30 nt size were purified by gel extraction. The Ribo-Zero Gold rRNA Removal Kits (illumina, MRZG126, MRZY1306) were used to deplete ribosomal RNAs, in which the Ribo-Zero Removal Solutions from the mouse and yeast kits were mixed at the ratio of 8 μL: 4 μL to remove both mouse and yeast rRNAs. The following barcoding primers were used for next generation sequencing. 1. 5′-AATGATACGGCGACCACCGAGATCTACACGATCGGAAGAGCACACGTCTGAACTCCAGTCACATGCCATCCGACGATCATTGATGG. 2. 5′-AATGATACGGCGACCACCGAGATCTACACGATCGGAAGAGCACACGTCTGAACTCCAGTCACTGCATCTCCGACGATCATTGATGG. The libraries were sequenced at the Biopolymers Facility, Harvard Medical School.

For the purposes of alignment, the reference genome assemblies for *Mus musculus* (GRCM38) and *Saccharomyces* cerevisiae (SacCer3) were concatenated to create a combined genome fasta file. Prior to alignment, adaptor sequence (ATCTCGTATGCCGTCTTCTGCTTG) was removed from demultiplexed reads and low quality reads (Phred score < 20) were discarded. To remove contaminating rDNA reads, reads were aligned to the combined rDNA genome using bowtie2 and all unaligned reads were kept for alignment to the combined genome. Non-rDNA reads were aligned to the combined genome using bowtie2. All post-alignment analysis was performed using custom scripts written in R. P-site offsets for each read-length from 26 to 35 nts in the RPF libraries were determined by generating metagenes relative to the START codon and applied to aligned reads. For all metagenes, transcripts with no reads within the window of interest were excluded from analysis before applying any other filters. Additionally, for each position in the windows of the 5′ and 3′ metagenes, the highest and lowest 0.1% of signals were removed from analysis.

Human B Cell Assays

Peripheral blood mononuclear cells (PBMCs) were isolated by density gradient centrifugation using Ficoll-Paque. PBMCs were counted using trypan blue. They were either freshly used or frozen until further use. Thawed cells were placed in full medium overnight to allow selection of viable cells. PBMCs were washed with PBS and lysed using NP-40 lysis buffer containing proteinase inhibitors and phosphatase inhibitors on ice for western blot assay or using lysis buffer (80% methanol + 5% Trifluoroacetic acid) spiked with 2.5 μM 1,7-diaminoheptane (Sigma) for spermidine measurement by GC-MS. B cells were sorted using a negative selection kit (B Cell Isolation Kit II, human, Miltenyi Biotec) according to the manufacturer's protocol. B cells (5×10^5 cells) were seeded in 24-well plates, then activated with anti-IgM (5 μg/ml, Jackson Immuno Research) and CD40L (100 ng/ml, Enzo Life science) and treated with 1 mM DFMO, 10 μM spermidine, or 10 μM GC7 for 7 days. Cells then were lysed for western blot or stained for LC3-II measurement by flow cytometry. IgG release in culture supernatants was measured by heterologous two-site sandwich ELISA, according to the manufacturer's protocol (Invitrogen).

QUANTIFICATION AND STATISTICAL ANALYSIS

Prism software (GraphPad) was used for statistical analyses. Data are represented as mean ± SEM. All data points refer to the measurement of distinct samples (biological repeats). Unpaired two-tailed Student's t test was used for comparisons between two normally distributed datasets with equal variances unless specified. One-tailed Student's t test was used when the null hypothesis has a clear direction according to current knowledge (e.g., spermidine is an autophagy inducer in multiple systems or Bafilomycin A1 treatment leads to accumulation of LC3-II). Welch's unequal variances t test was used when the two normally distributed datasets have unequal variances. Holm-Sidak method was used to adjust P values of a family of multiple t tests with a single null hypothesis. Paired or unpaired one/two-way ANOVA, followed with post hoc Sidak's test (comparing two means within each group), Dunnett's test (comparing multiple means with the single control within each group), or Tukey's test (comparing the mean of each sample with the mean of every other sample), was used for multiple comparisons of normally distributed datasets with one/two variables. Mann-Whitney U test was used for comparisons between non-parametric datasets. Linear regression with 95% confidence interval was used to assess the relationships between age and the expression of target proteins or spermidine levels, in which R^2 was used to assess the goodness of fit and the P value calculated from F test was used to assess if the slope was significantly non-zero. P value was used to quantify the statistical significance of the null hypothesis testing. *$p \leq 0.05$, **$p \leq 0.01$, ***$p \leq 0.001$, ****$p \leq 0.0001$.

DATA AND CODE AVAILABILITY

Original images are available at https://doi.org/10.17632/cd4f7j2hbh.1

Sequencing results have been deposited to GEO with the accession number GSE133934.

Molecular Cell, Volume *76*

Supplemental Information

Polyamines Control eIF5A Hypusination, TFEB Translation, and Autophagy to Reverse B Cell Senescence

Hanlin Zhang, Ghada Alsaleh, Jack Feltham, Yizhe Sun, Gennaro Napolitano, Thomas Riffelmacher, Philip Charles, Lisa Frau, Philip Hublitz, Zhanru Yu, Shabaz Mohammed, Andrea Ballabio, Stefan Balabanov, Jane Mellor, and Anna Katharina Simon

Figure S1. Related to Figure 1. 6-week Treatment with Spermidine does not Affect Hematopoiesis in Old Mice.

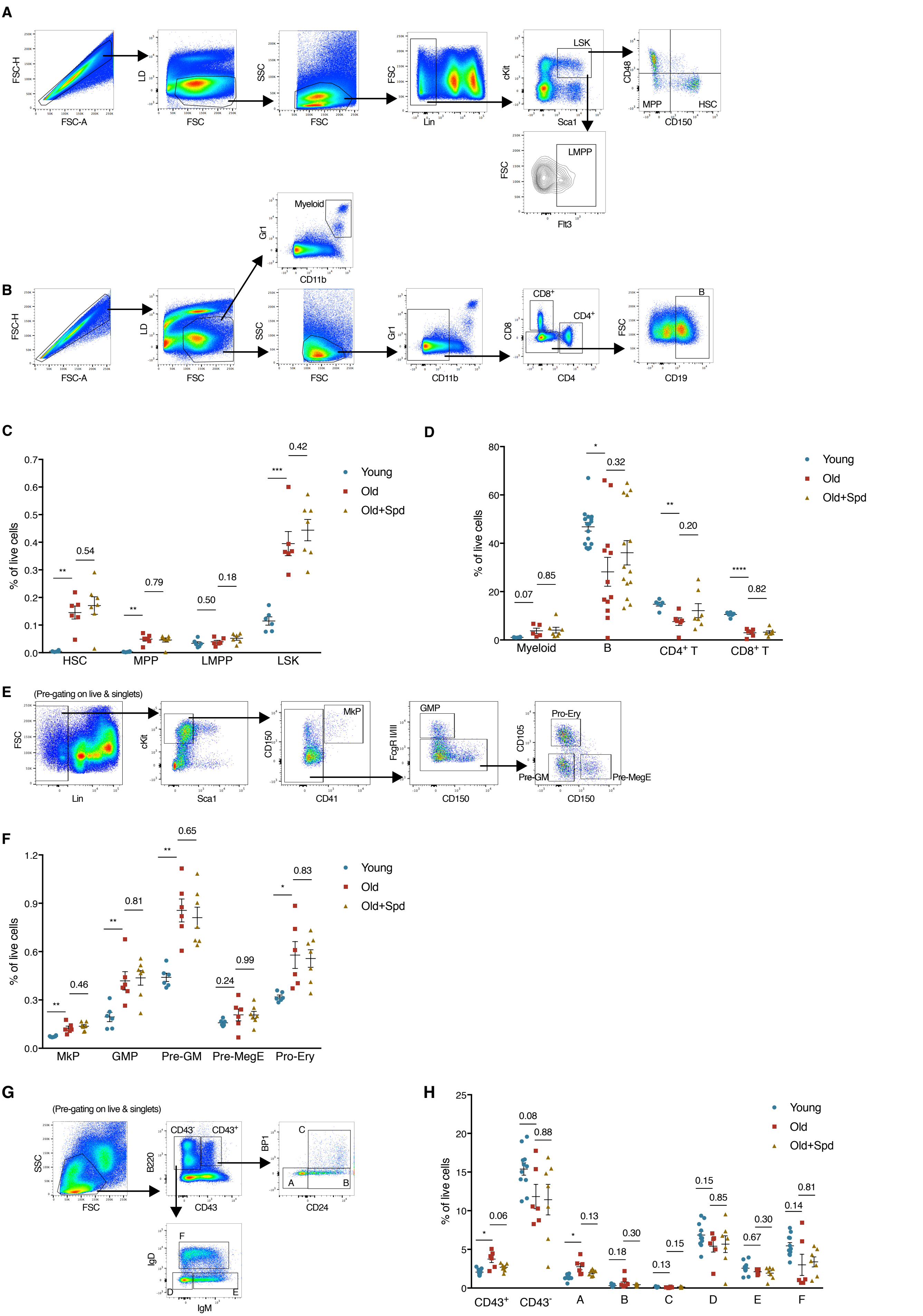

Figure S1. Related to Figure 1. 6-week Treatment with Spermidine does not Affect Hematopoiesis in Old Mice.

Various hematopoietic cell types in bone marrow and spleen from young (12 weeks), old (22-24 months), and old mice continuously administered with spermidine in drinking water for 6 weeks as in Figure 1A were assessed by flow cytometry.

(A) Gating strategy for hematopoietic stem cells (HSCs), multipotent progenitors (MPPs), lymphoid-biased multipotent progenitors (LMPPs), and $Lin^-Sca1^+cKit^+$ cells (LSKs, enriched of hematopoietic stem and progenitor cells) in bone marrow.

(B) Gating strategy for myeloid cells, B cells, $CD4^+$ T cells, and $CD8^+$ T cells in spleen.

(C) Expanded phenotypic HSCs, MPPs, and LSKs in bone marrow from old mice. The abundance of indicated cell types as % of total live cells is shown. n = 6-7 mice.

(D) Spleen lineages are lymphopenic in old mice. n = 6-17 mice as indicated by dots, combined from 2 independent experiments.

(E/F) Expanded myeloid progenitors in bone marrow from old mice. (E) Gating strategy for megakaryocyte progenitors (MkPs), granulocyte-macrophage progenitors (GMPs), pre-granulocyte/macrophages (Pre-GMs), pre-megakaryocytes/erythrocytes (pre-MegEs), and pro-erythroblast cells (Pro-Erys). (F) The abundance of indicated cell types is shown. n = 6-7 mice.

(G/H) Accumulated pro-B cells in old mice. (G) Gating strategy for Hardy fractions A-F. (H) The abundance of indicated cell types is shown. n = 7-11 mice, pooled from 2 independent experiments.

Each dot represents one mouse. Data represented as mean ± SEM. Welch's t-test. *P≤0.05, **P≤0.01, ***P≤0.001, ****P≤0.0001.

Figure S2. Related to Figure 2. Spermidine does not Improve B Cell Responses in Young Mice.

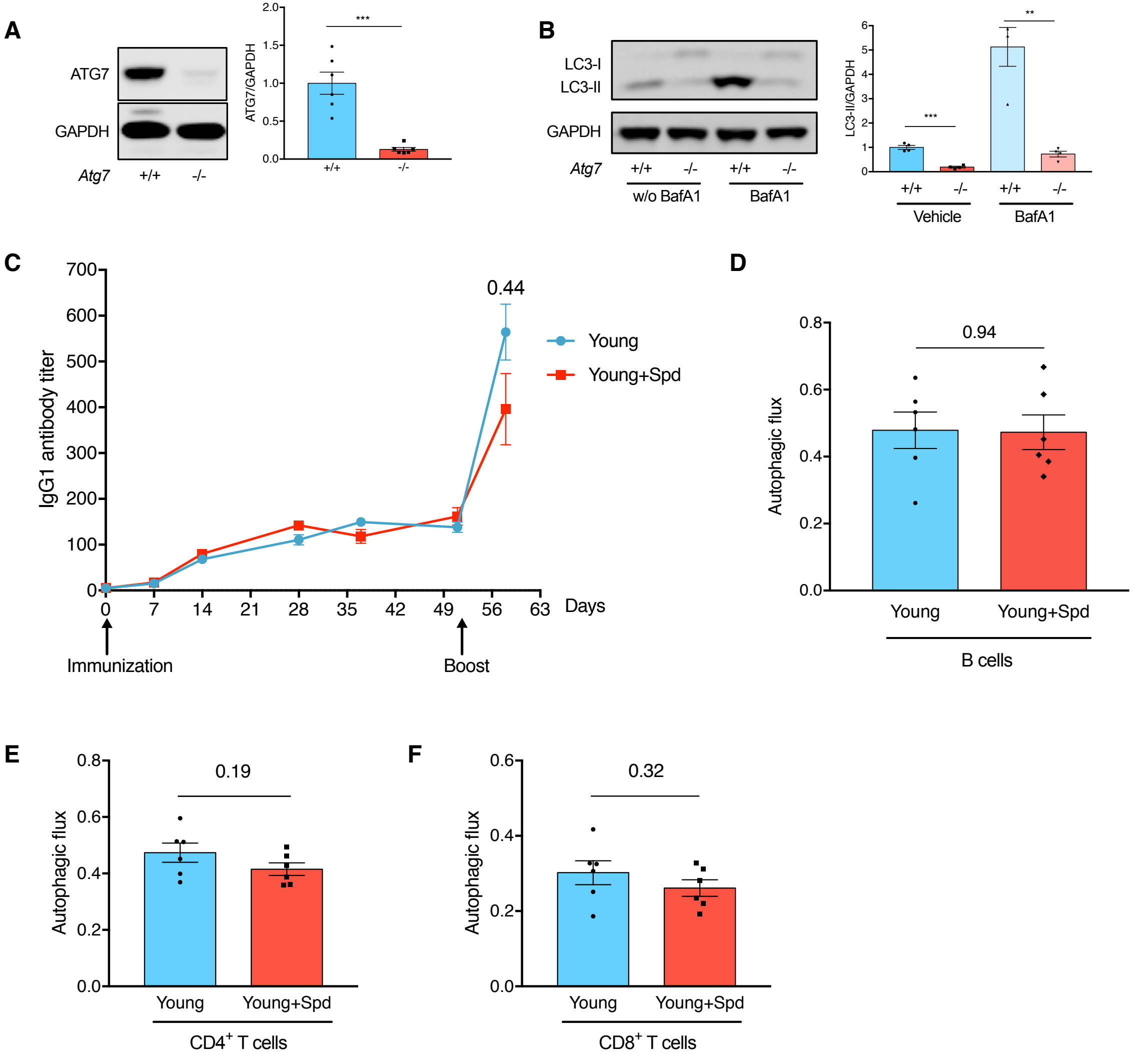

Figure S2. Related to Figure 2. Spermidine does not Improve B Cell Responses in Young Mice.

(A/B) Confirmation of reduced ATG7 expression and autophagy in B cells purified from B cell-specific *Atg7*-knockout mice. (A) B cells were purified from *Mb1-Cre-*, *Atg7*$^{flox/flox}$ (+/+) and *Mb1-Cre+*, *Atg7*$^{flox/flox}$ (-/-) mice and the expression of ATG7 was assessed by Western blot. n = 6 mice. (B) Purified B cells in (A) were cultured with 10 nM BafA1 for 2 h for LC3-II measurement by Western blot. n = 4 mice.

(C-F) Spermidine does not improve IgG1 responses in young mice. (C) Young adult mice were immunized and boosted with NP-CGG. Spermidine administration and serum NP-specific IgG1 measurement were processed as in Figure 2D. n = 11 (D7), 19 (D14), 12 (D28), 9 (D37), 15 (D51) and 6 (D58) mice, combined from 3 independent experiments. (D) Autophagic flux of splenic B cells (B220$^+$CD19$^+$) (D), CD4$^+$ T cells (E), and CD8$^+$ T cells (F) from mice culled on D58 in (C) was measured by LC3-II flow cytometry staining as in Figure 1A. n = 6 mice.

Data represented as mean ± SEM. One-tailed Welch's t-test (A/B). Two-tailed Student's t-test (C-F). **P≤0.01, ***P≤0.001.

Figure S3. Related to Figure 3. Spermidine does not Directly Inhibit HAT Activities and High-dose Spermidine Induces Cellular Stress.

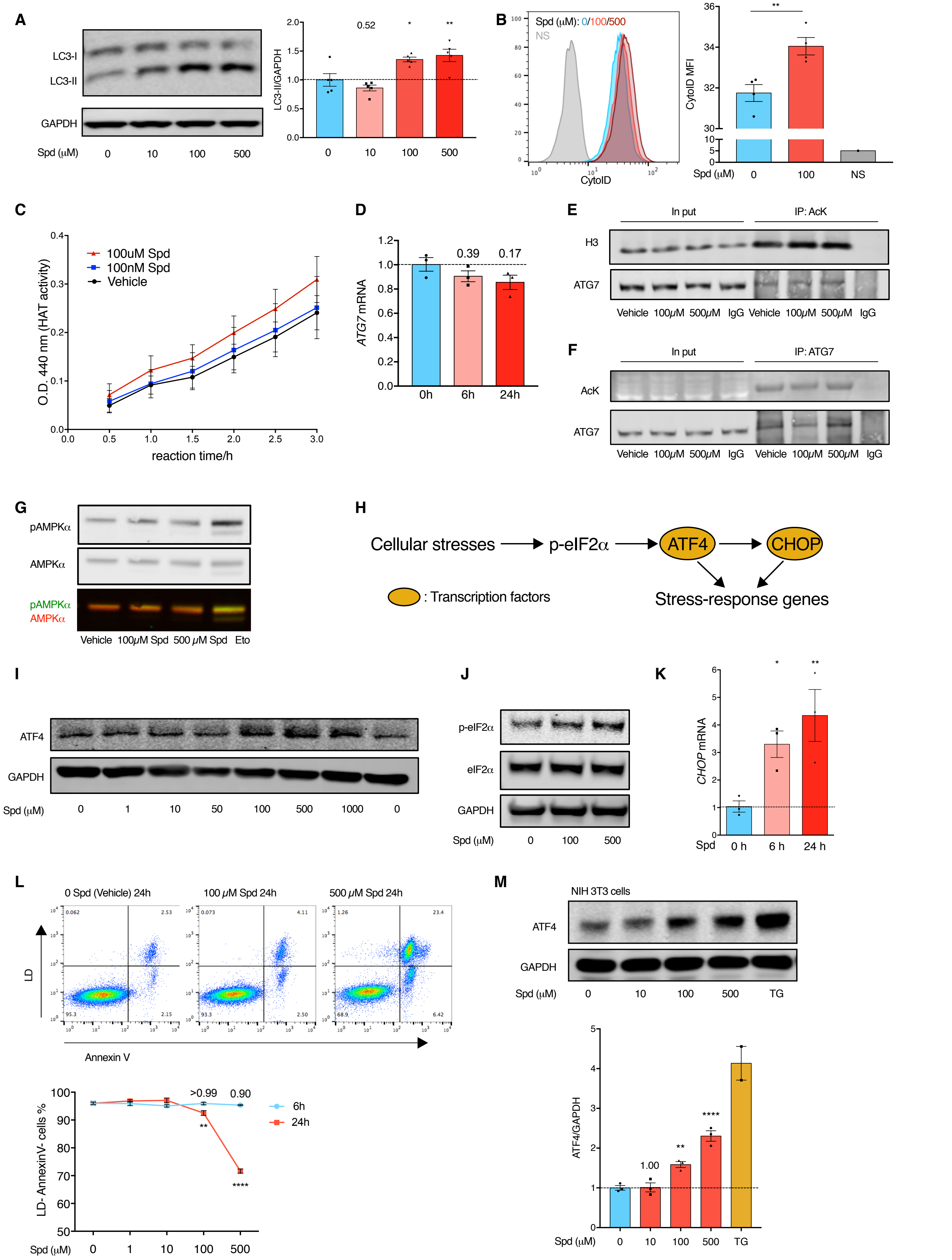

Figure S3. Related to Figure 3. Spermidine does not Directly Inhibit HAT Activities and High-dose Spermidine Induces Cellular Stress.

(A/B) Spermidine induces autophagy *in vitro*. (A) Jurkat cells were treated with spermidine as indicated for 6 h. Autophagy was assessed by LC3-II Western blot. n = 5. (B) Autophagosome/autolysosome-specific staining of CytoID was measured by flow cytometry. n = 4.

(C-F) Spermidine does not inhibit HAT activity in Jurkat cells. (C) Jurkat nuclear extract was prepared using the Nuclear/Cytosol Fractionation Kit, incubated with spermidine of indicated concentrations (starting from 0 h) and the relative HAT activity was measured using the HAT Activity Colorimetric Assay Kit. n = 3. (D) Jurkat cells were treated with 100 μM spermidine for 6 h or 24 h. *ATG7* mRNA was measured by quantitative PCR (qPCR) with *GAPDH* used as the reference gene. n = 3. (E/F) Jurkat cells were treated with spermidine for 6 h and cellular proteins with acetylated lysine residues were pulled down (IP: AcK) and assessed for H3 and ATG7 acetylation (E). To assess ATG7 acetylation in an alternative way, ATG7 was pulled down (IP: ATG7) and acetylation measured with an antibody against AcK (F).

(G) Spermidine does not affect AMPK activity. Jurkat cells were treated with spermidine or etoposide (Eto) for 6 h. AMPK activity was assessed by AMPKα phosphorylation. Representative of 3 independent repeats.

(H-M) High-dose spermidine induces cellular stress. (H) Schematic overview of the integrated stress response eIF2α-ATF4-CHOP pathway. Multiple cellular stresses induce the phosphorylation of eIF2α. Phosphorylated eIF2α facilitates the synthesis of the transcription factor ATF4, which then induces the expression of CHOP. ATF4 and CHOP induce the expression of multiple stress-response genes including chaperones, apoptosis, and autophagy. (I/J) Jurkat cells were treated with spermidine for 6 h. The expression of cellular stress markers ATF4 (I) and phosphorylation of eIF2α (J) were assessed by Western blot. Representative of 3 independent repeats. (K) Jurkat cells were treated with 100 μM spermidine. The expression of *CHOP* was assessed by qPCR with *GAPDH* as the reference gene. n = 3. (L) Jurkat cells were treated with spermidine of indicated concentrations for 6 h or 24 h. Cell viability and apoptosis were assessed by Live-Dead (LD) and Annexin V flow cytometry staining. n = 3. (M) NIH 3T3 cells were treated with spermidine or thapsigargin (TG) for 6 h. The expression of ATF4 was assessed by Western blot. n = 3.

Data represented as mean ± SEM. One-way ANOVA with post hoc Dunnett's test (A/D/K/M). Student's t-test (B). Two-way ANOVA with post hoc Dunnett's test (L). *P≤0.05, **P≤0.01, ****P≤0.0001.

Figure S4. Related to Figure 3. Spermidine Maintains Cellular Autophagy by Hypusinating eIF5A.

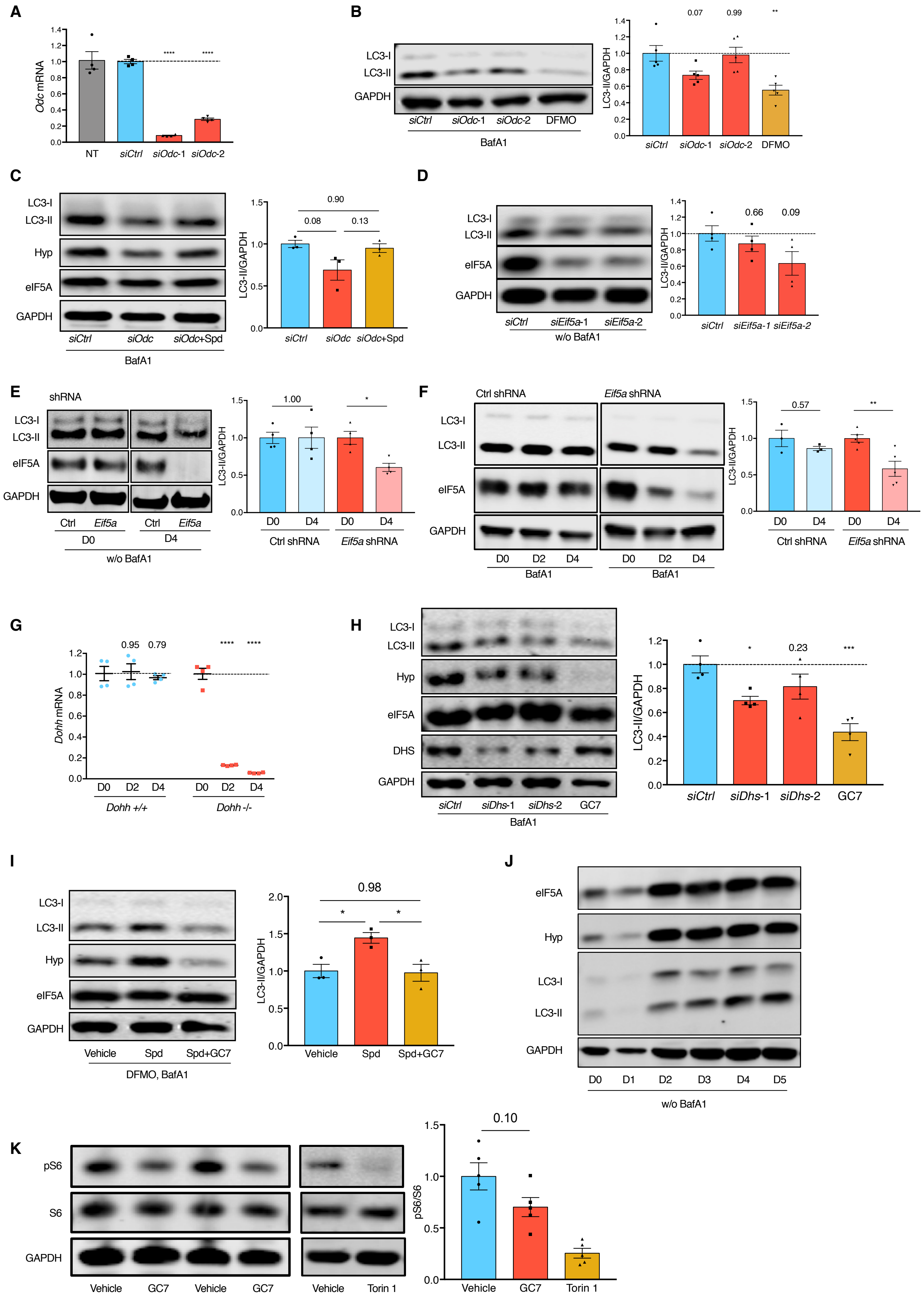

Figure S4. Related to Figure 3. Spermidine Maintains Cellular Autophagy by Hypusinating eIF5A.

(A) NIH 3T3 cells were transfected with non-targeting siRNA (*siCtrl*) or siRNA targeting two different regions of *Odc* mRNA (*siOdc*-1/2) for 3 days. The expression of *Odc* was assessed by qPCR with *Gapdh* as the reference gene. n = 4. NT, non-treatment. *siOdc-1* was used in all other figures unless specified otherwise.

(B-D) NIH 3T3 cells were transfected with *siOdc*-1/2 with/without 10 μM spermidine for 3 days (B/C), transfected with *siEif5a*-1/2 for 3 days (D), or treated with DFMO for 24 h (B). LC3-II was measured by Western blot. n = 3-5.

(E/F) The knockdown of *Eif5a* was induced by 100 μM IPTG in NIH 3T3 cells expressing IPTG-inducible *Eif5a* shRNA. The expression of LC3-II on indicated days post IPTG induction was measured by Western blot (E, without BafA1; F, with BafA1). n = 3-5.

(G) The expression of *Dohh* on indicated days post 4-OHT induced deletion was assessed by qPCR with *Gapdh* as the reference gene. n = 4.

(H) NIH 3T3 cells were transfected with *siCtrl* or siRNA targeting two different regions of *Dhs* mRNA (*siDhs*-1/2) for 3 days or treated with 100 μM GC7 for 24 h. n = 4. *siDhs-1* was used in all other figures unless specified otherwise.

(I) Spermidine-depleted NIH 3T3 cells by DFMO treatment were rescued with 10 μM spermidine alone or spermidine together with GC7 for 24 h. LC3-II was measured by Western blot. n = 3.

(J) Purified murine B cells were cultured with LPS for indicated days as in Figure 3H. The expression of overall eIF5A, hypusinated eIF5A, and LC3-II (without BafA1 treatment) was assessed by Western blot. Representative of 4 independent repeats.

(K) GC7 inhibits autophagy in an mTOR-independent manner (not via activating mTOR). LPS-activated murine B cells were treated with GC7 for 24 h as in Figure 3I, or with the mTOR inhibitor Torin 1 for 2 h. The expression of S6 and its phosphorylation (Ser235/236) downstream of mTOR were assessed by Western blot. n = 5.

BafA1 was added 2 h before harvest where indicated for autophagy measurement. Data represented as mean ± SEM. One-way ANOVA with post hoc Dunnett's test (A/B/D/H), or with post hoc Tukey's test (C/I). Two-way ANOVA with post hoc Sidak's test (E/F) or Dunnett's test (G), Student's t-test (K). $^{*}P \leq 0.05$, $^{**}P \leq 0.01$, $^{***}P \leq 0.001$, $^{****}P \leq 0.0001$.

Figure S5. Related to Figure 4 and Figure 5. Hypusinated eIF5A Regulates TFEB Expression and Overall Protein Synthesis.

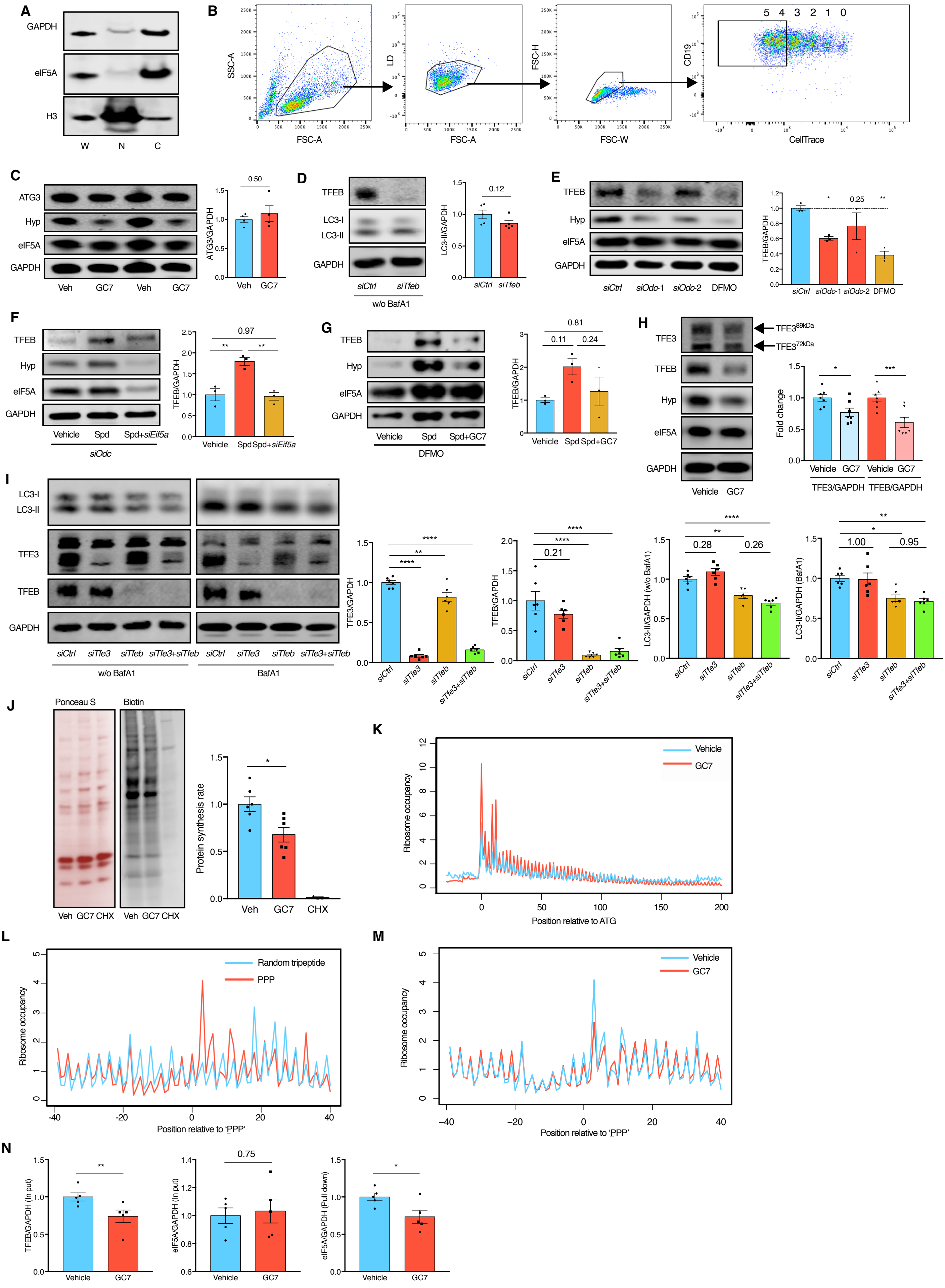

Figure S5. Related to Figure 4 and Figure 5. Hypusinated eIF5A Regulates TFEB Expression and Overall Protein Synthesis.

(A) An aliquot of protein samples for MS in Figure 4A was assessed by Western blot for cell fractionation efficiency. W: whole cell lysate; N: nuclear fraction; C: cytoplasmic fraction.

(B) Gating strategy for the FACS sorting in Figure 4B. Live B cells ($CD19^+$) that divided four times or more were collected.

(C) Murine B cells were treated with 10 μM GC7 for 24 h as in Figure 3I. The expression of ATG3 was assessed by Western blot. n = 4 mice.

(D) NIH 3T3 cells were transfected with *siTfeb* for 3 days. LC3-II expression was measured by Western blot. n = 5.

(E) NIH 3T3 cells were transfected with *siOdc*-1/2 for 3 days or treated with 1 mM DFMO for 24 h. TFEB expression was measured by Western blot. n = 3.

(F) Spermidine-depleted NIH 3T3 cells by *siOdc* transfection were rescued with spermidine alone or in combination with *siEif5a* for 3 days. n = 3.

(G) DFMO-treated NIH 3T3 cells were treated (rescued) with spermidine alone or spermidine together with GC7 for 24 h. n = 3.

(H) Murine B cells were treated with GC7 as in (C). The expression of TFE3 and TFEB was assessed by Western blot. n = 7 mice.

(I) NIH 3T3 cells were transfected with *siTfe3*, *siTfeb*, or both together for 3 days. The expression of TFE3, TFEB, and LC3-II was assessed by Western blot. n = 6.

(J) Murine B cells were treated with GC7 as in (C). Nascent proteins were labeled with AHA for 4 h, conjugated to biotin by click reaction and directly assessed by Western blot with IRDye-conjugated streptavidin. Ponceau S staining of total proteins was used as the loading control. Cycloheximide (CHX) was added 30 min before adding AHA to inhibit translation as the positive control. n = 6 mice.

(K-M) Murine B cells were treated with GC7 as in (C) and processed for ribosome profiling. (K) Ribosome occupancy of all genes is aligned at the start codon and normalized to a mean value of 1 for each gene. (L/M) Ribosome occupancy of all genes expressing PPP motifs of the Vehicle sample (L) or GC7 and Vehicle samples (M) is aligned at the underlined Pro on the P site of the ribosome and normalized to a mean value of 1 for each gene.

(N) Quantification of Figure 5B. n = 5 mice.

Data represented as mean ± SEM. Student's t-test (C/D/J/N). One-way ANOVA with post hoc Dunnett's test (E/I TFE3, TFEB), Tukey's test (F/G/I LC3-II), or Sidak's test (H). *$P \leq 0.05$, **$P \leq 0.01$, ***$P \leq 0.001$, ****$P \leq 0.0001$.

Figure S6. Related to Figure 6. Hypusination of eIF5A is Essential for Hematopoiesis and B Cell Activation.

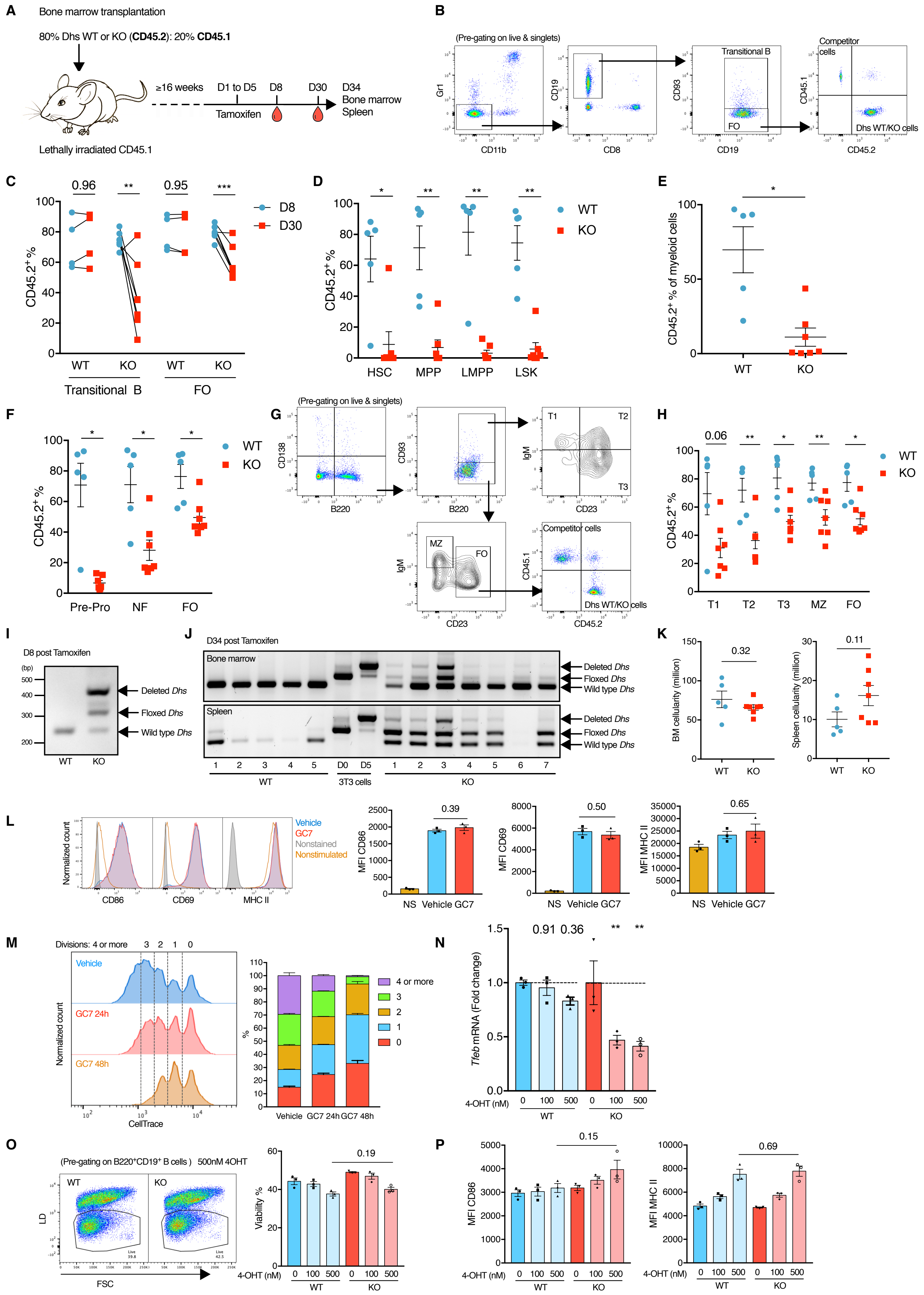

Figure S6. Related to Figure 6. Hypusination of eIF5A is Essential for Hematopoiesis and B Cell Activation.

(A-K) Competitive bone marrow chimeric mice were generated by transplanting bone marrow cells from tamoxifen-inducible CD45.2^{+} *Dhs* knockout mice (WT: *CAG-Cre/Esr1*$^{+}$, *Dhs*$^{+/+}$; KO: *CAG-Cre/Esr1*$^{+}$, *Dhs*$^{f/f}$,) and wild type CD45.1^{+} competitors into CD45.1^{+} recipient mice (A). After ≥ 16 weeks of long-term reconstitution, tamoxifen was administered by oral gavage for 5 consecutive days, followed with lineage contribution assessment. (B/C) The contribution of CD45.2^{+} cells to transitional (CD19^{+}CD93^{+}) and mature follicular (FO) B cells (CD19^{+}CD93^{-}) in peripheral blood on day 8 and day 30 post tamoxifen induction was assessed by flow cytometry. (D-H) The contribution of CD45.2^{+} cells to bone marrow hematopoietic stem and progenitor cells (D), spleen myeloid cells (CD11b^{+}Gr1^{+}) (E), bone marrow pre-pro B cells (Hardy fraction A), newly formed B cells (NF, fraction E), follicular B cells (FO, fraction F) (F), and spleen transitional B cells (T1-3), marginal zone B cells (MZ), follicular B cells (G/H) was assessed by flow cytometry on day 34 post tamoxifen induction. (I/J) *Dhs* deletion efficiency was tested by PCR. DNA was extracted from pooled peripheral blood of the chimeric mice on day 8 post tamoxifen administration (I) or from bone marrow and spleen on day 34 post tamoxifen administration (J). 4-OHT-induced *Dhs* deletion (D0/D5) in 3T3 cells derived from *CAG-Cre/Esr1*$^{+}$, *Dhs*$^{f/f}$ mouse embryonic fibroblasts was used as positive control. (K) The cellularity of bone marrow (tibia and femur of both sides) and spleen of WT and KO *Dhs* chimeric mice. n = 4-7 mice.

(L) Wild type murine B cells were stimulated with LPS for 1 day with or without 10 μM GC7. The expression of early activation markers CD86, CD69, and MHC II was assessed by flow cytometry. NS, nonstimulated. n = 3 mice.

(M) Wild type murine B cells were stained with CellTrace and cultured with LPS for 3 days. GC7 was added 24 h or 48 h before harvest for cell proliferation analysis (left). The percentage of cells that divided for the indicated times is quantified (right). n = 6 mice.

(N-P) The knockout of *Tfeb* in B cells was induced as in Figure 6A for 4 days. (N) The *Tfeb* knockout efficiency was assessed by qPCR with *Gapdh* as the reference gene. (O) The viability of B cells was assessed by flow cytometry. (P) Quantification of Figure 6C. n = 3 mice.

Data represented as mean ± SEM. Two-way ANOVA with post hoc Sidak's test for transitional B cells and follicular B cells separately (C) or with post hoc Dunnett's test (N). Welch's t-test (D/E/F/H). Student's t-test (K/L/O/P). *P≤0.05, **P≤0.01, ***P≤0.001.

Figure S7. Related to Figure 7. Spermidine does not Improve Young Human B Cell Responses.

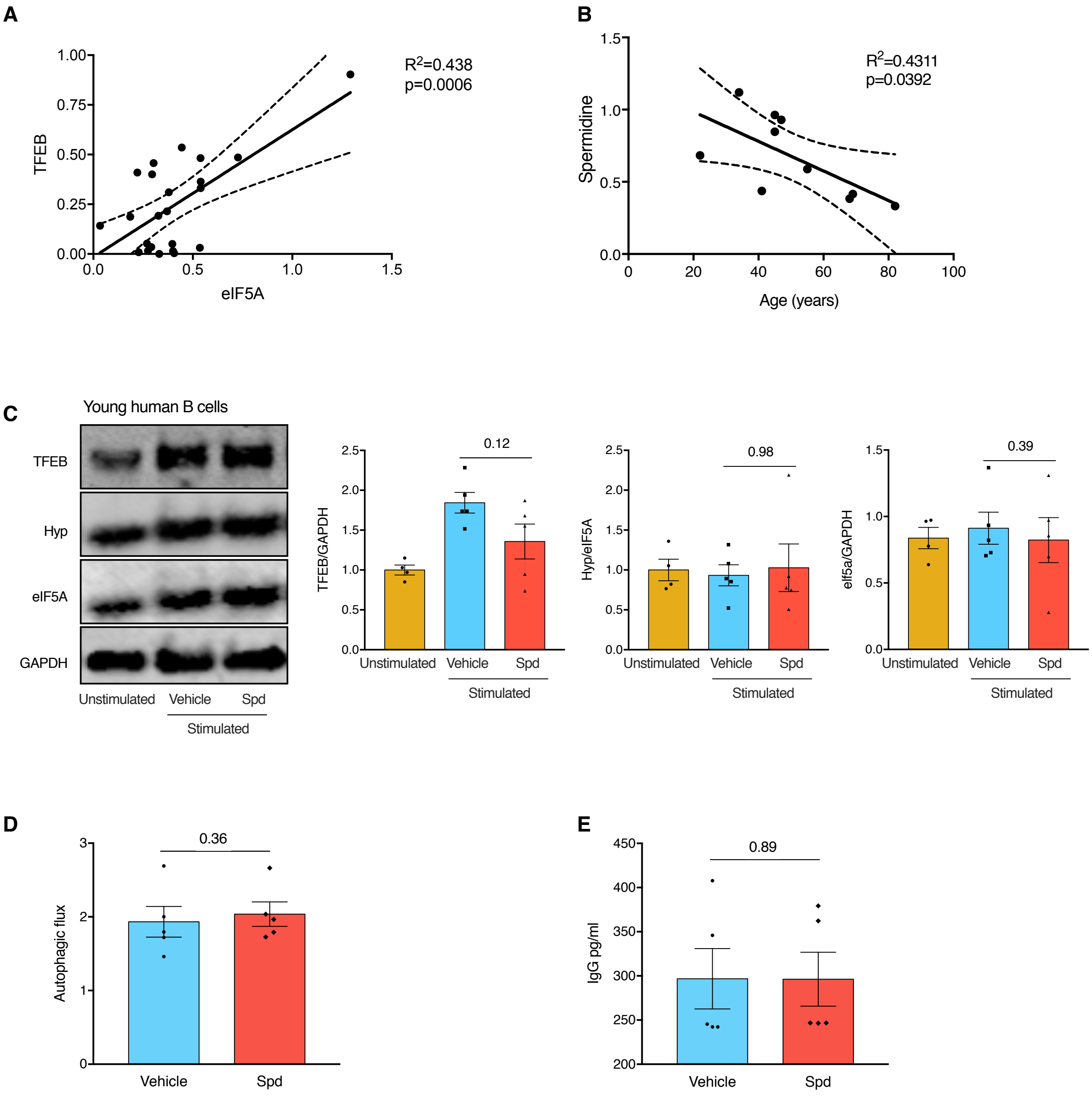

Figure S7. Related to Figure 7. Spermidine does not Improve Young Human B Cell Responses.

(A) Correlative analysis between the expression of TFEB and eIF5A (both normalized to GAPDH) in samples of Figure 7A. n = 23 donors.

(B) Spermidine content of PBMCs collected from healthy donors was measured by GC-MS. n = 10 donors.

(C-E) Sorted B cells from young human donors (<65 years) were stimulated by anti-IgM and CD40L for 7 days with 10 μM spermidine where indicated. (C) The expression of TFEB, overall eIF5A and hypusinated eIF5A was assessed by Western blot. (D) The autophagic flux was measured by flow cytometry staining of LC3-II as in Figure 1A. (E) Supernatant IgG was assessed by ELISA. n = 5 donors. Data represented as mean ± SEM.

Linear regression with 95% confidence intervals (A/B). The goodness of fit is assessed by R^2, and the P value of the slope (whether significantly non-zero) is calculated by F test (A/B). Paired Student's t-test (C-E).
